# A Survey of Strategies to Modulate the Bone Morphogenetic Protein Signaling Pathway: Current and Future Perspectives

**DOI:** 10.1155/2016/7290686

**Published:** 2016-06-28

**Authors:** Jonathan W. Lowery, Brice Brookshire, Vicki Rosen

**Affiliations:** ^1^Division of Biomedical Science, Marian University College of Osteopathic Medicine, Indianapolis, IN 46222, USA; ^2^Department of Developmental Biology, Harvard School of Dental Medicine, Boston, MA 02115, USA

## Abstract

Bone morphogenetic proteins (BMPs) constitute the largest subdivision of the TGF-*β* family of ligands and are unequivocally involved in regulating stem cell behavior. Appropriate regulation of canonical BMP signaling is critical for the development and homeostasis of numerous human organ systems, as aberrations in the BMP pathway or its regulation are increasingly associated with diverse human pathologies. In this review, we provide a wide-perspective on strategies that increase or decrease BMP signaling. We briefly outline the current FDA-approved approaches, highlight emerging next-generation technologies, and postulate prospective avenues for future investigation. We also detail how activating other pathways may indirectly modulate BMP signaling, with a particular emphasis on the relationship between the BMP and Activin/TGF-*β* pathways.

## 1. Introduction

Bone morphogenetic proteins (BMPs) constitute the largest subdivision of the TGF-*β* family of ligands. To date, approximately thirty distinct human proteins are named BMPs and some have additionally been assigned as Growth/Differentiation Factors (GDFs). However, important differences exist among these molecules with regard to pathway mechanics and effects on cellular behavior. This imprecise nomenclature can cause confusion when discussing BMP ligands and their role in human physiology or disease. Clarification may come, however, by focusing on the downstream pathway activated by each ligand rather than name alone. The intracellular effectors SMAD1/5/8 actuate the “bone morphogenetic protein” activity (i.e., autoinduction of bone at extraskeletal sites) originally described by Urist [1, 2]. Proteins that participate in the activation of SMAD1/5/8, then, are* bona fide* components of the canonical BMP signaling cascade. On this basis, it is possible to identify approximately thirteen* bone fide* BMP ligands in humans.* Bona fide* human bone morphogenetic proteins (BMPs) (less common alternative names are in parentheses) are as follows:

 BMP2 (BMP2A, BDA2A).
 BMP4 (BMP2B, BMP2B1, MCOPS6, OFC11, and ZYME).
 BMP5.
 BMP6 (VGR, VGR1).
 BMP7 (OP-1).
 BMP8A.
 BMP8B (OP-2).
 BMP9 (GDF2, HHT5).
 BMP10.
 BMP15 (GDF9B, ODG2, and POF4).
 GDF5 (BMP14, OS5, LAP4, BDA1C, CDMP1, SYM1B, and SYNS2).
 GDF6 (BMP13, KFM, KFS, KFS1, KFSL, SGM1, CDMP2, LCA17, MCOP4, SCDO4, and MCOPCB6).
 GDF7 (BMP12).

It is this narrow definition of BMP signaling that we utilize in this review article.

Bone morphogenetic proteins (BMPs) are unequivocally involved in the modulation of several stem cell populations including embryonic stem cells (ESCs), induced pluripotent stem cells, intestinal stem cells, and mesenchymal stem cells (reviewed in [3–6]). For instance, in embryonic primordial germ cell differentiation, BMP signaling activates a transcriptional network and reexpression of the pluripotency markers* Nanog* and* Sox2* [7]. Mouse ESCs also require dose dependent BMP pathway activation to maintain pluripotency [7]. Genetic inactivation studies demonstrate that* Bmp7* is essential for the maintenance of nephron progenitor cells and its absence promotes premature arrest of nephrogenesis [8]. Additionally, complete removal of BMP signaling sends inactive hair follicle (HF) stem cells into premature proliferation while ectopic expression of BMP4 reduces HF induction and leads to baldness [9]. These findings support the idea that BMP signaling acts as a gatekeeper in stem cells preventing execution of differentiation programs; however other studies demonstrate that BMPs may also elicit the opposite effect. This is often accomplished in collaboration with other signaling pathways. For example, in human ESCs BMPs work in concert with FGF2 to drive mesendoderm differentiation into cardiac, hematopoietic, pancreatic, and liver lineages [10]. The same study suggests that cells derived from mouse ESCs further differentiate into hematopoietic mesoderm cells driven by cooperation between BMP, TGF-*β*, and Wnt signals [10]. And, BMP pathway activation is a potent activator of osteochondral differentiation in mesenchymal stem cells [11]. Thus, depending on the stem cell population in question, BMP signaling may act in a context-specific manner to either stimulate differentiation or promote maintenance of pluripotency.

This widespread yet context-dependent role of BMP signaling in modulating stem cell behavior requires appropriate regulation of BMP signaling for the development and homeostasis of numerous human organ systems [12]. Aberrations in the BMP pathway or its regulation are increasingly associated with diverse human pathologies (reviewed in [13–16]). Concomitant with this increased clinical significance, there is a growing need to develop effective strategies that modulate BMP signaling as a means of regulating stem cell populations. Tremendous gains have been made in recent years, but these exciting advances have often occurred within areas that may have been overlooked by nonspecialists. Thus, in this review we wish to provide a wide-perspective on the modulation of BMP signaling by paying particular attention to strategy rather than specific application* per se*, though numerous reported applications are noted in the main text and supplemental tables. We briefly outline the current FDA-approved approaches, highlight emerging technologies, and postulate prospective avenues for future investigation. We also detail how activating other pathways may indirectly modulate BMP signaling, with a particular emphasis on the relationship between the BMP and Activin/TGF-*β* pathways.

## 2. Strategies to Activate the BMP Pathway

**Figure 1:**
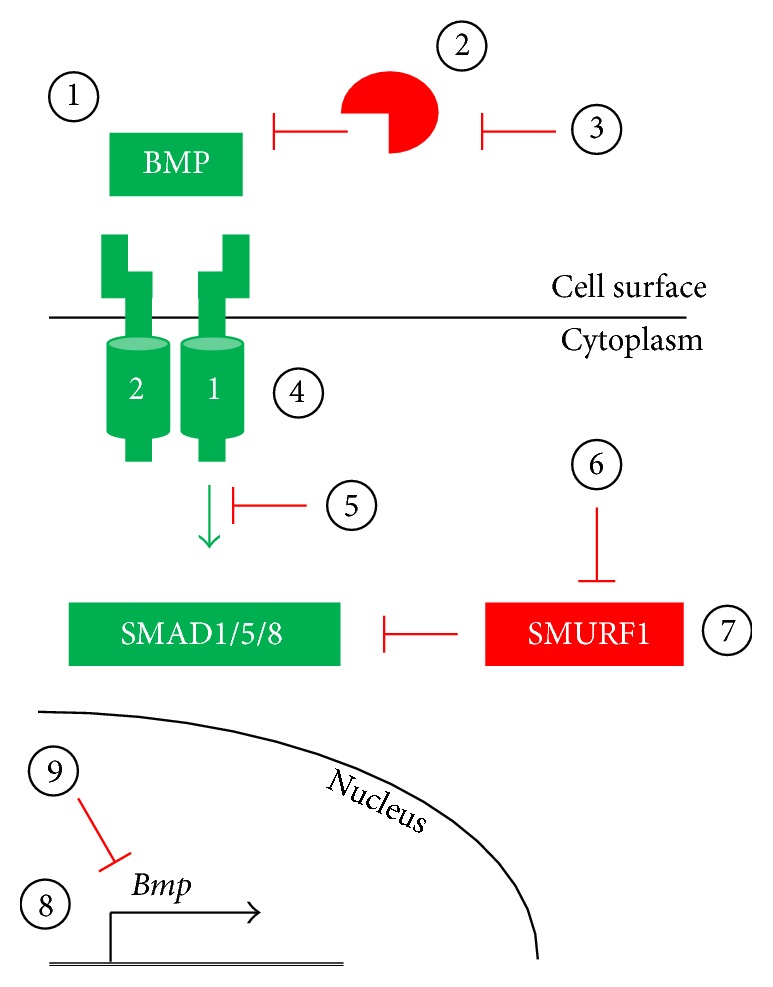
Potential strategies for modulating the BMP pathway. (1–3) The BMP pathway may be activated by exogenous natural or engineered BMP ligands or by expression of such ligands via gene transfer techniques (1). Ligand-induced BMP pathway activation may be inhibited by extracellular ligand traps, such as naturally-occurring antagonists or neutralizing antibodies, via delivery of recombinant protein or expression via gene transfer techniques (2). Endogenous extracellular BMP antagonists, such as Noggin or Chordin, may be inhibited via neutralizing antibodies or small molecules, resulting in increased BMP signaling (3). (4-5) The endogenous BMP pathway inhibitors FKBP12 and Casein Kinase 2 may be inactivated by delivery of FK506 and CK2.3, respectively, thereby increasing signal transduction (4). Alternatively, BMP receptor-mediated activation of the SMAD effectors may be blocked by kinase inhibitors (5). (6-7) Persistence of BMP signaling may be modulated by regulating the SMURF1-mediated ubiquitination of SMAD effector proteins by disrupting SMURF1 interaction with SMADs by small molecule inhibitors (6) or by increasing SMURF1 protein levels (7). (8-9) BMP pathway component expression may be elevated by increasing transcription or alleviating microRNA-mediated translational silencing (8). Alternatively, BMP pathway component levels may be reduced by reducing transcription and/or translation rates (9).

In this section, we highlight several strategies to activate the BMP pathway. These different approaches are schematized in Figure 1.

### 2.1. Natural and Engineered Ligands

The potential for clinical application of the BMP pathway was discovered decades prior to the identification of the BMP ligands [1, 2]. In these original reports, BMP activity liberated from the bone matrix was shown to promote ectopic bone formation. Several osteogenic proteins were then cloned, expressed as recombinant human proteins, and demonstrated to induce bone formation [17], heralding the potential for clinical applicability in orthopedics, which came to actualization in 2001 when recombinant human (rh) BMP7 (OP-1, Stryker) received a humanitarian device exemption (HDE) from the US FDA “for use as an alternative to autograft in recalcitrant long bone nonunions where use of autograft is unfeasible and alternative treatments have failed” (FDA). This was followed in 2002 when rhBMP2 (InFuse Bone Graft, Medtronic) received FDA medical device approval for use in anterior lumbar interbody fusion. The FDA subsequently approved rhBMP2 for use in several additional spine fusion approaches. rhBMP7 received a second HDE in 2004 for use in posterolateral lumbar fusion, and rhBMP2 received additional FDA approval for use in open tibial fractures in 2004 and oral-maxillofacial applications including sinus augmentation and localized alveolar ridge augmentation in 2007 (FDA). Several ongoing or upcoming clinical trials evaluate the usefulness of rhBMP2 and rhBMP7 in additional orthopedic/dental applications (https://clinicaltrials.gov/).

Recombinant BMPs have a high production cost for clinical use, which raises concern about their cost-effectiveness [18, 19]. As detailed in Table 1, this has prompted several groups to produce relatively short biomimetic peptides and/or to optimize BMP sequences for synthesis in* E. coli* [20–40]. Additionally, numerous studies have demonstrated the feasibility of a gene transfer approach for production of natural or engineered BMP ligands* in vivo* (Tables S1–S7 in Supplementary Material available online at http://dx.doi.org/10.1155/2016/7290686). Several of these studies accomplished cell type specific and/or regulated BMP synthesis. One very interesting idea put forth involves ingesting bacteria that express BMPs for localized production in the gastrointestinal tract [41], which might be advantageous for treating conditions like inflammatory bowel disease (Table S7).

Part of the high cost of rhBMPs is related to the fact that large amounts of protein have been required for clinical use, leading multiple groups to engineer versions that have higher activity than the naturally-occurring ligand (Table 1). For instance, BMP2 chimerae containing segments from Activin A have been shown to be resistant to sequestration by the antagonist Noggin [35, 42–47], leading to greater signaling activity. Noggin-resistant versions of BMP7 and GDF5 bearing enhanced activity have also been described [48–50]. Other studies have utilized nonsignaling ligand decoys to neutralize Noggin [51–53] or potentiate receptor complex assembly [54–59]. In addition, heterodimeric ligands, such as BMP2/6, BMP2/7, and BMP4/7, have been designed to optimize receptor:ligand interactions and each of these display greater activity than the respective homodimer [60–70]. To the best of our knowledge, there are no ongoing clinical trials in humans with these second-generation ligands. One can envision combining the best features of these intelligently engineered molecules and/or production methods into an optimized BMP pathway activator best-suited for specific clinical uses.

### 2.2. Neutralizing Antibody and Small Molecule Approaches

BMP pathway activation is regulated by a large number of soluble antagonists [71]. Because these proteins operate in the extracellular space, they are attractive targets for strategies aimed at blocking their interaction with BMPs. The feasibility of this approach has been demonstrated by studies using neutralizing antibodies against Noggin or Gremlin in the contexts of pulmonary arterial hypertension (PAH) and spinal cord injury [72–74]. Additionally, the peptide CK2.3 reportedly disrupts the inhibitory interaction between Casein Kinase 2 and the BMP type 1 receptor BMPR1A [75]. Similarly, an* in silico* screen has identified several compounds that could bind to Noggin to disrupt its interaction with BMP ligands [76] and lead candidates have emerged from a screen for small molecules that potentially inhibit the E3 ubiquitin ligase SMURF1 by preventing its interaction with the BMP effectors SMAD1/5 and targeting them for degradation [77–79]. We are not aware of clinical trials of these antibodies or small molecules for increasing BMP signaling* in vivo *at present. The FDA-approved immunosuppressant tacrolimus (Astellas Pharma), which is also known as FK506, activates BMP signaling by inhibiting FKBP12 and is being tested in a clinical trial for the treatment of PAH (NCT01647945).

### 2.3. Regulation of Expression and/or Potentiating Activity

Enhancing the expression of BMP pathway components could serve as a means to increase signaling. Numerous stimuli have been reported to increase expression levels of BMP ligands or receptors (Table S8). Notably, several kinds of clinically relevant physical stimuli, such as pulsed electromagnetic fields, ultrasound, and mechanical loading, can positively modulate the BMP pathway at multiple levels [80–89]. Additionally, several FDA-approved drugs have been shown to regulate expression of BMP pathway components and/or potentiate BMP signaling. For instance, the statin drugs lovastatin and simvastatin increase BMP2 expression and signaling in several cell types and* in vivo* [90–95]. BMP2 expression and signaling are also increased by the Rho-kinase inhibitor fasudil [96, 97]. Pan-phosphodiesterase inhibition with pentoxifylline or selective inhibition with rolipram or sildenafil has been reported to potentiate BMP signaling as well [98–104].

Recent years have brought considerable attention to the role that microRNAs (miRNAs) play in gene expression, and several miRNAs have been implicated in negatively regulating the expression of BMP pathway components (Table 2 and Section 3). This opens the door, then, to an RNA interference strategy called “anti-miR” or “antagomiR” that targets miRNA and thereby alleviates translation repression. To date, a handful of studies have demonstrated the feasibility of anti-miRs to augment BMP pathway activity* in vitro* and in animal models (Table 2). This technology could prove useful as a means to increase expression of BMP pathway members, especially in scenarios where abnormal miRNA expression is involved in disease pathogenesis [105].

## 3. Strategies to Inhibit the BMP Pathway

In this section, we will highlight several strategies to inhibit the BMP pathway. These different approaches are schematized in Figure 1.

### 3.1. Natural and Engineered Antagonists and Small Molecule Inhibitors

The fact that BMP ligands are present in the extracellular environment makes them vulnerable to sequestration upstream of receptor binding on target cells, and the extracellular antagonists Noggin, Gremlin, and Chordin might be used to regulate BMP signaling in this manner [71]. Numerous studies have exploited this relationship by administering recombinant BMP antagonists or delivering them via gene transfer (Tables S2, S4, and S6–S8). Once delivered, these antagonists typically sequester multiple BMP isoforms, which, depending on the specific application, may be advantageous or not. An alternative approach to enhance BMP:BMP antagonist interactions would be to employ soluble decoy receptors that comprise only the ligand binding domain of individual BMP receptors and, therefore, interact with ligands according to particular affinities (Table 3). An example of this kind of specificity can be observed with the soluble ALK1 (ALK1-ECD, Dalantercept, Acceleron Pharma), which is currently in clinical trials as a cancer therapy (NCT01458392, NCT01642082, NCT01720173, NCT01727336, and NCT02024087); ALK1-ECD preferentially sequesters BMP9 and BMP10 [106–111]. Greater specificity in ligand sequestration may also be achieved by using neutralizing antibodies raised against individual BMP ligands (Table 3). Investigators should be aware, however, that a high degree of homology exists between certain BMP ligands, such as BMP2 and BMP4 which are 92% identical, and this could make it challenging to specifically neutralize only one isoform when others are present. It is possible, also, that a specific BMP ligand could be inactivated via interaction with its prodomain [112] or via bespoke DNA aptamers [113].

BMP receptors are serine/threonine kinases, which makes them attractive targets for small molecules that block the kinase pocket and inhibit their activity. Considerable attention has been focused upon type 1 BMP receptors (ALK1/2/3/6) and the first kinase inhibitor reported was Dorsomorphin [114]. Though significant off-target effects are now noted for Dorsomorphin (Table 4), this molecule represents a key advancement in the field and has served as a guide for subsequent generations of analogues with greater specificity (Table 4). Some type 1 receptor selectivity has been reported among each of these compounds and it is conceivable that, in the near future, an investigator may be able to choose the most appropriate small molecule for a given application. For instance, activating mutations in ALK2 cause both fibrodysplasia ossificans progressiva (FOP) and pediatric intrinsic diffuse glioma (PIDG) [115–119]. Four candidate molecules, LDN-212854, LDN-214117, ML-347, and 1LWY, have recently been described as having dramatically enhanced selectivity for ALK2 (and the closely related ALK1) over the other type 1 receptors [120–123]; we are unaware of data directly comparing the* in vivo* efficacy of these four molecules head-to-head. Similarly, Tsugawa et al. concluded that differential type 1 receptor targeting underlies the finding that LDN-193189, DMH2, and VU5350 are effective in promoting liver regeneration in a rodent model while 1LWY is not [120].

It should be noted that some of these small molecules also target type 2 BMP receptors BMPR2, ACVR2A, and ACVR2B (Table 4), which might be advantageous in some experimental designs but could be problematic in others. And, given that ACVR2A and ACVR2B are also utilized by Activin and Activin-like ligands such as Myostatin, one must also keep in mind that Dorsomorphin and LDN-193189 can effectively block SMAD2/3 activation by these ligands [124].

### 3.2. Regulation of Expression

As mentioned in Section 2, several miRNAs have been shown to negatively regulate the expression of BMP pathway components (Table 2). In particular, translation of the BMP effector SMAD1 is repressed by at least four distinct miRNAs. And, some miRNAs, such as miR-155, target both SMAD1 and SMAD5. This raises the possibility that gene transfer of certain miRNA sequences singly or in combination could be useful as a means to impair effectors of the canonical BMP response. Proof of principle for this approach is found in several studies that utilized viral transduction or naked DNA delivery of miRNA to impact BMP signaling (Table 2). Similarly, knockdown of BMP pathway components as a means of reducing signaling* in vivo *has been accomplished by gene transfer in multiple scenarios and by various methods (Tables S2, S4, and S6). Notably, one emerging gene therapy strategy uses allele-specific RNA interference (ASP-RNAi) to selectively silence a single protein isoform, such as a constitutively active (ca) mutant [125]. Two separate groups have applied ASP-RNAi to the BMP pathway* in vitro *to knock down disease-causing caALK2 expression [126, 127]. This strategy is particularly amenable to FOP because the same point mutation underlies the vast majority of cases, thus enabling a single set of validated siRNAs to treat most patients [128]. ASP-RNAi could potentially be applied to disease-causing dominant negative mutations as well, such as those in* BMPR2* that are found in some heritable PAH patients and are associated with earlier onset and more severe disease than nonexpressed mutants [129].

In comparison to stimuli that positively modulate the BMP pathway, relatively few agents have been described to reduce expression and/or pathway activity (Table S9). Notably, the FDA-approved antianginal drug perhexiline reduces BMP signaling* in vitro* and decreases ossification in an ectopic assay [130]. BMP inhibition is also observed with a retinoic acid receptor-gamma agonist and a clinical trial is currently underway to examine this approach in reducing heterotopic ossification among patients with classic FOP (https://clinicaltrials.gov/).

## 4. Indirect Modulation of BMP Pathway Activity via Activating Other Pathways

A large body of literature describes effects on the BMP pathway when other signaling pathways are targeted. Many of these studies were designed to augment BMP signaling, especially in orthopedic and dental applications (Table S1) though other scenarios have also been evaluated (Tables S2–S7) and several ways that the cellular or tissue microenvironment can be altered to be more permissive to BMP signaling have come to light. One example of this is the synergy observed when intermittent parathyroid hormone therapy is combined with BMP2 or BMP7 in bone healing [131, 132].

Relatively little is known about how activating a different pathway can antagonize the effects of BMP signaling* in vivo*. One significant exception to this is the wide range of contexts in which the Activin/TGF*β* and BMP pathways elicit distinctly opposing effects on the same cell type. Some examples of this includes early body patterning [133], angiogenesis [134], cell fate of type 2 alveolar epithelial cells [135], maintenance of epithelial cell polarity [136], and regulation of skeletal muscle mass [137, 138]. Also, imbalances in the ratio of TGF*β* superfamily cytokines are increasingly associated with human diseases, including pulmonary and kidney fibrosis [139, 140], glaucoma [141, 142], asthma [143], and pulmonary arterial hypertension [144, 145]. This raises the intriguing possibility that the effects of Activin/TGF*β* pathway inhibition, for example, on skeletal muscle mass or bone volume, could in part be due to reducing antagonism of the BMP pathway. Support for this idea comes from the fact that increasing the BMP pathway can have similar effects to inhibiting TGF*β* signaling (e.g., [146–148]). While the Activin/TGF*β* receptor kinase inhibitor SB431542 has been reported to increase BMP signaling in preosteoblasts [149] and BMP target gene expression in chondrocytes [150], most studies have not evaluated how modulating the BMP pathway alters transduction of the Activin/TGF*β* pathway, or vice versa, so the extent to which this bidirectional antagonism impacts development and disease is not presently known. That said, in general, all cell types examined to date have the capacity to respond to BMPs, Activins, and TGF*β*s and these molecules are often present in the extracellular environment at the same time. Thus, how cells integrate BMP versus Activin/TGF*β* information and make specific decisions is an important area for future research.

## 5. Methods

Studies germane to this topic were identified in http://pubmed.com/ by combining the following search terms: antagonism; antagonist; bmp; bone morphogenetic protein; gene therapy; inhibition; inhibitor; siRNA. Articles retrieved were indexed to MEDLINE prior to January 6, 2016. Clinical trials were identified on https://clinicaltrials.gov/ and https://www.clinicaltrialsregister.eu/ prior to January 21, 2016. Specific applications highlighted are meant to be representative rather than exhaustive of the field and no endorsement by the authors of any particular application should be inferred.

## Supplementary Material

Specific reports of BMP pathway modulation and related applications are provided in the supplemental material. Specific applications highlighted are meant to be representative rather than exhaustive of the field and no endorsement by the authors of any particular application should be inferred.

## Figures and Tables

**Table 1:** Examples of engineered BMP pathway activators.

Category	Engineered version	Modification(s)	Reference(s)
BMP2-based	B2A (B2A2-K-NS)	BMP2-based peptide with heparin-binding domain that augments activity of BMP2 but has no signaling ability alone	[54–59, 151]
	BMP2-L51P	BMP2 mutant that augments activity of BMP2 but has no signaling ability alone	[51–53]
	BMP2_108	BMP2-based peptide; mimics activity of BMP2	[20]
	mBMP	BMP2-based peptide with mineral-binding domain; mimics activity of BMP2	[21]
	OPD	BMP2-based peptide; mimics or presumed to mimic activity of BMP2	[22]
	P1	BMP2-based peptide; mimics or presumed to mimic activity of BMP2	[23]
	P2	BMP2-based peptide; mimics or presumed to mimic activity of BMP2	
	P24	BMP2-based peptide; mimics or presumed to mimic activity of BMP2	[24, 25]
	PEP7	BMP2-based peptide; mimics or presumed to mimic activity of BMP2	[26]
	Unnamed	BMP2-based peptide; mimics or presumed to mimic activity of BMP2	[27–34]

BMP2/Activin A chimerae	AB204	Segmental-chimera of BMP2 and Activin A with enhanced activity over BMP2; Noggin resistant	[35, 42–46]
	AB204-I103Y	Variant of AB204; enhanced activity over BMP2 and AB204	[42]
	AB211	Segmental-chimera of BMP2 and Activin A with enhanced activity over BMP2; Noggin resistant	[35]
	AB215	Segmental-chimera of BMP2 and Activin A with enhanced activity over BMP2; Noggin resistant	[35, 47]

BMP2/BMP9 chimera	BB29	Segmental-chimera of BMP2 and BMP9 with enhanced folding when produced in *E. coli*	[35]

BMP6/BMP7 chimera	80-1	Segmental-chimera of BMP6 and BMP7 with reduced Noggin binding when compared to BMP7	[48]

BMP7-based	BMP7-E60K	BMP6-informed mutant with reduced Noggin binding	[48]
	THR-123	BMP7-based peptide	[36]
	Unnamed	BMP7-based peptide; mimics activity of BMP7	[27]

BMP9-based	MB109	BMP9-based peptide optimized for production in *E. coli*	[37]
	pBMP9	BMP9-based peptide with enhanced activity over BMP9	[38–40]
	SpBMP9	BMP9-based peptide with enhanced activity over BMP9	[40]
	Unnamed	BMP9-based peptide; mimics activity of BMP9	[27]

GDF5-based	GDF5-S94N	Naturally-occurring mutant with enhanced activity due to decreased inhibition by Noggin	[48]
	GDF5-N445K	Naturally-occurring mutant with enhanced activity due to decreased inhibition by Noggin	[49]
	GDF5-N445T	Naturally-occurring mutant with enhanced activity due to decreased inhibition by Noggin	[49, 50]
	GDF5-V453/V456	BMP2-informed variant of GDF5; enhanced activity over GDF5 and BMP2	[152, 153]

Heterodimers	BMP2/6	Heterodimer with enhanced activity over BMP2 and BMP6	[60, 61]
	BMP2/7	Heterodimer with enhanced activity over BMP2 and BMP7	[62–67]
	BMP4/7	Heterodimer with enhanced activity over BMP4 and BMP7	[68–70]

**Table 2:** Examples of microRNAs targeting BMP pathway components and their inhibition via anti-miR RNA interference.

miRNA	Target(s)/notes	Reference(s)	Anti-miR
miR-17-5p	*Bmpr2, Smad7*	[154, 155]	NR
miR-20a	*Bmpr2, Bambi, Crim1*	[154, 156]	[157]
miR-23b	*Smad4, Smad5*; also *Smad3*	[158]	NR
miR-26a	*Smad1, Smad4, Tob1*	[159–161]	[159, 160]
miR-27	*Acvr2a*; also *Tgfβr1* and *Smad2*	[162]	NR
miR-30a/b/c/d	*Bmp7, Smad1*	[163, 164]	[164]
miR-100	*Bmpr2*	[165]	NR
miR-122	*Hemojuvelin*	[166]	[166]
miR-125	*Bmpr2*	[167]	[167]
miR-130a	*Alk2*	[168]	NR
miR-135b	*Bmpr2, Smad5*; also *Alk4 *and *Tgfβr2*	[169, 170]	NR
miR-140	*Bmp2*	[171]	NR
miR-145	Undetermined (possibly *Bmp4* indirectly)	[172]	NR
miR-148a	*ALK2*	[173]	NR
miR-153	*Bmpr2*	[174]	NR
miR-155	*Smad1, Smad5*	[175, 176]	NR
miR-199a^*∗*^	*Smad1*	[177]	[177]
miR-200	*Bmp4, *indirectly	[178]	NR
miR-205	*Smad1*, *Smad4*	[179]	NR
miR-302	*Bmpr2*	[180]	NR
miR542-3p	*Bmp7*	[181]	NR

NR: not reported.

**Table 3:** Examples of BMP pathway modulation by receptor ECDs or neutralizing antibodies.

Molecule	Reference(s)
ACVR2A-ECD	[182]
ACVR2B-ECD	[182, 183]
Anti-ALK1 Ab	[184]
ALK1-ECD	[106–110]
ALK3-ECD	[185–188]
Anti-BMP2 Ab	[189, 190]
Anti-BMP4 Ab	[190–192]
Anti-BMP6 Ab	[193–195]
Anti-BMP7 Ab	[196, 197]
Anti-BMP10 Ab	[111]
BMPR2-ECD	[198]
Dragon-ECD	[194]
Anti-gremlin Ab	[72]
Hemojuvelin-ECD	[193, 199, 200]
Anti-noggin Ab	[73, 74]

Ab: antibody; ECD: extracellular domain.

**Table 4:** Small molecule inhibitors of BMP Type 1 receptors and examples of their use.

Molecule	Comment(s)	Reference(s)
1LWY	Dramatically enhanced selectivity for ALK2 versus other type 1 BMP receptors (approximate order of selectivity: ALK2 > ALK3 > ALK6); greatly reduced off-target effects compared to DM and LDN	[120]

DMH1	Pan-type 1 BMP receptor inhibitor (approximate order of selectivity: ALK3 > ALK1 > ALK6 > ALK2); reduced off-target effects compared to DM and LDN	[121, 122, 201–205]

DMH2	Pan-type 1 BMP receptor inhibitor (approximate order selectivity: ALK6 > ALK3 > ALK2); notable off-target effects, including BMPR2, TGFBR2, ALK4, ALK5, AMPK, and VEGFR2	[120, 201, 206]

DMH3	Presumed pan-type 1 BMP receptor inhibitor; reduced off-target effects compared to DM and LDN	[201]

Dorsomorphin (DM)	Pan-type 1 BMP receptor inhibitor (approximate order of selectivity: ALK2 > ALK3 > ALK1 > ALK6); notable off-target effects, including BMPR2, ACVR2A, ACVR2B, TGFBR2, ALK5, AMPK, VEGFR2, and PDGFR*β*	[114, 121, 122, 124, 201, 202, 207–215]

K02288	Modestly enhanced selectivity for ALK1 and ALK2 versus other type 1 BMP receptors (approximate order of selectivity: ALK2 > ALK1 > ALK6 > ALK3); reduced off-target effects compared to DM and LDN	[121, 216, 217]

LDN-193189 (LDN)	Pan-type 1 BMP receptor inhibitor (approximate order of selectivity: ALK1~ALK2 > ALK3 > ALK6); notable off-target effects, including BMPR2, ACVR2A, ACVR2B, TGFBR2, ALK5, AMPK, VEGFR2, and PDGFR*β*	[120–122, 124, 185, 191, 207–209, 216, 218–227]

LDN-212854	Significantly enhanced selectivity for ALK1 and ALK2 versus other type 1 BMP receptors (approximate order of selectivity: ALK2 > ALK1 > ALK3); reduced off-target effects compared to DM and LDN	[121]

LDN-214117	Dramatically enhanced selectivity for ALK2 versus other type 1 BMP receptors (approximate order of selectivity: ALK1, ALK2 > ALK3); greatly reduced off-target effects compared to DM and LDN	[123]

ML-347	Dramatically enhanced selectivity for ALK1 and ALK2 versus other type 1 BMP receptors (approximate order of selectivity: ALK2 > ALK1 ≫ ALK3); reduced off-target effects compared to DM and LDN	[122, 228]

VU5350	Pan-type 1 BMP receptor inhibitor (approximate order selectivity: ALK3 > ALK2 > ALK6); notable off-target effects, including BMPR2, TGFBR2, AMPK, and VEGFR2	[120]
