# Supplementary material for: A Survey of Strategies to Modulate the Bone Morphogenetic Protein Signaling Pathway: Current and Future Perspectives
Source: Stem Cells Int. 2016 Jun 28;2016:7290686. doi: 10.1155/2016/7290686 (PMC4940573; doi:10.1155/2016/7290686)
Supplement: Supplementary file 1 — Specific reports of BMP pathway modulation and related applications are provided in the supplemental material. Specific applications highlighted are meant to be representative rather than exhaustive of the field and no endorsement by the authors of any particular application should be inferred. [file 7290686.f1.pdf]

Table S1. Examples of BMP pathway modulation for orthopedic and dental applications.

Application	Agent	Technique	Modifier		Reference
			Agent	Effect of Modifier	
Articular Cartilage and Meniscal Repair	BMP2	Gene Transfer	Periosteal Cells	ND	[1]
			Chondrocytes	ND	[2]
			Sox9 + Chondrocytes	ND	[3]
	BMP4	Gene Transfer	None	NA	[4]
			ASCs	ND	[5]
			MDSCs	ND	[6]
			sFLT1 + PRP and MDSCs	Enhance	[7]
	BMP6	Recombinant	ASCs	ND	[8]
	BMP7	Recombinant	None	NA	[9, 10]
		Gene Transfer	Periosteal Cells	ND	[11]
Bone Augmentation / Grafting / Fracture Repair	AB204	Recombinant	None	NA	[12]
	caALK2	Gene Transfer	None	NA	[13]
	caALK6	Gene Transfer	Runx2	ND	[14]
	BB1	Recombinant	None	NA	[15]
	BMP2	Recombinant	None	NA	[12, 15-136]
			Alendronate	Enhance	[137]
			Antibiotic	Enhance	[138]
			ASCs	ND	[139]
				No Change	[140]
			BMP2-L51P	Enhance	[84]
			BMSCs	Enhance	[141-144]
				ND	[145]
				No Change	[146, 147]
			Smurf1-KD BMSCs	Enhance	[148]
			DBP	No Change	[149]
			EGF	Enhance	[150]
			EPO	Enhance	[151]
			bFGF	Enhance	[152, 153]
				No Change	[150, 154]
			LIPUS	Enhance	[155]
			OPG	Enhance	[156]
			PEMF	Enhance	[157]
			PDGF	Enhance	[150]
			PRP	Enhance	[158]
				No Change	[159]
			iPTH	Enhance	[160]
			Phenamil	Enhance	[161]
			SDF1	Enhance	[162, 163]
				No Change	[164]
			TGFβ2	No Change	[165]
			VEGF	Enhance	[166-168]
				No Change	[150, 169]
		Gene Transfer	None	NA	[170-188]
			ASCs	ND	[189-192]
			ASCs + miR148b	Enhance	[193]
			BMP7	ND	[194]
			BMP7 + BLKs	Enhance	[195]
			BMSCs	ND	[196-227]
			BMSCs + PDGF	Enhance	[228]
			BMSCs + EPCs	Enhance	[229]
			DFs	ND/Enhance	[171, 230]
			MDCs	ND	[231-233]
			OPG	Enhance	[156]

			PDLSCs	ND	[234]
			VEGF	ND	[235]
	B2A2	Peptide	None	NA	[236]
	BMP4	Recombinant	None	NA	[237-240]
		Gene Transfer	None	NA	[241-243]
			BMSCs	ND	[244-246]
			MDCs	ND	[245, 247]
	BMP6	Recombinant	None	NA	[248]
		Gene Transfer	None	NA	[172]
	BMP7	Recombinant	None	NA	[17, 110, 248-276]
			BMSCs	Enhance	[277]
			BBP	Enhance	[278]
			iPTH	Enhance	[279]
			TGFβ2	No Change	[280]
			ZA	Enhance	[281]
		Gene Transfer	None	NA	[170, 282-284]
			BMP2 + BLKs	Enhance	154]
			BMSCs	ND	[285]
			DFs	ND	[286-290]
			PDGF	Enhance	[291]
			Periosteal Cells	ND	[292]
	BMP9	Gene Transfer	None	NA	[173, 293]
	GDF5	Recombinant	None	NA	[294-298]
	GDF5-N445T	Recombinant	None	NA	[299]
	GDF5-V453/V456	Recombinant	None	NA	[298]
	Noggin siRNA	Gene Transfer	OBs	ND	[300]
			ASCs + BMP2	ND	[301, 302]
	P1	Recombinant	None	NA	[303]
	PEP7	Peptide	None	NA	[21]
	Smurf1 siRNA	Gene Transfer	BMSCs + BMP2	Enhance	[148]
Bone / Tooth Regeneration	BMP2	Recombinant	None	NA	[304, 305]
			Fibrin	Enhance	[306]
	BMP4	Recombinant	None	NA	[307]
Craniosynostosis	Noggin	Recombinant	None	NA	[308]
		Gene Transfer	None	NA	[309]
			MDCs	ND	[310]
Heterotopic/Ectopic Ossification	caALK2 siRNA	Gene Transfer	None	NA	[311, 312]
	BMP2	Recombinant	EP4A	Enhance	[313, 314]
			FGF2	Enhance/Decrease	[315]
			Heparin	Enhance	[316]
			PDE Inhibitor	Enhance	[317-319]
			SB-431542	No Change	[320]
			Spp24	Decrease	[321, 322]
			TNP-470	Decrease	[323]
			VEGF	Enhance	[169]
			Y-27632	Enhance	[324]
		Gene Transfer	BMP7 + BLKs	Enhance	[325]
			FK506	Enhance	[326]
			Noggin + MDCs	Decrease	[327]
			Runx2 + ASCs	Enhance	[328]
			VEGF + Periosteal Cells	Enhance	[329]
			VEGF + BMSCs	Enhance	[330]
	Corticosteroid	Small Molecule	None	NA	[331]
	LDN-193189	Small Molecule	None	NA	[331, 332]
	LDN-212854	Small Molecule	None	NA	[333]
	Noggin	Gene Transfer	None	NA	[334]
	NogginΔB2	Gene Transfer	None	NA	[334]

	Perhexiline	Small Molecule	None	NA	[335]
	RAR-γ agonist	Small Molecule	None	NA	[336]
IVD Degeneration	BMP2	Gene Transfer	None	NA	[337, 338]
	BMP7	Recombinant	None	NA	[339]
		Gene Transfer	Chondrocytes	ND	[340]
			NPCs	ND	[341]
	GDF5	Recombinant	None	NA	[342]
Joint Fusion	BMP2	Recombinant	None	NA	[109, 343-401]
			BMP7	Enhance	[402]
			COMP	Enhance	[403]
			iPTH	Enhance	[404]
			BMSCs	Enhance	[405-407]
			EP4A	Enhance	[408]
			bFGF + BMSCs	Enhance	[409]
		Gene Transfer	None	NA	[410, 411]
			ASCs	ND	[412]
			BMSCs	ND	[412-418]
			NRKs	ND	[419]
	B2A	Peptide	None	NA	[420-422]
	BMP4	Recombinant	None	NA	[423, 424]
		Gene Transfer	None	NA	[425]
	BMP6	Recombinant	None	NA	[426]
		Gene Transfer	None	NA	[427]
	BMP7	Recombinant	None	NA	[428-440]
			BBP	Enhance	[441]
			BMP2	Enhance	[402]
		Gene Transfer	BMSCs	ND	[442]
	BMP9	Gene Transfer	None	NA	[443]
			BMSCs	ND	[444]
Osteopenia / Osteoporosis	ALK3-ECD	Recombinant	None	NA	[445]
	BMP2	Recombinant	None	NA	[446-448]
		Gene Transfer	BMSCs	ND	[449, 450]
	BMP4	Gene Transfer	None	NA	[451]
			BMSCs	ND	[452]
	BMP6	Recombinant	None	NA	[453]
	BMP7	Recombinant	None	NA	[448]
Peri-implantitis	BMP2	Gene Transfer	PDLSCs	ND	[234]
Peri-trabecular fibrosis	BMP7	Recombinant	None	NA	[454]
Rubenstein-Tayibi defects	BMP2 + BMP7	Recombinant	None	NA	[455]
Tendon / Ligament Repair, Integration, Enthesitis	BMP2	Recombinant	None	NA	[456-458]
			Periosteal Cells	ND	[459, 460]
		Gene Transfer	None	NA	[461]
			SMAD8 + BMSCs	ND	[462]
			NRKs	ND	[463]
	BMP4	Gene Transfer	None	NA	[464]
	GDF5	Recombinant	None	NA	[465, 466]
		Gene Transfer	None	NA	[467]
	GDF6	Recombinant	None	NA	[465]
		Gene Transfer	None	NA	[468]
	GDF7	Recombinant	None	NA	[469, 470]
		Gene Transfer	MDSCs	ND	[471]
	Noggin	Gene Transfer	None	NA	[472]

ASCs, adipose-derived stromal cells; BBP, BMP-binding protein; bFGF, basic fibroblast growth factor; BLKs, embryonic mouse fibroblasts; BM, bone mass; BMD, bone mineral density; BMSCs, bone marrow-derived stromal cells; ca: constitutively active; DBP, vitamin D binding protein; DFs, dermal fibroblasts; ECD, extracellular domain; EGF, epidermal growth factor; EP4A, EP4 receptor agonist; EPCs, endothelial progenitor cells; EPO, erythropoietin; iPTH, intermittent

parathyroid hormone therapy; LIPUS, low-intensity pulsed ultrasound; MDCs, muscle-derived stromal cells; NA, not applicable; ND, no data (direct comparison not reported); NPCs, nucleus polposus cells; NRKs, normal rat kidney cells; OBs, osteoblasts; OPG, osteoprotegerin; PDGF, platelet-derived growth factor; PDLSCs; periodontal ligament-derived stromal cells; PEMF, pulsed electromagnetic field; PRP, platelet rich plasma; SDF1, stromal cell-derived factor 1; siRNA, small interfering RNA; VEGF, vascular endothelial growth factor; ZA, zoledronic acid.

Table S2. Examples of BMP pathway modulation for fibrosis-related applications

Application	Agent	Technique	Modifier		Reference
			Agent	Effect of Modifier	
Bone Marrow Fibrosis	BMP7	Recombinant	None	NA	[454]
Corneal Fibrosis	BMP7	Gene Transfer	None	NA	[473, 474]
Hepatic Fibrosis	BMP7	Recombinant	None	NA	[475, 476]
		Gene Transfer	None	NA	[477, 478]
Lens Fibrosis	BMP7	Gene Transfer	None	NA	[479]
	ID1	Gene Transfer	None	NA	[479]
	ID3	Gene Transfer	None	NA	[479]
Prosthesis-related Fibrosis	BMP7	Recombinant	None	NA	[276]
Pulmonary Fibrosis	BMP7	Recombinant	None	NA	[480, 481]
Renal Fibrosis	BMP2	Recombinant	None	NA	[482]
	BMP7	Recombinant	None	NA	[483-489]
		Gene Transfer	BMSCs	ND	[490]
	FK506	Small Molecule	None	NA	[491]
	Gremlin siRNA	Gene Transfer	None	NA	[492]
	SMAD7	Gene Transfer	None	NA	[493]
	THR-123	Peptide	None	NA	[494]
	Trichostatin-A	Small Molecule	None	NA	[495]

BMSCs, bone marrow-derived stromal cells; NA, not applicable; ND, no data (direct comparison not reported)

Table S3. Examples of BMP pathway modulation for (cardio)vascular applications.

Application	Agent	Technique	Modifier		Reference
			Agent	Effect of Modifier	
Angiogenesis	ALK1-Fc	Recombinant	None	NA	[496-500]
	Anti-BMP9 Ab	Recombinant	None	NA	[500]
	Anti-BMP10 Ab	Recombinant	None	NA	[500]
Hypertensive and/or Prediabetic Cardiac Remodeling	BMP2	Recombinant	None	NA	[501]
	BMP7	Recombinant	None	NA	[502, 503]
	Smad7	Gene Transfer	None	NA	[504]
Myocardial Infarction	BMP2	Recombinant	None	NA	[505]
	BMP10	Recombinant	None	NA	[506]
	Dorsomorphin	Small Molecule	None	NA	[507]
	FSLT1	Recombinant	None	NA	[508]
	Noggin	Recombinant	None	NA	[507]
	sFRP2	Gene Transfer	BMSCs	ND	[509]
Pulmonary Arterial Hypertension	BMPR2	Gene Transfer	None	NA	[510-513]
	BMP9	Recombinant	None	NA	[514]
	Chloroquine	Small Molecule	None	NA	[515]
	FK506	Small Molecule	None	NA	[516]
	Anti-Gremlin Ab	Recombinant	None	NA	[517]
	miR-20a antagomiR	Gene Transfer	None	NA	[518]
	Serotonin	Small Molecule	None	Na	[519]
	Sildenafil	Small Molecule	None	NA	[520]
Systemic Hypertension	Alk3 siRNA	Gene Transfer	None	NA	[521]
	Bmp4 siRNA	Gene Transfer	None	NA	[521]
	Noggin	Recombinant	None	NA	[521]
	Smad7	Gene Transfer	None	NA	[493]
Vascular Inflammation, Atherosclerosis, and Vascular Calcification	Noggin	Recombinant	None	NA	[522]
	ALK3-ECD	Recombinant	None	NA	[523, 524]
	BMP7	Recombinant	None	NA	[525]
	LDN-193189	Small Molecule	None	NA	[523, 524, 526, 527]
	sFRP2	Gene Transfer	BMSCs	ND	[509]

Ab, antibody; BMSCs, bone marrow-derived stromal cells; ECD, extracellular domain; NA, not applicable; ND, no data (direct comparison not reported)

Table S4. Examples of BMP pathway modulation for reproduction-related applications.

Application	Agent	Technique	Modifier		Reference
			Agent	Effect of Modifier	
Oocyte Quality	BMP6	Recombinant	None	NA	[528]
Ovulation Rate	BMP6	Recombinant	None	NA	[528]
	BMP7	Recombinant	None	NA	[529]
	Anti-BMP15 Ab	Recombinant	None	NA	[530-532]
	BMPR2-ECD	Recombinant	None	NA	[533]

Ab, antibody; ECD, extracellular domain; NA, not applicable.

Table S5. Examples of BMP pathway modulation for cancer-related applications.

Application	Agent	Technique	Modifier		Reference
			Agent	Effect of Modifier	
Breast Cancer	AB215	Recombinant	None	NA	[534]
	BMP2	Recombinant	None	NA	[535]
Breast Cancer Metastasis to Bone	BMP2	Recombinant	None	NA	[536]
	BMP2/7	Recombinant	None	NA	[537]
Breast Cancer Metastasis to Lung	DMH1	Small Molecule	None	NA	[538]
Gliobastoma	BMP4	Gene Transfer	ASCs	ND	[539]
Lung Cancer	BMP2 siRNA	Gene Transfer	A549 Cells	ND	[540]
Prostate Cancer Metastasis to Bone	Anti-BMP6 Ab	Recombinant	None	Na	[541]
	BMP7	Recombinant	None	NA	[542]
	LDN-193189	Small Molecule	None	NA	[543]
	Noggin	Gene Transfer	C42b Cells	ND	[544]
			PC-3 Cells	ND	[545, 546]
	Noggin siRNA	Gene Transfer	PC-3 Cells	ND	[547]
Tumor Angiogenesis	ALK1-ECD	Recombinant	None	NA	[496-498, 548]

Ab, antibody; ASCs, adipose-derived stromal cells; ECD, extracellular domain; NA, not applicable; ND, no data (direct comparison not reported).

Table S6. Examples of BMP pathway modulation for nervous system-related applications

Application	Agent	Technique	Modifier		Reference
			Agent	Effect of Modifier	
Intraventricular Hemorrhage	Noggin	Recombinant	None	NA	[549]
CNS Ischemia	BMP7	Recombinant	None	NA	[550-553]
		Gene Transfer	None	NA	[554]
	Noggin	Recombinant	None	NA	[555]
		Gene Transfer	BMSCs	ND	[556, 557]
Remyelination	Noggin	Recombinant	None	NA	[558, 559]
Spinal Cord Injury	Anti-Noggin Ab	Recombinant	None	NA	[560, 561]
	Noggin	Recombinant	None	NA	[562, 563]
		Gene Transfer	NPCs	ND	[564]

Ab, antibody; BMSCs, bone marrow-derived stromal cells; NA, not applicable; ND, no data (direct comparison not reported); NPCs, neural precursor cells

Table S7. Examples of BMP pathway modulation for miscellaneous applications.

Application	Agent	Technique	Modifier		Reference
			Agent	Effect of Modifier	
Airway inflammation	BMP4	Recombinant	None	NA	[565]
Inflammatory Bowel Disease	Anti-BMP6 Ab	Recombinant	None	NA	[566]
	BMP7	Recombinant	None	NA	[567, 568]
	HJV-ECD	Recombinant	None	NA	[566]
	LDN-193189	Small Molecule	None	NA	[566, 569]
Iron Deficiency Anemia	ALK3-ECD	Recombinant	None	NA	[569]
	Anti-BMP6 Ab	Recombinant	None	NA	[566, 570, 571]
	Dorsomorphin	Small Molecule	None	NA	[572]
	Heparin	Small Molecule	None	NA	[573]
	HJV-ECD	Recombinant	None	NA	[566, 570, 574, 575]
	LDN-193189	Small Molecule	None	NA	[566, 569, 574]
Liver Regeneration	DMH2	Small Molecule	None	NA	[576]
	LDN-193189				
	VU5350				
Lung Injury (Acute)	LDN-193189	Small Molecule	None	NA	[577]
Muscular Dystrophy	Noggin	Gene Transfer	None	NA	[578]
Obesity (Diet-induced)	BMP7	Recombinant	None	NA	[579]
Pancreatitis (Acute)	Noggin	Recombinant	None	NA	[580]
Retinal Injury	BMP4	Recombinant	None	NA	[581]
Rhytid	ALK3-ECD	Recombinant	None	NA	[582]
Strabismus	BMP4	Recombinant	None	NA	[583]

Ab, antibody; ECD, extracellular domain; NA, not applicable.

Table S8. Agents shown to increase expression of BMP pathway components, enhance BMP signaling, and/or act synergistically with BMP signaling in a therapeutic context.

Modifier	Effect	Reference
Acerogenin A	Increases BMP2, BMP4, and BMP7 expression	[584]
2-Acetyldibenzothiophene	Increases BMP2 expression	[585]
AST-II	Increases BMP2 expression	[586]
BBP	Enhances BMP signaling	[278, 587, 588]
Chloroquine	Stabilizes BMPR2; enhances BMP signaling	[515, 589]
Cilomilast	Enhances BMP signaling	[590]
Compound 1	Increases BMP2 expression	[591]
Compound 5	Increases BMP2 expression	[592]
Compound 8a	Increases BMP2 expression	[591]
Compound 11	Increases BMP2 expression	[593]
Coumarin	Increases BMP2 expression	[594]
Diadzein	Increases BMP2 expression	[585]
DSP	Synergy	[595]
EGF	Synergy	[150]
Epinephrine	Enhances BMP signaling	[596]
Estradiol	Enhances BMP signaling	[597]
ESW	Increases BMP2 expression	[598]
Fasudil	Increases BMP2 expression and enhances BMP signaling	[599, 600]
bFGF	Synergy	[152, 315, 409]
Formononetin	Increases BMP2 expression	[585]
FSAP	Increases BMP signaling by activating BMP2	[601]
Genistein	Increases expression of several BMPs	[602]
Harmine	Increases BMP2, BMP4, BMP6, and BMP7 expression	[603]
HDAC Inhibitor	Enhances BMP signaling	[604]
Heparin sulfate	Enhances BMP signaling	[316, 605]
HGF	Increases BMP receptor expression	[606]
HSE	Increases BMP7 expression	[607]
HSP70	Enhances BMP signaling by binding MGP	[608]
Icariin	Increases BMP2 expression	[609]
IGF-1	Synergy	[610]
IGF-2	Enhances BMP signaling	[611]
Iron	Increases BMP6 expression	[612]
Lactoferrin	Increases BMP signaling by downregulating Noggin and/or increasing BMP7 expression	[613, 614]
Licochalcone A	Enhances BMP signaling	[615]
LIPUS	Increases BMP2, BMP4, BMP6 and BMP7 expression; enhances activity	[155, 616-619]
LLLI	Increases BMP2 expression and enhances activity	[620]
Lovastatin	Increases BMP2 expression	[621]
Mechanical Stress	Increases BMP2, BMP6 and BMP7 expression and enhances signaling	[622-624]
Melatonin	Increases BMP2 and BMP4 expression	[625]
miR-148b	Synergy	[193]
Myricetin	Increases BMP2 expression	[626]
NMP	Enhances BMP signaling	[627]
NOV	Increases BMP4 expression	[628]
ONO-4819	Enhances BMP activity and BMP2 expression	[313, 314, 408, 629, 630]
PTH	Synergy; enhances BMP signaling and increases BMP2 expression	[160, 631-633]
PDGF-BB	Synergy	[228, 291]
Phenamil	Enhances BMP signaling by promoting SMURF1 degradation	[634]
Phosphoserine	Increases BMP2 expression	[635]
PEMF	Synergy; increases BMP2 and BMP7 expression	[636-638]
Pentoxifylline	Enhances BMP activity	[318]
Resveratrol	Increases BMP2 and BMP7 expression and BMP signaling	[639, 640]
9-cis Retinoic acid	Increases BMP7 expression	[641, 642]

all-trans Retinoic acid	Synergy	[643]
RGD peptide	Synergy	[644]
Rolipram	Enhances BMP signaling	[317, 319, 590]
Runx2	Synergy	[328]
Salidroside	Increases BMP2, BMP6, and BMP7 expression	[645]
Serotonin	Possibly activates BMP signaling	[519]
Sildenafil	Enhances BMP signaling	[520, 646-648]
Silibinin	Increases BMP2 expression	[649]
Simvastatin	Increases BMP2 expression	[621, 650-654]
SMURF1 Inhibitors	Enhances BMP signaling	[655-657]
Sox9	Synergy	[3]
Syringetin	Increases BMP2 expression	[658]
TGFβ	Synergy	[659, 660]
Titanium Particles	Synergy	[661]
U0126	Increases BMP signaling and possibly BMP2 expression	[662]
VEGF	Synergy	[166, 167, 329, 330]
Y-27632	Enhances BMP signaling and BMP4 expression	[324]
Zoledronic acid	Synergy	[281]

AST-II, astragaloside II; BBP, BMP-binding protein; DSP, dentin sialoprotein; EGF, epidermal growth factor; ESW, extracorporeal shock wave; HDAC, histone deacetylase; HGF, hepatocyte growth factor; HSE, Hwanggeumchal sorghum extract; HSP70, heat shock protein 70; IGF, insulin-like growth factor; LIPUS, low-intensity pulsed ultrasound; LLLI, low-level laser irradiation; MGP, matrix-GLA protein; NMP, N-methyl pyrrolidone; PEMF, pulsed electromagnetic field; PTH, parathyroid hormone; VEGF, vascular endothelial growth factor.

Table S9. Agents shown to decrease expression of BMP pathway components, reduce BMP signaling, and/or act antagonistically with BMP signaling in a therapeutic context.

Modifier	Effect	Reference
COX-2 inhibitor	Reduces BMP6 expression	[663]
FGF2	Reduces BMP signaling	[664]
IGF-1	Reduces BMP signaling	[665]
IL-6	Reduces BMPR2 expression	[666]
LPS	Reduces BMP signaling	[667]
Perhexiline	Reduces BMP signaling	[335]
NF-κB	Reduces BMP signaling	[667]
Progesterone	Reduces BMP7 expression	[668]
PTHrP	Reduces BMP6 expression	[669]
Rapamycin	Reduces BMP signaling	[670]
Retinoic acid	Reduces BMP signaling	[671]
RAR-γ agonist	Reduces BMP signaling	[336]
TNP-470	Reduces BMP activity	[323]

COX, cyclooxygenase; FGF, fibroblast growth factor; IL, interleukin; LPS, lipopolysaccharide; NF-κB, nuclear factor kapp-light-chain-enhance of activated B cells; PTHrP, parathyroid hormone-related peptide; RAR, retinoic acid receptor.

Supplemental References:

1. Gelse, K., et al., *Articular cartilage repair by gene therapy using growth factor-producing mesenchymal cells.* Arthritis Rheum, 2003. **48**(2): p. 430-41.
2. Wubbenhorst, D., et al., *Tetracycline-regulated bone morphogenetic protein 2 gene expression in lentivirally transduced primary rabbit chondrocytes for treatment of cartilage defects.* Arthritis Rheum, 2010. **62**(7): p. 2037-46.
3. Cha, B.H., et al., *Cartilage tissue formation from dedifferentiated chondrocytes by codelivery of BMP-2 and SOX-9 genes encoding bicistronic vector.* Cell Transplant, 2013. **22**(9): p. 1519-28.
4. Zhang, X., et al., *The synergistic effects of microfracture, perforated decalcified cortical bone matrix and adenovirus-bone morphogenetic protein-4 in cartilage defect repair.* Biomaterials, 2008. **29**(35): p. 4616-29.
5. Shi, J., et al., *Nanoparticle delivery of the bone morphogenetic protein 4 gene to adipose-derived stem cells promotes articular cartilage repair in vitro and in vivo.* Arthroscopy, 2013. **29**(12): p. 2001-2011 e2.
6. Matsumoto, T., et al., *Cartilage repair in a rat model of osteoarthritis through intraarticular transplantation of muscle-derived stem cells expressing bone morphogenetic protein 4 and soluble Flt-1.* Arthritis Rheum, 2009. **60**(5): p. 1390-405.
7. Mifune, Y., et al., *The effect of platelet-rich plasma on the regenerative therapy of muscle derived stem cells for articular cartilage repair.* Osteoarthritis Cartilage, 2013. **21**(1): p. 175-85.
8. Sukarto, A., et al., *Co-delivery of adipose-derived stem cells and growth factor-loaded microspheres in RGD-grafted N-methacrylate glycol chitosan gels for focal chondral repair.* Biomacromolecules, 2012. **13**(8): p. 2490-502.
9. Hunter, D.J., et al., *Phase 1 safety and tolerability study of BMP-7 in symptomatic knee osteoarthritis.* BMC Musculoskelet Disord, 2010. **11**: p. 232.
10. Forriol, F., et al., *Meniscal repair possibilities using bone morphogenetic protein-7.* Injury, 2014. **45 Suppl 4**: p. S15-21.
11. Mason, J.M., et al., *Cartilage and bone regeneration using gene-enhanced tissue engineering.* Clin Orthop Relat Res, 2000(379 Suppl): p. S171-8.
12. Yoon, B.H., et al., *An Activin A/BMP2 Chimera Displays Bone Healing Properties Superior to Those of BMP2.* J Bone Miner Res, 2014.
13. Ulrich-Vinther, M., *Gene therapy methods in bone and joint disorders. Evaluation of the adeno-associated virus vector in experimental models of articular cartilage disorders, periprosthetic osteolysis and bone healing.* Acta orthopaedica. Supplementum, 2007. **78**(325): p. 1-64.
14. Itaka, K., et al., *Bone regeneration by regulated in vivo gene transfer using biocompatible polyplex nanomicelles.* Molecular therapy : the journal of the American Society of Gene Therapy, 2007. **15**(9): p. 1655-62.
15. Kleinschmidt, K., et al., *Enhanced reconstruction of long bone architecture by a growth factor mutant combining positive features of GDF-5 and BMP-2.* Biomaterials, 2013. **34**(24): p. 5926-36.
16. Wink, J.D., et al., *Sustained delivery of rhBMP-2 via PLGA microspheres: cranial bone regeneration without heterotopic ossification or craniosynostosis.* Plastic and reconstructive surgery, 2014.
17. Huri, P.Y., et al., *A biomimetic growth factor delivery strategy for enhanced regeneration of iliac crest defects.* Biomedical materials, 2013. **8**(4): p. 045009.
18. Herford, A.S., et al., *Immediate distraction osteogenesis: the sandwich technique in combination with rhBMP-2 for anterior maxillary and mandibular defects.* The Journal of craniofacial surgery, 2013. **24**(4): p. 1383-7.
19. Sonnet, C., et al., *Rapid healing of femoral defects in rats with low dose sustained BMP2 expression from PEGDA hydrogel microspheres.* Journal of orthopaedic research : official publication of the Orthopaedic Research Society, 2013. **31**(10): p. 1597-604.
20. Rodriguez-Evora, M., et al., *Osteogenic effect of local, long versus short term BMP-2 delivery from a novel SPU-PLGA-betaTCP concentric system in a critical size defect in rats.* European journal of pharmaceutical sciences : official journal of the European Federation for Pharmaceutical Sciences, 2013. **49**(5): p. 873-84.
21. Kang, E.J., et al., *Evaluation of the osteogenic activity of the BMP-2 mimetic peptide, PEP7, in vitro and in vivo.* The International journal of oral & maxillofacial implants, 2013. **28**(3): p. 749-56.

22. Zhang, J., et al., *RhBMP-2-loaded calcium silicate/calcium phosphate cement scaffold with hierarchically porous structure for enhanced bone tissue regeneration.* Biomaterials, 2013. **34**(37): p. 9381-92.
23. Fu, Y.C., et al., *Preparation of porous bioceramics using reverse thermo-responsive hydrogels in combination with rhBMP-2 carriers: in vitro and in vivo evaluation.* Journal of the mechanical behavior of biomedical materials, 2013. **27**: p. 64-76.
24. Simank, H.G., et al., *Bone morphogenetic protein-2 and growth and differentiation factor-5 enhance the healing of necrotic bone in a sheep model.* Growth Factors, 2001. **19**(4): p. 247-57.
25. Gruber, R.M., et al., *Mandibular reconstruction using a calcium phosphate/polyethylene glycol hydrogel carrier with BMP-2.* Journal of clinical periodontology, 2014.
26. Shekaran, A., et al., *Bone regeneration using an alpha 2 beta 1 integrin-specific hydrogel as a BMP-2 delivery vehicle.* Biomaterials, 2014. **35**(21): p. 5453-61.
27. Ventura, M., et al., *Monitoring the biological effect of BMP-2 release on bone healing by PET/CT.* Journal of controlled release : official journal of the Controlled Release Society, 2014. **183**: p. 138-44.
28. Qiao, C., et al., *Using poly(lactic-co-glycolic acid) microspheres to encapsulate plasmid of bone morphogenetic protein 2/polyethylenimine nanoparticles to promote bone formation in vitro and in vivo.* International journal of nanomedicine, 2013. **8**: p. 2985-95.
29. Han, C.M., et al., *Creation of nanoporous TiO2 surface onto polyetheretherketone for effective immobilization and delivery of bone morphogenetic protein.* Journal of biomedical materials research. Part A, 2014. **102**(3): p. 793-800.
30. Lee, J.K., et al., *Bone formation and remodeling of three different dental implant surfaces with Escherichia coli-derived recombinant human bone morphogenetic protein 2 in a rabbit model.* The International journal of oral & maxillofacial implants, 2013. **28**(2): p. 424-30.
31. Schwarz, C., et al., *Mechanical load modulates the stimulatory effect of BMP2 in a rat nonunion model.* Tissue engineering. Part A, 2013. **19**(1-2): p. 247-54.
32. Chappuis, V., et al., *Periosteal BMP2 activity drives bone graft healing.* Bone, 2012. **51**(4): p. 800-9.
33. Kim, B.J., et al., *A comparative study of the effectiveness of sinus bone grafting with recombinant human bone morphogenetic protein 2-coated tricalcium phosphate and platelet-rich fibrin-mixed tricalcium phosphate in rabbits.* Oral surgery, oral medicine, oral pathology and oral radiology, 2012. **113**(5): p. 583-92.
34. Diab, T., et al., *A silk hydrogel-based delivery system of bone morphogenetic protein for the treatment of large bone defects.* Journal of the mechanical behavior of biomedical materials, 2012. **11**: p. 123-31.
35. Dumas, J.E., et al., *Injectable reactive biocomposites for bone healing in critical-size rabbit calvarial defects.* Biomedical materials, 2012. **7**(2): p. 024112.
36. Angle, S.R., et al., *Healing of rat femoral segmental defect with bone morphogenetic protein-2: a dose response study.* Journal of musculoskeletal & neuronal interactions, 2012. **12**(1): p. 28-37.
37. Li, D., et al., *Enhancement of osteogenesis by poly(lactide-co-glycolide) sponges loaded with surface-embedded hydroxyapatite particles and rhBMP-2.* Journal of biomedical materials research. Part B, Applied biomaterials, 2012. **100**(4): p. 1103-13.
38. Yoo, J.J., et al., *Synthetic peptide-conjugated titanium alloy for enhanced bone formation in vivo.* Connective tissue research, 2012. **53**(5): p. 359-65.
39. Tan, R., et al., *Repair of rat calvarial bone defects by controlled release of rhBMP-2 from an injectable bone regeneration composite.* Journal of tissue engineering and regenerative medicine, 2012. **6**(8): p. 614-21.
40. Yang, H.S., et al., *Efficient Bone Regeneration Induced by Bone Morphogenetic Protein-2 Released from Apatite-Coated Collagen Scaffolds.* Journal of biomaterials science. Polymer edition, 2011.
41. Zhao, J., et al., *Enhanced healing of rat calvarial defects with sulfated chitosan-coated calcium-deficient hydroxyapatite/bone morphogenetic protein 2 scaffolds.* Tissue engineering. Part A, 2012. **18**(1-2): p. 185-97.
42. Lesaichot, V., et al., *The influence of Bone Morphogenic Protein-2 on the consolidation phase in a distraction osteogenesis model.* Injury, 2011. **42**(12): p. 1460-6.
43. Fujita, N., et al., *An analysis of bone regeneration at a segmental bone defect by controlled release of bone morphogenetic protein 2 from a biodegradable sponge composed of gelatin and beta-tricalcium phosphate.* Journal of tissue engineering and regenerative medicine, 2012. **6**(4): p. 291-8.
44. Kolambkar, Y.M., et al., *Spatiotemporal delivery of bone morphogenetic protein enhances functional repair of segmental bone defects.* Bone, 2011. **49**(3): p. 485-92.

45. Spiro, A.S., et al., *Combined treatment of congenital pseudarthrosis of the tibia, including recombinant human bone morphogenetic protein-2: a case series.* The Journal of bone and joint surgery. British volume, 2011. **93**(5): p. 695-9.
46. Song, D.S., et al., *Enhanced adipogenic differentiation and reduced collagen synthesis induced by human periodontal ligament stem cells might underlie the negative effect of recombinant human bone morphogenetic protein-2 on periodontal regeneration.* Journal of periodontal research, 2011. **46**(2): p. 193-203.
47. Minear, S., et al., *rBMP represses Wnt signaling and influences skeletal progenitor cell fate specification during bone repair.* Journal of bone and mineral research : the official journal of the American Society for Bone and Mineral Research, 2010. **25**(6): p. 1196-207.
48. Cooper, G.M., et al., *Inkjet-based biopatterning of bone morphogenetic protein-2 to spatially control calvarial bone formation.* Tissue engineering. Part A, 2010. **16**(5): p. 1749-59.
49. Nguyen, P.D., et al., *Scaffold-based rhBMP-2 therapy in a rat alveolar defect model: implications for human gingivoperiosteoplasty.* Plastic and reconstructive surgery, 2009. **124**(6): p. 1829-39.
50. Sailhan, F., et al., *Rh-BMP-2 in distraction osteogenesis: dose effect and premature consolidation.* Injury, 2010. **41**(7): p. 680-6.
51. Smeets, R., et al., *Impact of rhBMP-2 on regeneration of buccal alveolar defects during the osseointegration of transgingival inserted implants.* Oral surgery, oral medicine, oral pathology, oral radiology, and endodontics, 2009. **108**(4): p. e3-e12.
52. Boraiah, S., et al., *Complications of recombinant human BMP-2 for treating complex tibial plateau fractures: a preliminary report.* Clinical orthopaedics and related research, 2009. **467**(12): p. 3257-62.
53. Liu, Y., et al., *Segmental bone regeneration using an rhBMP-2-loaded gelatin/nanohydroxyapatite/fibrin scaffold in a rabbit model.* Biomaterials, 2009. **30**(31): p. 6276-85.
54. Sarban, S., et al., *Can rhBMP-2 containing collagen sponges enhance bone repair in ovariectomized rats?: a preliminary study.* Clinical orthopaedics and related research, 2009. **467**(12): p. 3113-20.
55. Azad, V., et al., *rhBMP-2 enhances the bone healing response in a diabetic rat segmental defect model.* Journal of orthopaedic trauma, 2009. **23**(4): p. 267-76.
56. Schnettler, R., et al., *Enhancement of bone formation in hydroxyapatite implants by rhBMP-2 coating.* Journal of biomedical materials research. Part B, Applied biomaterials, 2009. **90**(1): p. 75-81.
57. Wikesjo, U.M., et al., *Bone formation at recombinant human bone morphogenetic protein-2-coated titanium implants in the posterior mandible (Type II bone) in dogs.* Journal of clinical periodontology, 2008. **35**(11): p. 985-91.
58. Bodde, E.W., et al., *The kinetic and biological activity of different loaded rhBMP-2 calcium phosphate cement implants in rats.* Journal of biomedical materials research. Part A, 2008. **87**(3): p. 780-91.
59. Kim, S.S., S.J. Gwak, and B.S. Kim, *Orthotopic bone formation by implantation of apatite-coated poly(lactide-co-glycolide)/hydroxyapatite composite particulates and bone morphogenetic protein-2.* Journal of biomedical materials research. Part A, 2008. **87**(1): p. 245-53.
60. Ma, B., et al., *The effect of simvastatin on bone formation and ceramic resorption in a peri-implant defect model.* Acta biomaterialia, 2008. **4**(1): p. 149-55.
61. Kang, P., et al., *Repairing defect and preventing collapse of canine femoral head using titanium implant enhanced by autogenous bone graft and rhBMP-2.* Connective tissue research, 2007. **48**(4): p. 171-9.
62. Chen, F.M., et al., *Periodontal regeneration using novel glycidyl methacrylated dextran (Dex-GMA)/gelatin scaffolds containing microspheres loaded with bone morphogenetic proteins.* Journal of controlled release : official journal of the Controlled Release Society, 2007. **121**(1-2): p. 81-90.
63. Zheng, L.W. and L.K. Cheung, *Effect of recombinant human bone morphogenetic protein-2 on mandibular distraction at different rates in a rabbit model.* Tissue engineering, 2006. **12**(11): p. 3181-8.
64. Takahashi, Y., et al., *Skull bone regeneration in nonhuman primates by controlled release of bone morphogenetic protein-2 from a biodegradable hydrogel.* Tissue engineering, 2007. **13**(2): p. 293-300.
65. Faria, M.L., et al., *Recombinant human bone morphogenetic protein-2 in absorbable collagen sponge enhances bone healing of tibial osteotomies in dogs.* Veterinary surgery : VS, 2007. **36**(2): p. 122-31.
66. Liu, H.W., et al., *Targeted delivery system for juxtacrine signaling growth factor based on rhBMP-2-mediated carrier-protein conjugation.* Bone, 2006. **39**(4): p. 825-36.
67. Cheung, L.K. and L.W. Zheng, *Effect of recombinant human bone morphogenetic protein-2 on mandibular distraction at different rates in an experimental model.* The Journal of craniofacial surgery, 2006. **17**(1): p. 100-8; discussion 109-10.

68. Siebert, C.H., et al., *Ingrowth of osteochondral grafts under the influence of growth factors: 6-month results of an animal study.* Archives of orthopaedic and trauma surgery, 2006. **126**(4): p. 247-52.
69. Bergenholtz, G., et al., *Observations on healing following endodontic surgery in nonhuman primates (Macaca fascicularis): effects of rhBMP-2.* Oral surgery, oral medicine, oral pathology, oral radiology, and endodontics, 2006. **101**(1): p. 116-25.
70. Schliephake, H., et al., *Effect of immobilized bone morphogenic protein 2 coating of titanium implants on peri-implant bone formation.* Clinical oral implants research, 2005. **16**(5): p. 563-9.
71. Wikesjo, U.M., G. Polimeni, and M. Qahash, *Tissue engineering with recombinant human bone morphogenetic protein-2 for alveolar augmentation and oral implant osseointegration: experimental observations and clinical perspectives.* Clinical implant dentistry and related research, 2005. **7**(2): p. 112-9.
72. Rachmiel, A., D. Aizenbud, and M. Peled, *Enhancement of bone formation by bone morphogenetic protein-2 during alveolar distraction: an experimental study in sheep.* Journal of periodontology, 2004. **75**(11): p. 1524-31.
73. Sykaras, N., et al., *Effect of recombinant human bone morphogenetic protein-2 on the osseointegration of dental implants: a biomechanics study.* Clinical oral investigations, 2004. **8**(4): p. 196-205.
74. Kamakura, S., et al., *New scaffold for recombinant human bone morphogenetic protein-2.* Journal of biomedical materials research. Part A, 2004. **71**(2): p. 299-307.
75. Morcuende, J.A., et al., *Effect of chemotherapy on segmental bone healing enhanced by rhBMP-2.* The Iowa orthopaedic journal, 2004. **24**: p. 36-42.
76. Wikesjo, U.M., et al., *rhBMP-2 significantly enhances guided bone regeneration.* Clinical oral implants research, 2004. **15**(2): p. 194-204.
77. Einhorn, T.A., et al., *A single percutaneous injection of recombinant human bone morphogenetic protein-2 accelerates fracture repair.* The Journal of bone and joint surgery. American volume, 2003. **85-A**(8): p. 1425-35.
78. Blumenthal, N.M., et al., *Effect of surgical implantation of recombinant human bone morphogenetic protein-2 in a bioabsorbable collagen sponge or calcium phosphate putty carrier in intrabony periodontal defects in the baboon.* Journal of periodontology, 2002. **73**(12): p. 1494-506.
79. Murakami, N., et al., *Repair of segmental defects in rabbit humeri with titanium fiber mesh cylinders containing recombinant human bone morphogenetic protein-2 (rhBMP-2) and a synthetic polymer.* Journal of biomedical materials research, 2002. **62**(2): p. 169-74.
80. Luppen, C.A., et al., *Recombinant human bone morphogenetic protein-2 enhances osteotomy healing in glucocorticoid-treated rabbits.* Journal of bone and mineral research : the official journal of the American Society for Bone and Mineral Research, 2002. **17**(2): p. 301-10.
81. Sigurdsson, T.J., S. Nguyen, and U.M. Wikesjo, *Alveolar ridge augmentation with rhBMP-2 and bone-to-implant contact in induced bone.* The International journal of periodontics & restorative dentistry, 2001. **21**(5): p. 461-73.
82. Bouxsein, M.L., et al., *Recombinant human bone morphogenetic protein-2 accelerates healing in a rabbit ulnar osteotomy model.* The Journal of bone and joint surgery. American volume, 2001. **83-A**(8): p. 1219-30.
83. Wikesjo, U.M., R.G. Sorensen, and J.M. Wozney, *Augmentation of alveolar bone and dental implant osseointegration: clinical implications of studies with rhBMP-2.* The Journal of bone and joint surgery. American volume, 2001. **83-A Suppl 1**(Pt 2): p. S136-45.
84. Sebald, H.J., et al., *Inhibition of endogenous antagonists with an engineered BMP-2 variant increases BMP-2 efficacy in rat femoral defect healing.* Acta biomaterialia, 2012. **8**(10): p. 3816-20.
85. Luvizuto, E.R., et al., *The effect of BMP-2 on the osteoconductive properties of beta-tricalcium phosphate in rat calvaria defects.* Biomaterials, 2011. **32**(15): p. 3855-61.
86. Yu, Y.Y., et al., *Bone morphogenetic protein 2 stimulates endochondral ossification by regulating periosteal cell fate during bone repair.* Bone, 2010. **47**(1): p. 65-73.
87. Issa, J.P., et al., *Effect of recombinant human bone morphogenetic protein-2 on bone formation in the acute distraction osteogenesis of rat mandibles.* Clinical oral implants research, 2009. **20**(11): p. 1286-92.
88. Lopez-Lacomba, J.L., et al., *Use of rhBMP-2 activated chitosan films to improve osseointegration.* Biomacromolecules, 2006. **7**(3): p. 792-8.

89. Sachse, A., et al., *Osteointegration of hydroxyapatite-titanium implants coated with nonglycosylated recombinant human bone morphogenetic protein-2 (BMP-2) in aged sheep.* Bone, 2005. **37**(5): p. 699-710.
90. Jung, R.E., et al., *Effect of rhBMP-2 on guided bone regeneration in humans.* Clinical oral implants research, 2003. **14**(5): p. 556-68.
91. Wikesjo, U.M., et al., *Periodontal repair in dogs: space-providing ePTFE devices increase rhBMP-2/ACS-induced bone formation.* Journal of clinical periodontology, 2003. **30**(8): p. 715-25.
92. Yoshida, K., et al., *Enhancement by recombinant human bone morphogenetic protein-2 of bone formation by means of porous hydroxyapatite in mandibular bone defects.* Journal of dental research, 1999. **78**(9): p. 1505-10.
93. Lee, S.C., et al., *Healing of large segmental defects in rat femurs is aided by RhBMP-2 in PLGA matrix.* Journal of biomedical materials research, 1994. **28**(10): p. 1149-56.
94. Hanseler, P., et al., *Delivery of BMP-2 by two clinically available apatite materials: In vitro and in vivo comparison.* Journal of biomedical materials research. Part A, 2014.
95. Lee, J.S., et al., *Controlled release of BMP-2 using a heparin-conjugated carrier system reduces in vivo adipose tissue formation.* Journal of biomedical materials research. Part A, 2014.
96. Pelaez, M., et al., *Effect of rhBMP-2 Dose on Bone Formation/Maturation in a Rat Critical-size Calvarial Defect Model.* Journal of clinical periodontology, 2014.
97. Song, S.H., et al., *Bone Formation in a Rat Tibial Defect Model Using Carboxymethyl Cellulose/BioC/Bone Morphogenic Protein-2 Hybrid Materials.* BioMed research international, 2014. **2014**: p. 230152.
98. Rodriguez-Evora, M., et al., *Bone regeneration induced by an in situ gel-forming poloxamine, bone morphogenetic protein-2 system.* Journal of biomedical nanotechnology, 2014. **10**(6): p. 959-69.
99. Jensen, O.T., et al., *Experience with bone morphogenetic protein-2 and interpositional grafting of edentulous maxillae: a comparison of Le Fort I downfracture to full-arch (horseshoe) segmental osteotomy done in conjunction with sinus floor grafting.* The International journal of oral & maxillofacial implants, 2013. **28**(6): p. e331-48.
100. Casap, N., et al., *Recombinant human bone morphogenetic protein-2 confined by an imperforate titanium shell over high-profile dental implants in rabbit tibiae: a pilot bone augmentation study.* The International journal of oral & maxillofacial implants, 2013. **28**(6): p. e349-56.
101. Marx, R.E., et al., *rhBMP-2/ACS grafts versus autogenous cancellous marrow grafts in large vertical defects of the maxilla: an unsponsored randomized open-label clinical trial.* The International journal of oral & maxillofacial implants, 2013. **28**(5): p. e243-51.
102. Cottam, J.R., et al., *Closure of 1.5-cm alveolar oral antral fistula with intra-alveolar sinus membrane elevation and bone morphogenetic protein-2/collagen graft followed by dental implant restoration: case report.* The International journal of oral & maxillofacial implants, 2013. **28**(5): p. e277-82.
103. Cha, J.K., et al., *Sinus augmentation using BMP-2 in a bovine hydroxyapatite/collagen carrier in dogs.* Journal of clinical periodontology, 2014. **41**(1): p. 86-93.
104. Kim, J.S., et al., *Acceleration of Bone Regeneration by BMP-2-Loaded Collagenated Biphasic Calcium Phosphate in Rabbit Sinus.* Clinical implant dentistry and related research, 2014.
105. Lai, C.H., et al., *Use of a collagen membrane loaded with recombinant human bone morphogenetic protein-2 with collagen-binding domain for vertical guided bone regeneration.* Journal of periodontology, 2013. **84**(7): p. 950-7.
106. Lopes, N.M., et al., *Use of rhBMP-2 to reconstruct a severely atrophic mandible: a modified approach.* International journal of oral and maxillofacial surgery, 2012. **41**(12): p. 1566-70.
107. Yamashita, M., et al., *A pilot experimental lateral ridge augmentation study using bone morphogenetic protein 2 in dogs.* The International journal of periodontics & restorative dentistry, 2010. **30**(5): p. 457-69.
108. Thoma, D.S., et al., *Ridge augmentation using recombinant bone morphogenetic protein-2 techniques: an experimental study in the canine.* Journal of periodontology, 2010. **81**(12): p. 1829-38.
109. O'Shaughnessy, B.A., T.R. Kuklo, and S.L. Ondra, *Surgical treatment of vertebral osteomyelitis with recombinant human bone morphogenetic protein-2.* Spine (Phila Pa 1976), 2008. **33**(5): p. E132-9.
110. Strassmair, M., et al., *The use of a Type-I lyophilisate collagen as an osteoinductive factor in pseudarthroses of the forearm.* Surg Technol Int, 2009. **18**: p. 213-8.

111. Lyon, T., et al., *Efficacy and safety of recombinant human bone morphogenetic protein-2/calcium phosphate matrix for closed tibial diaphyseal fracture: a double-blind, randomized, controlled phase-II/III trial.* J Bone Joint Surg Am, 2013. **95**(23): p. 2088-96.
112. Flierl, M.A., et al., *Outcomes and complication rates of different bone grafting modalities in long bone fracture nonunions: a retrospective cohort study in 182 patients.* J Orthop Surg Res, 2013. **8**: p. 33.
113. Skogh, A.C., et al., *Variation in calvarial bone healing capacity: a clinical study on the effects of BMP-2-hydrogel or bone autograft treatments at different cranial locations.* J Craniofac Surg, 2013. **24**(2): p. 339-43.
114. Canan, L.W., Jr., et al., *Human bone morphogenetic protein-2 use for maxillary reconstruction in cleft lip and palate patients.* J Craniofac Surg, 2012. **23**(6): p. 1627-33.
115. Neovius, E., et al., *Alveolar bone healing accompanied by severe swelling in cleft children treated with bone morphogenetic protein-2 delivered by hydrogel.* J Plast Reconstr Aesthet Surg, 2013. **66**(1): p. 37-42.
116. Tsuzuki, N., et al., *In vivo osteoinductivity of gelatin beta-tri-calcium phosphate sponge and bone morphogenetic protein-2 on an equine third metacarpal bone defect.* Res Vet Sci, 2012. **93**(2): p. 1021-5.
117. Aro, H.T., et al., *Recombinant human bone morphogenetic protein-2: a randomized trial in open tibial fractures treated with reamed nail fixation.* J Bone Joint Surg Am, 2011. **93**(9): p. 801-8.
118. Alonso, N., et al., *Evaluation of maxillary alveolar reconstruction using a resorbable collagen sponge with recombinant human bone morphogenetic protein-2 in cleft lip and palate patients.* Tissue Eng Part C Methods, 2010. **16**(5): p. 1183-9.
119. Alt, V., et al., *A health economic analysis of the use of rhBMP-2 in Gustilo-Anderson grade III open tibial fractures for the UK, Germany, and France.* Injury, 2009. **40**(12): p. 1269-75.
120. Jung, R.E., et al., *A randomized-controlled clinical trial evaluating clinical and radiological outcomes after 3 and 5 years of dental implants placed in bone regenerated by means of GBR techniques with or without the addition of BMP-2.* Clin Oral Implants Res, 2009. **20**(7): p. 660-6.
121. Dickinson, B.P., et al., *Reduced morbidity and improved healing with bone morphogenic protein-2 in older patients with alveolar cleft defects.* Plast Reconstr Surg, 2008. **121**(1): p. 209-17.
122. Jones, A.L., et al., *Recombinant human BMP-2 and allograft compared with autogenous bone graft for reconstruction of diaphyseal tibial fractures with cortical defects. A randomized, controlled trial.* J Bone Joint Surg Am, 2006. **88**(7): p. 1431-41.
123. Swiontkowski, M.F., et al., *Recombinant human bone morphogenetic protein-2 in open tibial fractures. A subgroup analysis of data combined from two prospective randomized studies.* J Bone Joint Surg Am, 2006. **88**(6): p. 1258-65.
124. Bianchi, J., et al., *Measuring the efficacy of rhBMP-2 to regenerate bone: a radiographic study using a commercially available software program.* Int J Periodontics Restorative Dent, 2004. **24**(6): p. 579-87.
125. Govender, S., et al., *Recombinant human bone morphogenetic protein-2 for treatment of open tibial fractures: a prospective, controlled, randomized study of four hundred and fifty patients.* J Bone Joint Surg Am, 2002. **84-A**(12): p. 2123-34.
126. Maniscalco, P., et al., *Healing of fresh tibial fractures with OP-1. A preliminary report.* Acta Biomed, 2002. **73**(1-2): p. 27-33.
127. Riedel, G.E. and A. Valentin-Opran, *Clinical evaluation of rhBMP-2/ACS in orthopedic trauma: a progress report.* Orthopedics, 1999. **22**(7): p. 663-5.
128. de Freitas, R.M., et al., *Horizontal ridge augmentation of the atrophic anterior maxilla using rhBMP-2/ACS or autogenous bone grafts: a proof-of-concept randomized clinical trial.* J Clin Periodontol, 2013. **40**(10): p. 968-75.
129. Kao, D.W., et al., *The negative effect of combining rhBMP-2 and Bio-Oss on bone formation for maxillary sinus augmentation.* Int J Periodontics Restorative Dent, 2012. **32**(1): p. 61-7.
130. Triplett, R.G., et al., *Pivotal, randomized, parallel evaluation of recombinant human bone morphogenetic protein-2/absorbable collagen sponge and autogenous bone graft for maxillary sinus floor augmentation.* J Oral Maxillofac Surg, 2009. **67**(9): p. 1947-60.
131. Serra, E.S.F.M., J. Ricardo de Albergaria-Barbosa, and R. Mazzonetto, *Clinical evaluation of association of bovine organic osseous matrix and bovine bone morphogenetic protein versus autogenous bone graft in sinus floor augmentation.* J Oral Maxillofac Surg, 2006. **64**(6): p. 931-5.
132. Boyne, P.J., et al., *De novo bone induction by recombinant human bone morphogenetic protein-2 (rhBMP-2) in maxillary sinus floor augmentation.* J Oral Maxillofac Surg, 2005. **63**(12): p. 1693-707.

133. Fiorellini, J.P., et al., *Randomized study evaluating recombinant human bone morphogenetic protein-2 for extraction socket augmentation.* J Periodontol, 2005. **76**(4): p. 605-13.
134. Cochran, D.L., et al., *Evaluation of recombinant human bone morphogenetic protein-2 in oral applications including the use of endosseous implants: 3-year results of a pilot study in humans.* J Periodontol, 2000. **71**(8): p. 1241-57.
135. Boyne, P.J., et al., *A feasibility study evaluating rhBMP-2/absorbable collagen sponge for maxillary sinus floor augmentation.* Int J Periodontics Restorative Dent, 1997. **17**(1): p. 11-25.
136. Howell, T.H., et al., *A feasibility study evaluating rhBMP-2/absorbable collagen sponge device for local alveolar ridge preservation or augmentation.* Int J Periodontics Restorative Dent, 1997. **17**(2): p. 124-39.
137. Kim, H.C., et al., *Combined effect of bisphosphonate and recombinant human bone morphogenetic protein 2 on bone healing of rat calvarial defects.* Maxillofac Plast Reconstr Surg, 2015. **37**(1): p. 16.
138. Chen, X., et al., *Union of a chronically infected internally stabilized segmental defect in the rat femur after debridement and application of rhBMP-2 and systemic antibiotic.* Journal of orthopaedic trauma, 2007. **21**(10): p. 693-700.
139. Sandor, G.K., et al., *Adipose stem cells used to reconstruct 13 cases with cranio-maxillofacial hard-tissue defects.* Stem Cells Transl Med, 2014. **3**(4): p. 530-40.
140. Smith, D.M., et al., *Regenerative surgery in cranioplasty revisited: the role of adipose-derived stem cells and BMP-2.* Plastic and reconstructive surgery, 2011. **128**(5): p. 1053-60.
141. Kang, S.W., et al., *Combination therapy with BMP-2 and BMSCs enhances bone healing efficacy of PCL scaffold fabricated using the 3D plotting system in a large segmental defect model.* Biotechnology letters, 2012. **34**(7): p. 1375-84.
142. Stephan, S.J., et al., *Injectable tissue-engineered bone repair of a rat calvarial defect.* The Laryngoscope, 2010. **120**(5): p. 895-901.
143. Xia, L., et al., *Maxillary sinus floor elevation using a tissue-engineered bone with rhBMP-2-loaded porous calcium phosphate cement scaffold and bone marrow stromal cells in rabbits.* Cells, tissues, organs, 2011. **194**(6): p. 481-93.
144. Jo, S., et al., *Effects of recombinant human bone morphogenic protein-2 and human bone marrow-derived stromal cells on in vivo bone regeneration of chitosan-poly(ethylene oxide) hydrogel.* J Biomed Mater Res A, 2013. **101**(3): p. 892-901.
145. Marx, R.E. and D.B. Harrell, *Translational research: The CD34+ cell is crucial for large-volume bone regeneration from the milieu of bone marrow progenitor cells in craniomandibular reconstruction.* Int J Oral Maxillofac Implants, 2014. **29**(2): p. e201-9.
146. Terella, A., et al., *Repair of a calvarial defect with biofactor and stem cell-embedded polyethylene glycol scaffold.* Archives of facial plastic surgery, 2010. **12**(3): p. 166-71.
147. Kirker-Head, C., et al., *BMP-silk composite matrices heal critically sized femoral defects.* Bone, 2007. **41**(2): p. 247-55.
148. Rodriguez-Evora, M., et al., *Smurf1 Knocked-Down, Mesenchymal Stem Cells and BMP-2 in an Electrospun System for Bone Regeneration.* Biomacromolecules, 2014. **15**(4): p. 1311-22.
149. Sun, J.S., et al., *Vitamin-D binding protein does not enhance healing in rat bone defects: a pilot study.* Clinical orthopaedics and related research, 2009. **467**(12): p. 3156-64.
150. Lee, J.H., et al., *Synergistic induction of early stage of bone formation by combination of recombinant human bone morphogenetic protein-2 and epidermal growth factor.* Journal of tissue engineering and regenerative medicine, 2014.
151. Patel, J.J., et al., *Dual Delivery of EPO and BMP2 from a Novel Modular Poly-varepsilon-Caprolactone Construct to Increase the Bone Formation in Prefabricated Bone Flaps.* Tissue Eng Part C Methods, 2015. **21**(9): p. 889-97.
152. Su, J., et al., *Dual Delivery of BMP-2 and bFGF from a New Nano-Composite Scaffold, Loaded with Vascular Stents for Large-Size Mandibular Defect Regeneration.* International journal of molecular sciences, 2013. **14**(6): p. 12714-28.
153. Springer, I.N., et al., *BMP-2 and bFGF in an irradiated bone model.* Journal of cranio-maxillo-facial surgery : official publication of the European Association for Cranio-Maxillo-Facial Surgery, 2008. **36**(4): p. 210-7.
154. Charles, L.F., et al., *Effects of low dose FGF-2 and BMP-2 on healing of calvarial defects in old mice.* Exp Gerontol, 2015. **64**: p. 62-9.

155. Angle, S.R., et al., *Combined Use of Low Intensity Pulsed Ultrasound and rhBMP-2 to Enhance Bone Formation in a Rat Model of Critical-Size Defect.* J Orthop Trauma, 2014.
156. Bougioukli, S., et al., *Combination therapy with BMP-2 and a systemic RANKL inhibitor enhances bone healing in a mouse critical-sized femoral defect.* Bone, 2015. **84**: p. 93-103.
157. Yang, H.J., R.Y. Kim, and S.J. Hwang, *Pulsed Electromagnetic Fields Enhance Bone Morphogenetic Protein-2 Dependent-Bone Regeneration.* Tissue Eng Part A, 2015. **21**(19-20): p. 2629-37.
158. Park, E.J., et al., *Improved bone healing by angiogenic factor-enriched platelet-rich plasma and its synergistic enhancement by bone morphogenetic protein-2.* The International journal of oral & maxillofacial implants, 2008. **23**(5): p. 818-26.
159. Por, Y.C., et al., *Bone generation in the reconstruction of a critical size calvarial defect in an experimental model.* Annals of the Academy of Medicine, Singapore, 2007. **36**(11): p. 911-9.
160. Kempen, D.H., et al., *Enhanced bone morphogenetic protein-2-induced ectopic and orthotopic bone formation by intermittent parathyroid hormone (1-34) administration.* Tissue engineering. Part A, 2010. **16**(12): p. 3769-77.
161. Fan, J., et al., *Delivery of Phenamil Enhances BMP-2-Induced Osteogenic Differentiation of Adipose-Derived Stem Cells and Bone Formation in Calvarial Defects.* Tissue Eng Part A, 2015. **21**(13-14): p. 2053-65.
162. Holloway, J.L., et al., *Synergistic Effects of SDF-1alpha and BMP-2 Delivery from Proteolytically Degradable Hyaluronic Acid Hydrogels for Bone Repair.* Macromol Biosci, 2015. **15**(9): p. 1218-23.
163. Hwang, H.D., et al., *Sequential Treatment with SDF-1 and BMP-2 Potentiates Bone Formation in Calvarial Defects.* Tissue Eng Part A, 2015. **21**(13-14): p. 2125-35.
164. Lee, C.H., et al., *Effect of dual treatment with SDF-1 and BMP-2 on ectopic and orthotopic bone formation.* PLoS One, 2015. **10**(3): p. e0120051.
165. Thorey, F., et al., *Osseointegration by bone morphogenetic protein-2 and transforming growth factor beta2 coated titanium implants in femora of New Zealand white rabbits.* Indian journal of orthopaedics, 2011. **45**(1): p. 57-62.
166. Ramazanoglu, M., et al., *The effect of combined delivery of recombinant human bone morphogenetic protein-2 and recombinant human vascular endothelial growth factor 165 from biomimetic calcium-phosphate-coated implants on osseointegration.* Clinical oral implants research, 2011. **22**(12): p. 1433-9.
167. Patel, Z.S., et al., *Dual delivery of an angiogenic and an osteogenic growth factor for bone regeneration in a critical size defect model.* Bone, 2008. **43**(5): p. 931-40.
168. Lohse, N., et al., *Continuous delivery of rhBMP2 and rhVEGF165 at a certain ratio enhances bone formation in mandibular defects over the delivery of rhBMP2 alone - An experimental study in rats.* J Control Release, 2015. **220**(Pt A): p. 201-9.
169. Kempen, D.H., et al., *Effect of local sequential VEGF and BMP-2 delivery on ectopic and orthotopic bone regeneration.* Biomaterials, 2009. **30**(14): p. 2816-25.
170. Kroczek, A., et al., *Effects of osteoinduction on bone regeneration in distraction: results of a pilot study.* Journal of cranio-maxillo-facial surgery : official publication of the European Association for Cranio-Maxillo-Facial Surgery, 2010. **38**(5): p. 334-44.
171. Ishihara, A., et al., *Comparative efficacy of dermal fibroblast-mediated and direct adenoviral bone morphogenetic protein-2 gene therapy for bone regeneration in an equine rib model.* Gene therapy, 2010. **17**(6): p. 733-44.
172. Menendez, M.I., et al., *Direct delayed human adenoviral BMP-2 or BMP-6 gene therapy for bone and cartilage regeneration in a pony osteochondral model.* Osteoarthritis and cartilage / OARS, Osteoarthritis Research Society, 2011. **19**(8): p. 1066-75.
173. Alden, T.D., et al., *The use of bone morphogenetic protein gene therapy in craniofacial bone repair.* The Journal of craniofacial surgery, 2000. **11**(1): p. 24-30.
174. Gafni, Y., et al., *Gene therapy platform for bone regeneration using an exogenously regulated, AAV-2-based gene expression system.* Molecular therapy : the journal of the American Society of Gene Therapy, 2004. **9**(4): p. 587-95.
175. Uusitalo, H., et al., *Induction of periosteal callus formation by bone morphogenetic protein-2 employing adenovirus-mediated gene delivery.* Matrix biology : journal of the International Society for Matrix Biology, 2001. **20**(2): p. 123-7.
176. Hu, W.W., et al., *Bone regeneration in defects compromised by radiotherapy.* Journal of dental research, 2010. **89**(1): p. 77-81.

177. Santoni, B.G., et al., *Gene therapy to enhance allograft incorporation after host tissue irradiation.* Clinical orthopaedics and related research, 2008. **466**(8): p. 1921-9.
178. Betz, O.B., et al., *Delayed administration of adenoviral BMP-2 vector improves the formation of bone in osseous defects.* Gene therapy, 2007. **14**(13): p. 1039-44.
179. Hu, W.W., et al., *Localized viral vector delivery to enhance in situ regenerative gene therapy.* Gene therapy, 2007. **14**(11): p. 891-901.
180. Park, J., et al., *The effect on bone regeneration of a liposomal vector to deliver BMP-2 gene to bone grafts in peri-implant bone defects.* Biomaterials, 2007. **28**(17): p. 2772-82.
181. Egermann, M., et al., *Effect of BMP-2 gene transfer on bone healing in sheep.* Gene therapy, 2006. **13**(17): p. 1290-9.
182. Ashinoff, R.L., et al., *Bone morphogenic protein-2 gene therapy for mandibular distraction osteogenesis.* Annals of plastic surgery, 2004. **52**(6): p. 585-90; discussion 591.
183. Ono, I., et al., *Combination of porous hydroxyapatite and cationic liposomes as a vector for BMP-2 gene therapy.* Biomaterials, 2004. **25**(19): p. 4709-18.
184. Uusitalo, H., et al., *Accelerated up-regulation of L-Sox5, Sox6, and Sox9 by BMP-2 gene transfer during murine fracture healing.* Journal of bone and mineral research : the official journal of the American Society for Bone and Mineral Research, 2001. **16**(10): p. 1837-45.
185. Lindsey, W.H., *Osseous tissue engineering with gene therapy for facial bone reconstruction.* The Laryngoscope, 2001. **111**(7): p. 1128-36.
186. Baltzer, A.W., et al., *Potential role of direct adenoviral gene transfer in enhancing fracture repair.* Clinical orthopaedics and related research, 2000(379 Suppl): p. S120-5.
187. Baltzer, A.W., et al., *Genetic enhancement of fracture repair: healing of an experimental segmental defect by adenoviral transfer of the BMP-2 gene.* Gene therapy, 2000. **7**(9): p. 734-9.
188. He, F.M., et al., *Bone formation at porous titanium implants coated with multiple layers of recombinant human bone morphogenetic protein-2 cDNA plasmid in the posterior mandible in dogs.* The International journal of oral & maxillofacial implants, 2013. **28**(6): p. 1648-54.
189. Lin, C.Y., et al., *The use of ASCs engineered to express BMP2 or TGF-beta3 within scaffold constructs to promote calvarial bone repair.* Biomaterials, 2013. **34**(37): p. 9401-12.
190. Betz, O.B., et al., *The repair of critical-sized bone defects using expedited, autologous BMP-2 gene-activated fat implants.* Tissue engineering. Part A, 2010. **16**(3): p. 1093-101.
191. Li, H., et al., *Bone regeneration by implantation of adipose-derived stromal cells expressing BMP-2.* Biochemical and biophysical research communications, 2007. **356**(4): p. 836-42.
192. Peterson, B., et al., *Healing of critically sized femoral defects, using genetically modified mesenchymal stem cells from human adipose tissue.* Tissue engineering, 2005. **11**(1-2): p. 120-9.
193. Liao, Y.H., et al., *Osteogenic differentiation of adipose-derived stem cells and calvarial defect repair using baculovirus-mediated co-expression of BMP-2 and miR-148b.* Biomaterials, 2014. **35**(18): p. 4901-10.
194. Feichtinger, G.A., et al., *Sonoporation increases therapeutic efficacy of inducible and constitutive BMP2/7 in vivo gene delivery.* Human gene therapy methods, 2014. **25**(1): p. 57-71.
195. Koh, J.T., et al., *Combinatorial gene therapy with BMP2/7 enhances cranial bone regeneration.* Journal of dental research, 2008. **87**(9): p. 845-9.
196. Tang, Y., et al., *Combination of bone tissue engineering and BMP-2 gene transfection promotes bone healing in osteoporotic rats.* Cell biology international, 2008. **32**(9): p. 1150-7.
197. Feeley, B.T., et al., *In vivo molecular imaging of adenoviral versus lentiviral gene therapy in two bone formation models.* Journal of orthopaedic research : official publication of the Orthopaedic Research Society, 2006. **24**(8): p. 1709-21.
198. Turgeman, G., et al., *Engineered human mesenchymal stem cells: a novel platform for skeletal cell mediated gene therapy.* The journal of gene medicine, 2001. **3**(3): p. 240-51.
199. Lieberman, J.R., et al., *Regional gene therapy with a BMP-2-producing murine stromal cell line induces heterotopic and orthotopic bone formation in rodents.* Journal of orthopaedic research : official publication of the Orthopaedic Research Society, 1998. **16**(3): p. 330-9.
200. Castro-Govea, Y., et al., *Human bone morphogenetic protein 2-transduced mesenchymal stem cells improve bone regeneration in a model of mandible distraction surgery.* The Journal of craniofacial surgery, 2012. **23**(2): p. 392-6.

201. Park, S.Y., et al., *The evaluation of the correlation between histomorphometric analysis and micro-computed tomography analysis in AdBMP-2 induced bone regeneration in rat calvarial defects.* Journal of periodontal & implant science, 2011. **41**(5): p. 218-26.
202. Dupont, K.M., et al., *Synthetic scaffold coating with adeno-associated virus encoding BMP2 to promote endogenous bone repair.* Cell and tissue research, 2012. **347**(3): p. 575-88.
203. Zhu, C., et al., *LvBMP-2 gene-modified BMSCs combined with calcium phosphate cement scaffolds for the repair of calvarial defects in rats.* Journal of materials science. Materials in medicine, 2011. **22**(8): p. 1965-73.
204. Virk, M.S., et al., *"Same day" ex-vivo regional gene therapy: a novel strategy to enhance bone repair.* Molecular therapy : the journal of the American Society of Gene Therapy, 2011. **19**(5): p. 960-8.
205. Long, J., et al., *Effects of bone morphogenetic protein 2 gene therapy on new bone formation during mandibular distraction osteogenesis at rapid rate in rabbits.* Oral surgery, oral medicine, oral pathology, oral radiology, and endodontics, 2011. **112**(1): p. 50-7.
206. Kallai, I., et al., *Quantitative, structural, and image-based mechanical analysis of nonunion fracture repaired by genetically engineered mesenchymal stem cells.* Journal of biomechanics, 2010. **43**(12): p. 2315-20.
207. Chang, S.C., et al., *Large-scale bicortical skull bone regeneration using ex vivo replication-defective adenoviral-mediated bone morphogenetic protein-2 gene-transferred bone marrow stromal cells and composite biomaterials.* Neurosurgery, 2009. **65**(6 Suppl): p. 75-81; discussion 81-3.
208. Zhao, J., et al., *Combination of beta-TCP and BMP-2 gene-modified bMSCs to heal critical size mandibular defects in rats.* Oral diseases, 2010. **16**(1): p. 46-54.
209. Yan, M.N., et al., *Reconstruction of peri-implant bone defects using impacted bone allograft and BMP-2 gene-modified bone marrow stromal cells.* Journal of biomedical materials research. Part A, 2010. **93**(1): p. 304-13.
210. Jiang, X., et al., *Mandibular repair in rats with premineralized silk scaffolds and BMP-2-modified bMSCs.* Biomaterials, 2009. **30**(27): p. 4522-32.
211. Chen, Y.L., et al., *Periodontal regeneration using ex vivo autologous stem cells engineered to express the BMP-2 gene: an alternative to alveolaplasty.* Gene therapy, 2008. **15**(22): p. 1469-77.
212. Steinhardt, Y., et al., *Maxillofacial-derived stem cells regenerate critical mandibular bone defect.* Tissue engineering. Part A, 2008. **14**(11): p. 1763-73.
213. Tai, K., et al., *Nanobiomechanics of repair bone regenerated by genetically modified mesenchymal stem cells.* Tissue engineering. Part A, 2008. **14**(10): p. 1709-20.
214. Virk, M.S., et al., *Influence of short-term adenoviral vector and prolonged lentiviral vector mediated bone morphogenetic protein-2 expression on the quality of bone repair in a rat femoral defect model.* Bone, 2008. **42**(5): p. 921-31.
215. Yue, B., et al., *BMP2 gene therapy on the repair of bone defects of aged rats.* Calcified tissue international, 2005. **77**(6): p. 395-403.
216. Zhang, X., et al., *Periosteal progenitor cell fate in segmental cortical bone graft transplantations: implications for functional tissue engineering.* Journal of bone and mineral research : the official journal of the American Society for Bone and Mineral Research, 2005. **20**(12): p. 2124-37.
217. Dai, K.R., et al., *Repairing of goat tibial bone defects with BMP-2 gene-modified tissue-engineered bone.* Calcified tissue international, 2005. **77**(1): p. 55-61.
218. Blum, J.S., et al., *In vivo evaluation of gene therapy vectors in ex vivo-derived marrow stromal cells for bone regeneration in a rat critical-size calvarial defect model.* Human gene therapy, 2003. **14**(18): p. 1689-701.
219. Chang, S.C., et al., *Ex vivo gene therapy in autologous critical-size craniofacial bone regeneration.* Plastic and reconstructive surgery, 2003. **112**(7): p. 1841-50.
220. Chang, S.C., et al., *Ex vivo gene therapy in autologous bone marrow stromal stem cells for tissue-engineered maxillofacial bone regeneration.* Gene therapy, 2003. **10**(24): p. 2013-9.
221. Park, J., et al., *Bone regeneration in critical size defects by cell-mediated BMP-2 gene transfer: a comparison of adenoviral vectors and liposomes.* Gene therapy, 2003. **10**(13): p. 1089-98.
222. Moutsatsos, I.K., et al., *Exogenously regulated stem cell-mediated gene therapy for bone regeneration.* Molecular therapy : the journal of the American Society of Gene Therapy, 2001. **3**(4): p. 449-61.
223. Gazit, D., et al., *Engineered pluripotent mesenchymal cells integrate and differentiate in regenerating bone: a novel cell-mediated gene therapy.* The journal of gene medicine, 1999. **1**(2): p. 121-33.

224. Lieberman, J.R., et al., *The effect of regional gene therapy with bone morphogenetic protein-2-producing bone-marrow cells on the repair of segmental femoral defects in rats.* The Journal of bone and joint surgery. American volume, 1999. **81**(7): p. 905-17.
225. Alaee, F., et al., *Suicide gene approach using a dual-expression lentiviral vector to enhance the safety of ex vivo gene therapy for bone repair.* Gene therapy, 2014. **21**(2): p. 139-47.
226. Jhin, M.J., et al., *Ex vivo bone morphogenetic protein-2 gene delivery using bone marrow stem cells in rabbit maxillary sinus augmentation in conjunction with implant placement.* Journal of periodontology, 2013. **84**(7): p. 985-94.
227. Jiang, X.Q., et al., *Maxillary sinus floor elevation using a tissue-engineered bone complex with beta-TCP and BMP-2 gene-modified bMSCs in rabbits.* Clin Oral Implants Res, 2009. **20**(12): p. 1333-40.
228. Park, S.Y., et al., *Dual delivery of rhPDGF-BB and bone marrow mesenchymal stromal cells expressing the BMP2 gene enhance bone formation in a critical-sized defect model.* Tissue engineering. Part A, 2013. **19**(21-22): p. 2495-505.
229. He, X., et al., *BMP2 genetically engineered MSCs and EPCs promote vascularized bone regeneration in rat critical-sized calvarial bone defects.* PloS one, 2013. **8**(4): p. e60473.
230. Wang, R., et al., *Autografts and xenografts of skin fibroblasts delivering BMP-2 effectively promote orthotopic and ectopic osteogenesis.* Anatomical record, 2009. **292**(6): p. 777-86.
231. Lee, J.Y., et al., *Enhancement of bone healing based on ex vivo gene therapy using human muscle-derived cells expressing bone morphogenetic protein 2.* Human gene therapy, 2002. **13**(10): p. 1201-11.
232. Lee, J.Y., et al., *Effect of bone morphogenetic protein-2-expressing muscle-derived cells on healing of critical-sized bone defects in mice.* The Journal of bone and joint surgery. American volume, 2001. **83-A**(7): p. 1032-9.
233. Day, C.S., et al., *Use of muscle cells to mediate gene transfer to the bone defect.* Tissue engineering, 1999. **5**(2): p. 119-25.
234. Park, S.Y., et al., *Ex vivo bone morphogenetic protein 2 gene delivery using periodontal ligament stem cells for enhanced re-osseointegration in the regenerative treatment of peri-implantitis.* J Biomed Mater Res A, 2014.
235. Wu, G., et al., *Effect of gene transfecting at different times on mandibular distraction osteogenesis.* The Journal of craniofacial surgery, 2013. **24**(1): p. 232-6.
236. Lin, X., et al., *Augmentation of osseous phenotypes in vivo with a synthetic peptide.* J Orthop Res, 2007. **25**(4): p. 531-9.
237. Ferreira, C.L., et al., *TGF-beta1 and BMP-4 carried by liposomes enhance the healing process in alveolar bone.* Archives of oral biology, 2013. **58**(6): p. 646-56.
238. Neudert, M., et al., *Osseointegration of titanium prostheses on the stapes footplate.* Journal of the Association for Research in Otolaryngology : JARO, 2010. **11**(2): p. 161-71.
239. Jung, U.W., et al., *The effect of varying the particle size of beta tricalcium phosphate carrier of recombinant human bone morphogenetic protein-4 on bone formation in rat calvarial defects.* Journal of periodontology, 2006. **77**(5): p. 765-72.
240. Ahn, S.H., et al., *Effect of recombinant human bone morphogenetic protein-4 with carriers in rat calvarial defects.* Journal of periodontology, 2003. **74**(6): p. 787-97.
241. Chen, J.C., et al., *rhBMP-4 gene therapy in a juvenile canine alveolar defect model.* Plastic and reconstructive surgery, 2007. **120**(6): p. 1503-9.
242. Huang, Y.C., et al., *Bone regeneration in a rat cranial defect with delivery of PEI-condensed plasmid DNA encoding for bone morphogenetic protein-4 (BMP-4).* Gene therapy, 2005. **12**(5): p. 418-26.
243. Rundle, C.H., et al., *In vivo bone formation in fracture repair induced by direct retroviral-based gene therapy with bone morphogenetic protein-4.* Bone, 2003. **32**(6): p. 591-601.
244. Jiang, X., et al., *The use of tissue-engineered bone with human bone morphogenetic protein-4-modified bone-marrow stromal cells in repairing mandibular defects in rabbits.* International journal of oral and maxillofacial surgery, 2006. **35**(12): p. 1133-9.
245. Rose, T., et al., *The role of cell type in bone healing mediated by ex vivo gene therapy.* Langenbeck's archives of surgery / Deutsche Gesellschaft fur Chirurgie, 2003. **388**(5): p. 347-55.
246. Gysin, R., et al., *Ex vivo gene therapy with stromal cells transduced with a retroviral vector containing the BMP4 gene completely heals critical size calvarial defect in rats.* Gene therapy, 2002. **9**(15): p. 991-9.

247. Rose, T., et al., *Gene therapy to improve osteogenesis in bone lesions with severe soft tissue damage.* Langenbeck's archives of surgery / Deutsche Gesellschaft fur Chirurgie, 2003. **388**(5): p. 356-65.
248. Song, K., et al., *Identification of a key residue mediating bone morphogenetic protein (BMP)-6 resistance to noggin inhibition allows for engineered BMPs with superior agonist activity.* The Journal of biological chemistry, 2010. **285**(16): p. 12169-80.
249. Papanagiotou, M., et al., *Autologous (non-vascularised) fibular grafting with recombinant bone morphogenetic protein-7 for the treatment of femoral head osteonecrosis: preliminary report.* The bone & joint journal, 2014. **96-B**(1): p. 31-5.
250. Cloutier, M., et al., *Calvarial bone wound healing: a comparison between carbide and diamond drills, Er:YAG and Femtosecond lasers with or without BMP-7.* Oral surgery, oral medicine, oral pathology, oral radiology, and endodontics, 2010. **110**(6): p. 720-8.
251. Draenert, M.E., et al., *Primary cancellous bone formation with BMP and micro-chambered beads: experimental study on sheep.* Bone, 2013. **52**(1): p. 465-73.
252. Saran, N., R. Zhang, and R.E. Turcotte, *Osteogenic protein-1 delivered by hydroxyapatite-coated implants improves bone ingrowth in extracortical bone bridging.* Clinical orthopaedics and related research, 2011. **469**(5): p. 1470-8.
253. Gilley, R.S., et al., *OP-1 augments glucocorticoid-inhibited fracture healing in a rat fracture model.* Clinical orthopaedics and related research, 2009. **467**(12): p. 3104-12.
254. Kidder, L.S., et al., *Osteogenic protein-1 overcomes inhibition of fracture healing in the diabetic rat: a pilot study.* Clinical orthopaedics and related research, 2009. **467**(12): p. 3249-56.
255. Zhou, A.J., S.A. Peel, and C.M. Clokie, *An evaluation of hydroxyapatite and biphasic calcium phosphate in combination with Pluronic F127 and BMP on bone repair.* The Journal of craniofacial surgery, 2007. **18**(6): p. 1264-75.
256. Ristiniemi, J., et al., *RhBMP-7 accelerates the healing in distal tibial fractures treated by external fixation.* The Journal of bone and joint surgery. British volume, 2007. **89**(2): p. 265-72.
257. Jensen, T.B., et al., *Osteogenic protein-1 increases the fixation of implants grafted with morcellised bone allograft and ProOsteon bone substitute: an experimental study in dogs.* The Journal of bone and joint surgery. British volume, 2007. **89**(1): p. 121-6.
258. Mandu-Hrit, M., et al., *Early injection of OP-1 during distraction osteogenesis accelerates new bone formation in rabbits.* Growth Factors, 2006. **24**(3): p. 172-83.
259. Zakhary, K., et al., *Effect of recombinant human bone morphogenetic protein 7 on bone density during distraction osteogenesis of the rabbit mandible.* The Journal of otolaryngology, 2005. **34**(6): p. 407-14.
260. Zhang, R., et al., *Osteogenic protein-1 enhances osseointegration of titanium implants coated with peri-apatite in rabbit femoral defect.* Journal of biomedical materials research. Part B, Applied biomaterials, 2004. **71**(2): p. 408-13.
261. Roldan, J.C., et al., *Bone formation in the presence of platelet-rich plasma vs. bone morphogenetic protein-7.* Bone, 2004. **34**(1): p. 80-90.
262. Corinaldesi, G., et al., *Augmentation of the floor of the maxillary sinus with recombinant human bone morphogenetic protein-7: a pilot radiological and histological study in humans.* The British journal of oral & maxillofacial surgery, 2013. **51**(3): p. 247-52.
263. Phillips, F.M., et al., *In vivo BMP-7 (OP-1) enhancement of osteoporotic vertebral bodies in an ovine model.* The spine journal : official journal of the North American Spine Society, 2006. **6**(5): p. 500-6.
264. Calori, G.M., et al., *Application of rhBMP-7 and platelet-rich plasma in the treatment of long bone non-unions: a prospective randomised clinical study on 120 patients.* Injury, 2008. **39**(12): p. 1391-402.
265. Calori, G.M., et al., *An ongoing research for evaluation of treatment with BMPs or AGFs in long bone non-union: protocol description and preliminary results.* Injury, 2006. **37 Suppl 3**: p. S43-50.
266. Kanakaris, N.K., et al., *Application of bone morphogenetic proteins to femoral non-unions: a 4-year multicentre experience.* Injury, 2009. **40 Suppl 3**: p. S54-61.
267. Giannoudis, P.V., et al., *The synergistic effect of autograft and BMP-7 in the treatment of atrophic nonunions.* Clin Orthop Relat Res, 2009. **467**(12): p. 3239-48.
268. Kanakaris, N.K., et al., *Application of BMP-7 to tibial non-unions: a 3-year multicenter experience.* Injury, 2008. **39 Suppl 2**: p. S83-90.
269. Ekrol, I., et al., *A comparison of RhBMP-7 (OP-1) and autogenous graft for metaphyseal defects after osteotomy of the distal radius.* Injury, 2008. **39 Suppl 2**: p. S73-82.
270. Bong, M.R., et al., *Osteogenic protein-1 (bone morphogenic protein-7) combined with various adjuncts in the treatment of humeral diaphyseal nonunions.* Bull Hosp Jt Dis, 2005. **63**(1-2): p. 20-3.

271. Lee, F.Y., et al., *Treatment of congenital pseudarthrosis of the tibia with recombinant human bone morphogenetic protein-7 (rhBMP-7). A report of five cases.* J Bone Joint Surg Am, 2006. **88**(3): p. 627-33.
272. Bilic, R., et al., *Osteogenic protein-1 (BMP-7) accelerates healing of scaphoid non-union with proximal pole sclerosis.* Int Orthop, 2006. **30**(2): p. 128-34.
273. Geesink, R.G., N.H. Hoefnagels, and S.K. Bulstra, *Osteogenic activity of OP-1 bone morphogenetic protein (BMP-7) in a human fibular defect.* J Bone Joint Surg Br, 1999. **81**(4): p. 710-8.
274. van den Bergh, J.P., et al., *Recombinant human bone morphogenetic protein-7 in maxillary sinus floor elevation surgery in 3 patients compared to autogenous bone grafts. A clinical pilot study.* J Clin Periodontol, 2000. **27**(9): p. 627-36.
275. Groeneveld, E.H., et al., *Histomorphometrical analysis of bone formed in human maxillary sinus floor elevations grafted with OP-1 device, demineralized bone matrix or autogenous bone. Comparison with non-grafted sites in a series of case reports.* Clin Oral Implants Res, 1999. **10**(6): p. 499-509.
276. Tan, H.C., et al., *Anti-fibrosis effect of BMP-7 peptide functionalization on cobalt chromium alloy.* J Orthop Res, 2013. **31**(6): p. 983-90.
277. Takigami, H., et al., *Bone formation following OP-1 implantation is improved by addition of autogenous bone marrow cells in a canine femur defect model.* Journal of orthopaedic research : official publication of the Orthopaedic Research Society, 2007. **25**(10): p. 1333-42.
278. Liao, J.C., et al., *Enhancement of recombinant human BMP-7 bone formation with bmp binding peptide in a rodent femoral defect model.* Journal of orthopaedic research : official publication of the Orthopaedic Research Society, 2011. **29**(5): p. 753-9.
279. Morgan, E.F., et al., *Combined effects of recombinant human BMP-7 (rhBMP-7) and parathyroid hormone (1-34) in metaphyseal bone healing.* Bone, 2008. **43**(6): p. 1031-8.
280. Im, G.I. and J.H. Lee, *Repair of osteochondral defects with adipose stem cells and a dual growth factor-releasing scaffold in rabbits.* Journal of biomedical materials research. Part B, Applied biomaterials, 2010. **92**(2): p. 552-60.
281. Little, D.G., et al., *Manipulation of the anabolic and catabolic responses with OP-1 and zoledronic acid in a rat critical defect model.* Journal of bone and mineral research : the official journal of the American Society for Bone and Mineral Research, 2005. **20**(11): p. 2044-52.
282. Zhang, Y., et al., *Synthesis and inflammatory response of a novel silk fibroin scaffold containing BMP7 adenovirus for bone regeneration.* Bone, 2012. **51**(4): p. 704-13.
283. Zhang, Y., et al., *In vitro and in vivo evaluation of adenovirus combined silk fibroin scaffolds for BMP-7 gene delivery.* Tissue engineering. Part C, Methods, 2011.
284. Dunn, C.A., et al., *BMP gene delivery for alveolar bone engineering at dental implant defects.* Molecular therapy : the journal of the American Society of Gene Therapy, 2005. **11**(2): p. 294-9.
285. Hu, J., et al., *Callus formation enhanced by BMP-7 ex vivo gene therapy during distraction osteogenesis in rats.* Journal of orthopaedic research : official publication of the Orthopaedic Research Society, 2007. **25**(2): p. 241-51.
286. Nussenbaum, B., R.B. Rutherford, and P.H. Krebsbach, *Bone regeneration in cranial defects previously treated with radiation.* The Laryngoscope, 2005. **115**(7): p. 1170-7.
287. Nussenbaum, B., et al., *Ex vivo gene therapy for skeletal regeneration in cranial defects compromised by postoperative radiotherapy.* Human gene therapy, 2003. **14**(11): p. 1107-15.
288. Jin, Q.M., et al., *Gene therapy of bone morphogenetic protein for periodontal tissue engineering.* Journal of periodontology, 2003. **74**(2): p. 202-13.
289. Rutherford, R.B., et al., *Bone morphogenetic protein-transduced human fibroblasts convert to osteoblasts and form bone in vivo.* Tissue engineering, 2002. **8**(3): p. 441-52.
290. Krebsbach, P.H., et al., *Gene therapy-directed osteogenesis: BMP-7-transduced human fibroblasts form bone in vivo.* Human gene therapy, 2000. **11**(8): p. 1201-10.
291. Zhang, Y., et al., *Delivery of PDGF-B and BMP-7 by mesoporous bioglass/silk fibrin scaffolds for the repair of osteoporotic defects.* Biomaterials, 2012. **33**(28): p. 6698-708.
292. Breitbart, A.S., et al., *Gene-enhanced tissue engineering: applications for bone healing using cultured periosteal cells transduced retrovirally with the BMP-7 gene.* Annals of plastic surgery, 1999. **42**(5): p. 488-95.
293. Kimelman-Bleich, N., et al., *Targeted gene-and-host progenitor cell therapy for nonunion bone fracture repair.* Molecular therapy : the journal of the American Society of Gene Therapy, 2011. **19**(1): p. 53-9.

294. Stavropoulos, A., et al., *A phase IIa randomized controlled clinical and histological pilot study evaluating rhGDF-5/beta-TCP for periodontal regeneration.* J Clin Periodontol, 2011. **38**(11): p. 1044-54.
295. Windisch, P., et al., *A phase IIa randomized controlled pilot study evaluating the safety and clinical outcomes following the use of rhGDF-5/beta-TCP in regenerative periodontal therapy.* Clin Oral Investig, 2012. **16**(4): p. 1181-9.
296. Stavropoulos, A., et al., *Histological evaluation of maxillary sinus floor augmentation with recombinant human growth and differentiation factor-5-coated beta-tricalcium phosphate: results of a multicenter randomized clinical trial.* J Clin Periodontol, 2011. **38**(10): p. 966-74.
297. Koch, F.P., et al., *A prospective, randomized pilot study on the safety and efficacy of recombinant human growth and differentiation factor-5 coated onto beta-tricalcium phosphate for sinus lift augmentation.* Clin Oral Implants Res, 2010. **21**(11): p. 1301-8.
298. Kleinschmidt, K., et al., *Superior angiogenic potential of GDF-5 and GDF-5(V453/V456) compared with BMP-2 in a rabbit long-bone defect model.* J Bone Joint Surg Am, 2014. **96**(20): p. 1699-707.
299. Degenkolbe, E., et al., *Improved bone defect healing by a superagonistic GDF5 variant derived from a patient with multiple synostoses syndrome.* Bone, 2015. **73**: p. 111-9.
300. Wan, D.C., et al., *Noggin suppression enhances in vitro osteogenesis and accelerates in vivo bone formation.* The Journal of biological chemistry, 2007. **282**(36): p. 26450-9.
301. Levi, B., et al., *Nonintegrating knockdown and customized scaffold design enhances human adipose-derived stem cells in skeletal repair.* Stem cells, 2011. **29**(12): p. 2018-29.
302. Levi, B., et al., *Enhancement of human adipose-derived stromal cell angiogenesis through knockdown of a BMP-2 inhibitor.* Plastic and reconstructive surgery, 2012. **129**(1): p. 53-66.
303. Lee, J.Y., et al., *Synthetic peptide-coated bone mineral for enhanced osteoblastic activation in vitro and in vivo.* J Biomed Mater Res A, 2008. **87**(3): p. 688-97.
304. Lee, J., et al., *SDF-1alpha/CXCR4 signaling mediates digit tip regeneration promoted by BMP-2.* Developmental biology, 2013. **382**(1): p. 98-109.
305. Ide, H., *Bone pattern formation in mouse limbs after amputation at the forearm level.* Developmental dynamics : an official publication of the American Association of Anatomists, 2012. **241**(3): p. 435-41.
306. Ren, W.H., L.J. Yang, and S.Z. Dong, *Induction of reparative dentin formation in dogs with combined recombinant human bone morphogenetic protein 2 and fibrin sealant.* The Chinese journal of dental research : the official journal of the Scientific Section of the Chinese Stomatological Association, 1999. **2**(3-4): p. 21-4.
307. Chung, I.H., et al., *Regulating the role of bone morphogenetic protein 4 in tooth bioengineering.* Journal of oral and maxillofacial surgery : official journal of the American Association of Oral and Maxillofacial Surgeons, 2007. **65**(3): p. 501-7.
308. Cooper, G.M., et al., *Noggin inhibits postoperative resynostosis in craniosynostotic rabbits.* Journal of bone and mineral research : the official journal of the American Society for Bone and Mineral Research, 2007. **22**(7): p. 1046-54.
309. Cray, J., Jr., et al., *Blocking bone morphogenetic protein function using in vivo noggin therapy does not rescue premature suture fusion in rabbits with delayed-onset craniosynostosis.* Plastic and reconstructive surgery, 2011. **127**(3): p. 1163-72.
310. Cooper, G.M., et al., *Ex vivo Noggin gene therapy inhibits bone formation in a mouse model of postoperative resynostosis.* Plast Reconstr Surg, 2009. **123**(2 Suppl): p. 94S-103S.
311. Kaplan, J., F.S. Kaplan, and E.M. Shore, *Restoration of normal BMP signaling levels and osteogenic differentiation in FOP mesenchymal progenitor cells by mutant allele-specific targeting.* Gene therapy, 2012. **19**(7): p. 786-90.
312. Takahashi, M., et al., *Disease-causing allele-specific silencing against the ALK2 mutants, R206H and G356D, in fibrodysplasia ossificans progressiva.* Gene therapy, 2012. **19**(7): p. 781-5.
313. Toyoda, H., et al., *Augmentation of bone morphogenetic protein-induced bone mass by local delivery of a prostaglandin E EP4 receptor agonist.* Bone, 2005. **37**(4): p. 555-62.
314. Sasaoka, R., et al., *A prostanoid receptor EP4 agonist enhances ectopic bone formation induced by recombinant human bone morphogenetic protein-2.* Biochem Biophys Res Commun, 2004. **318**(3): p. 704-9.
315. Nakamura, Y., et al., *Low dose fibroblast growth factor-2 (FGF-2) enhances bone morphogenetic protein-2 (BMP-2)-induced ectopic bone formation in mice.* Bone, 2005. **36**(3): p. 399-407.

316. Zhao, B., et al., *Heparin potentiates the in vivo ectopic bone formation induced by bone morphogenetic protein-2.* J Biol Chem, 2006. **281**(32): p. 23246-53.
317. Horiuchi, H., et al., *Effect of phosphodiesterase inhibitor-4, rolipram, on new bone formations by recombinant human bone morphogenetic protein-2.* Bone, 2002. **30**(4): p. 589-93.
318. Horiuchi, H., et al., *Enhancement of bone morphogenetic protein-2-induced new bone formation in mice by the phosphodiesterase inhibitor pentoxifylline.* Bone, 2001. **28**(3): p. 290-4.
319. Tokuhara, Y., et al., *Local delivery of rolipram, a phosphodiesterase-4-specific inhibitor, augments bone morphogenetic protein-induced bone formation.* J Bone Miner Metab, 2010. **28**(1): p. 17-24.
320. Schindeler, A., et al., *Rapid cell culture and pre-clinical screening of a transforming growth factor-beta (TGF-beta) inhibitor for orthopaedics.* BMC Musculoskelet Disord, 2010. **11**: p. 105.
321. Brochmann, E.J., et al., *Carboxy terminus of secreted phosphoprotein-24 kDa (spp24) is essential for full inhibition of BMP-2 activity.* J Orthop Res, 2010. **28**(9): p. 1200-7.
322. Sintuu, C., et al., *Full-length bovine spp24 [spp24 (24-203)] inhibits BMP-2 induced bone formation.* J Orthop Res, 2008. **26**(6): p. 753-8.
323. Mori, S., et al., *Antiangiogenic agent (TNP-470) inhibition of ectopic bone formation induced by bone morphogenetic protein-2.* Bone, 1998. **22**(2): p. 99-105.
324. Yoshikawa, H., et al., *Stimulation of ectopic bone formation in response to BMP-2 by Rho kinase inhibitor: a pilot study.* Clin Orthop Relat Res, 2009. **467**(12): p. 3087-95.
325. Zhao, M., et al., *Combinatorial gene therapy for bone regeneration: cooperative interactions between adenovirus vectors expressing bone morphogenetic proteins 2, 4, and 7.* J Cell Biochem, 2005. **95**(1): p. 1-16.
326. Kaihara, S., et al., *Simple and effective osteoinductive gene therapy by local injection of a bone morphogenetic protein-2-expressing recombinant adenoviral vector and FK506 mixture in rats.* Gene Ther, 2004. **11**(5): p. 439-47.
327. Hannallah, D., et al., *Retroviral delivery of Noggin inhibits the formation of heterotopic ossification induced by BMP-4, demineralized bone matrix, and trauma in an animal model.* J Bone Joint Surg Am, 2004. **86-A**(1): p. 80-91.
328. Lee, S.J., et al., *Enhancement of bone regeneration by gene delivery of BMP2/Runx2 bicistronic vector into adipose-derived stromal cells.* Biomaterials, 2010. **31**(21): p. 5652-9.
329. Samee, M., et al., *Bone morphogenetic protein-2 (BMP-2) and vascular endothelial growth factor (VEGF) transfection to human periosteal cells enhances osteoblast differentiation and bone formation.* J Pharmacol Sci, 2008. **108**(1): p. 18-31.
330. Huang, Y.C., et al., *Combined angiogenic and osteogenic factor delivery enhances bone marrow stromal cell-driven bone regeneration.* J Bone Miner Res, 2005. **20**(5): p. 848-57.
331. Yu, P.B., et al., *BMP type I receptor inhibition reduces heterotopic [corrected] ossification.* Nat Med, 2008. **14**(12): p. 1363-9.
332. Peterson, J.R., et al., *Treatment of heterotopic ossification through remote ATP hydrolysis.* Sci Transl Med, 2014. **6**(255): p. 255ra132.
333. Mohedas, A.H., et al., *Development of an ALK2-biased BMP type I receptor kinase inhibitor.* ACS Chem Biol, 2013. **8**(6): p. 1291-302.
334. Glaser, D.L., et al., *In vivo somatic cell gene transfer of an engineered Noggin mutein prevents BMP4-induced heterotopic ossification.* J Bone Joint Surg Am, 2003. **85-A**(12): p. 2332-42.
335. Yamamoto, R., et al., *Clinically applicable antianginal agents suppress osteoblastic transformation of myogenic cells and heterotopic ossifications in mice.* J Bone Miner Metab, 2013. **31**(1): p. 26-33.
336. Shimono, K., et al., *Potent inhibition of heterotopic ossification by nuclear retinoic acid receptor-gamma agonists.* Nat Med, 2011. **17**(4): p. 454-60.
337. Leckie, S.K., et al., *Injection of AAV2-BMP2 and AAV2-TIMP1 into the nucleus pulposus slows the course of intervertebral disc degeneration in an in vivo rabbit model.* Spine J, 2012. **12**(1): p. 7-20.
338. Di Cesare, P.E., et al., *Regional gene therapy for full-thickness articular cartilage lesions using naked DNA with a collagen matrix.* J Orthop Res, 2006. **24**(5): p. 1118-27.
339. Kawakami, M., et al., *Osteogenic protein-1 (osteogenic protein-1/bone morphogenetic protein-7) inhibits degeneration and pain-related behavior induced by chronically compressed nucleus pulposus in the rat.* Spine (Phila Pa 1976), 2005. **30**(17): p. 1933-9.
340. Che, J.H., et al., *Application of tissue-engineered cartilage with BMP-7 gene to repair knee joint cartilage injury in rabbits.* Knee Surg Sports Traumatol Arthrosc, 2010. **18**(4): p. 496-503.

341. Chaofeng, W., et al., *Nucleus pulposus cells expressing hBMP7 can prevent the degeneration of allogenic IVD in a canine transplantation model.* J Orthop Res, 2013. **31**(9): p. 1366-73.
342. Chujo, T., et al., *Effects of growth differentiation factor-5 on the intervertebral disc--in vitro bovine study and in vivo rabbit disc degeneration model study.* Spine (Phila Pa 1976), 2006. **31**(25): p. 2909-17.
343. Fourman, M.S., et al., *Recombinant human BMP-2 increases the incidence and rate of healing in complex ankle arthrodesis.* Clinical orthopaedics and related research, 2014. **472**(2): p. 732-9.
344. Majid, K., et al., *Biomimetic calcium phosphate coatings as bone morphogenetic protein delivery systems in spinal fusion.* The spine journal : official journal of the North American Spine Society, 2011. **11**(6): p. 560-7.
345. Helm, G.A., et al., *Utilization of type I collagen gel, demineralized bone matrix, and bone morphogenetic protein-2 to enhance autologous bone lumbar spinal fusion.* Journal of neurosurgery, 1997. **86**(1): p. 93-100.
346. Meyer, R.A., Jr., et al., *Safety of recombinant human bone morphogenetic protein-2 after spinal laminectomy in the dog.* Spine, 1999. **24**(8): p. 747-54.
347. David, S.M., et al., *Lumbar spinal fusion using recombinant human bone morphogenetic protein in the canine. A comparison of three dosages and two carriers.* Spine, 1999. **24**(19): p. 1973-9.
348. Martin, G.J., Jr., S.D. Boden, and L. Titus, *Recombinant human bone morphogenetic protein-2 overcomes the inhibitory effect of ketorolac, a nonsteroidal anti-inflammatory drug (NSAID), on posterolateral lumbar intertransverse process spine fusion.* Spine, 1999. **24**(21): p. 2188-93; discussion 2193-4.
349. Sandhu, H.S., et al., *Histologic evaluation of the efficacy of rhBMP-2 compared with autograft bone in sheep spinal anterior interbody fusion.* Spine, 2002. **27**(6): p. 567-75.
350. Akamaru, T., et al., *Simple carrier matrix modifications can enhance delivery of recombinant human bone morphogenetic protein-2 for posterolateral spine fusion.* Spine, 2003. **28**(5): p. 429-34.
351. Zhang, H., D.J. Sucato, and R.D. Welch, *Recombinant human bone morphogenic protein-2-enhanced anterior spine fusion without bone encroachment into the spinal canal: a histomorphometric study in a thoracoscopically instrumented porcine model.* Spine, 2005. **30**(5): p. 512-8.
352. Glassman, S.D., et al., *The efficacy of rhBMP-2 for posterolateral lumbar fusion in smokers.* Spine, 2007. **32**(15): p. 1693-8.
353. Oluigbo, C.O. and G.A. Solanki, *Use of recombinant human bone morphogenetic protein-2 to enhance posterior cervical spine fusion at 2 years of age: technical note.* Pediatric neurosurgery, 2008. **44**(5): p. 393-6.
354. Miyazaki, M., et al., *A porcine collagen-derived matrix as a carrier for recombinant human bone morphogenetic protein-2 enhances spinal fusion in rats.* The spine journal : official journal of the North American Spine Society, 2009. **9**(1): p. 22-30.
355. Shen, H.X., et al., *Pseudarthrosis in multilevel anterior cervical fusion with rhBMP-2 and allograft: analysis of one hundred twenty-seven cases with minimum two-year follow-up.* Spine, 2010. **35**(7): p. 747-53.
356. Xu, R., et al., *Safety and efficacy of rhBMP2 in posterior cervical spinal fusion for subaxial degenerative spine disease: Analysis of outcomes in 204 patients.* Surgical neurology international, 2011. **2**: p. 109.
357. Williams, B.J., et al., *Does bone morphogenetic protein increase the incidence of perioperative complications in spinal fusion? A comparison of 55,862 cases of spinal fusion with and without bone morphogenetic protein.* Spine, 2011. **36**(20): p. 1685-91.
358. Koo, K.H., et al., *Lumbar posterolateral fusion using heparin-conjugated fibrin for sustained delivery of bone morphogenic protein-2 in a rabbit model.* Artificial organs, 2012. **36**(7): p. 629-34.
359. Wang, M., et al., *Minimizing the severity of rhBMP-2-induced inflammation and heterotopic ossification with a polyelectrolyte carrier incorporating heparin on microbead templates.* Spine, 2013. **38**(17): p. 1452-8.
360. Koo, K.H., et al., *Controlled delivery of low-dose bone morphogenetic protein-2 using heparin-conjugated fibrin in the posterolateral lumbar fusion of rabbits.* Artificial organs, 2013. **37**(5): p. 487-94.
361. Flouzat-Lachaniette, C.H., et al., *Bone union rate with recombinant human bone morphogenic protein-2 versus autologous iliac bone in PEEK cages for anterior lumbar interbody fusion.* International orthopaedics, 2014.
362. Wang, H., et al., *Osteoinductive activity of ErhBMP-2 after anterior cervical diskectomy and fusion with a ss-TCP interbody cage in a goat model.* Orthopedics, 2014. **37**(2): p. e123-31.

363. Lee, S.S., et al., *Gel Scaffolds of BMP-2-Binding Peptide Amphiphile Nanofibers for Spinal Arthrodesis.* Advanced healthcare materials, 2014.
364. Roh, J.S., et al., *Allogeneic morphogenetic protein vs. recombinant human bone morphogenetic protein-2 in lumbar interbody fusion procedures: a radiographic and economic analysis.* J Orthop Surg Res, 2013. **8**: p. 49.
365. Pimenta, L., et al., *A prospective, randomized, controlled trial comparing radiographic and clinical outcomes between stand-alone lateral interbody lumbar fusion with either silicate calcium phosphate or rh-BMP2.* J Neurol Surg A Cent Eur Neurosurg, 2013. **74**(6): p. 343-50.
366. Hodges, S.D., J.C. Eck, and D. Newton, *Retrospective study of posterior cervical fusions with rhBMP-2.* Orthopedics, 2012. **35**(6): p. e895-8.
367. Sethi, A., et al., *Radiographic and CT evaluation of recombinant human bone morphogenetic protein-2-assisted spinal interbody fusion.* AJR Am J Roentgenol, 2011. **197**(1): p. W128-33.
368. Singh, K., et al., *Repeat use of human recombinant bone morphogenetic protein-2 for second level lumbar arthrodesis.* Spine (Phila Pa 1976), 2011. **36**(3): p. 192-6.
369. Rogozinski, A., C. Rogozinski, and G. Cloud, *Accelerating autograft maturation in instrumented posterolateral lumbar spinal fusions without donor site morbidity.* Orthopedics, 2009. **32**(11): p. 809.
370. Dawson, E., et al., *Recombinant human bone morphogenetic protein-2 on an absorbable collagen sponge with an osteoconductive bulking agent in posterolateral arthrodesis with instrumentation. A prospective randomized trial.* J Bone Joint Surg Am, 2009. **91**(7): p. 1604-13.
371. Dimar, J.R., 2nd, et al., *Clinical and radiographic analysis of an optimized rhBMP-2 formulation as an autograft replacement in posterolateral lumbar spine arthrodesis.* J Bone Joint Surg Am, 2009. **91**(6): p. 1377-86.
372. Burkus, J.K., et al., *Six-year outcomes of anterior lumbar interbody arthrodesis with use of interbody fusion cages and recombinant human bone morphogenetic protein-2.* J Bone Joint Surg Am, 2009. **91**(5): p. 1181-9.
373. Carreon, L.Y., et al., *RhBMP-2 versus iliac crest bone graft for lumbar spine fusion in patients over 60 years of age: a cost-utility study.* Spine (Phila Pa 1976), 2009. **34**(3): p. 238-43.
374. Glassman, S.D., et al., *RhBMP-2 versus iliac crest bone graft for lumbar spine fusion: a randomized, controlled trial in patients over sixty years of age.* Spine (Phila Pa 1976), 2008. **33**(26): p. 2843-9.
375. Vaidya, R., et al., *Complications in the use of rhBMP-2 in PEEK cages for interbody spinal fusions.* J Spinal Disord Tech, 2008. **21**(8): p. 557-62.
376. Meisel, H.J., et al., *Posterior lumbar interbody fusion using rhBMP-2.* Eur Spine J, 2008. **17**(12): p. 1735-44.
377. Mulconrey, D.S., et al., *Bone morphogenetic protein (RhBMP-2) as a substitute for iliac crest bone graft in multilevel adult spinal deformity surgery: minimum two-year evaluation of fusion.* Spine (Phila Pa 1976), 2008. **33**(20): p. 2153-9.
378. Katayama, Y., et al., *Clinical and radiographic outcomes of posterolateral lumbar spine fusion in humans using recombinant human bone morphogenetic protein-2: an average five-year follow-up study.* Int Orthop, 2009. **33**(4): p. 1061-7.
379. Buttermann, G.R., *Prospective nonrandomized comparison of an allograft with bone morphogenic protein versus an iliac-crest autograft in anterior cervical discectomy and fusion.* Spine J, 2008. **8**(3): p. 426-35.
380. Slosar, P.J., R. Josey, and J. Reynolds, *Accelerating lumbar fusions by combining rhBMP-2 with allograft bone: a prospective analysis of interbody fusion rates and clinical outcomes.* Spine J, 2007. **7**(3): p. 301-7.
381. Smucker, J.D., et al., *Increased swelling complications associated with off-label usage of rhBMP-2 in the anterior cervical spine.* Spine (Phila Pa 1976), 2006. **31**(24): p. 2813-9.
382. Dimar, J.R., et al., *Clinical outcomes and fusion success at 2 years of single-level instrumented posterolateral fusions with recombinant human bone morphogenetic protein-2/compression resistant matrix versus iliac crest bone graft.* Spine (Phila Pa 1976), 2006. **31**(22): p. 2534-9; discussion 2540.
383. Anand, N., et al., *Cantilever TLIF with structural allograft and RhBMP2 for correction and maintenance of segmental sagittal lordosis: long-term clinical, radiographic, and functional outcome.* Spine (Phila Pa 1976), 2006. **31**(20): p. E748-53.
384. Burkus, J.K., H.S. Sandhu, and M.F. Gornet, *Influence of rhBMP-2 on the healing patterns associated with allograft interbody constructs in comparison with autograft.* Spine (Phila Pa 1976), 2006. **31**(7): p. 775-81.

385. Villavicencio, A.T., et al., *Safety of transforaminal lumbar interbody fusion and intervertebral recombinant human bone morphogenetic protein-2.* J Neurosurg Spine, 2005. **3**(6): p. 436-43.
386. Luhmann, S.J., et al., *Use of bone morphogenetic protein-2 for adult spinal deformity.* Spine (Phila Pa 1976), 2005. **30**(17 Suppl): p. S110-7.
387. Glassman, S.D., et al., *Initial fusion rates with recombinant human bone morphogenetic protein-2/compression resistant matrix and a hydroxyapatite and tricalcium phosphate/collagen carrier in posterolateral spinal fusion.* Spine (Phila Pa 1976), 2005. **30**(15): p. 1694-8.
388. Boakye, M., et al., *Anterior cervical discectomy and fusion involving a polyetheretherketone spacer and bone morphogenetic protein.* J Neurosurg Spine, 2005. **2**(5): p. 521-5.
389. Burkus, J.K., et al., *Use of rhBMP-2 in combination with structural cortical allografts: clinical and radiographic outcomes in anterior lumbar spinal surgery.* J Bone Joint Surg Am, 2005. **87**(6): p. 1205-12.
390. Haid, R.W., Jr., et al., *Posterior lumbar interbody fusion using recombinant human bone morphogenetic protein type 2 with cylindrical interbody cages.* Spine J, 2004. **4**(5): p. 527-38; discussion 538-9.
391. Mummaneni, P.V., et al., *Contribution of recombinant human bone morphogenetic protein-2 to the rapid creation of interbody fusion when used in transforaminal lumbar interbody fusion: a preliminary report. Invited submission from the Joint Section Meeting on Disorders of the Spine and Peripheral Nerves, March 2004.* J Neurosurg Spine, 2004. **1**(1): p. 19-23.
392. Kuklo, T.R., M.K. Rosner, and D.W. Polly, Jr., *Computerized tomography evaluation of a resorbable implant after transforaminal lumbar interbody fusion.* Neurosurg Focus, 2004. **16**(3): p. E10.
393. Lanman, T.H. and T.J. Hopkins, *Lumbar interbody fusion after treatment with recombinant human bone morphogenetic protein-2 added to poly(L-lactide-co-D,L-lactide) bioresorbable implants.* Neurosurg Focus, 2004. **16**(3): p. E9.
394. Baskin, D.S., et al., *A prospective, randomized, controlled cervical fusion study using recombinant human bone morphogenetic protein-2 with the CORNERSTONE-SR allograft ring and the ATLANTIS anterior cervical plate.* Spine (Phila Pa 1976), 2003. **28**(12): p. 1219-24; discussion 1225.
395. Burkus, J.K., J.D. Dorchak, and D.L. Sanders, *Radiographic assessment of interbody fusion using recombinant human bone morphogenetic protein type 2.* Spine (Phila Pa 1976), 2003. **28**(4): p. 372-7.
396. Boden, S.D., et al., *Use of recombinant human bone morphogenetic protein-2 to achieve posterolateral lumbar spine fusion in humans: a prospective, randomized clinical pilot trial: 2002 Volvo Award in clinical studies.* Spine (Phila Pa 1976), 2002. **27**(23): p. 2662-73.
397. Burkus, J.K., et al., *Clinical and radiographic outcomes of anterior lumbar interbody fusion using recombinant human bone morphogenetic protein-2.* Spine (Phila Pa 1976), 2002. **27**(21): p. 2396-408.
398. Burkus, J.K., et al., *Anterior lumbar interbody fusion using rhBMP-2 with tapered interbody cages.* J Spinal Disord Tech, 2002. **15**(5): p. 337-49.
399. Boden, S.D., et al., *The use of rhBMP-2 in interbody fusion cages. Definitive evidence of osteoinduction in humans: a preliminary report.* Spine (Phila Pa 1976), 2000. **25**(3): p. 376-81.
400. Corenman, D.S., et al., *Recombinant human bone morphogenetic protein-2-augmented transforaminal lumbar interbody fusion for the treatment of chronic low back pain secondary to the homogeneous diagnosis of discogenic pain syndrome: two-year outcomes.* Spine (Phila Pa 1976), 2013. **38**(20): p. E1269-77.
401. Michielsen, J., et al., *The effect of recombinant human bone morphogenetic protein-2 in single-level posterior lumbar interbody arthrodesis.* J Bone Joint Surg Am, 2013. **95**(10): p. 873-80.
402. Kaito, T., et al., *Synergistic effect of bone morphogenetic proteins 2 and 7 by ex vivo gene therapy in a rat spinal fusion model.* The Journal of bone and joint surgery. American volume, 2013. **95**(17): p. 1612-9.
403. Refaat, M., et al., *Binding to COMP Reduces the BMP2 Dose for Spinal Fusion in a Rat Model.* Spine (Phila Pa 1976), 2015.
404. Morimoto, T., et al., *Effect of Intermittent Administration of Teriparatide (Parathyroid Hormone 1-34) on Bone Morphogenetic Protein-Induced Bone Formation in a Rat Model of Spinal Fusion.* J Bone Joint Surg Am, 2014. **96**(13): p. e107.
405. Fu, T.S., et al., *Enhancement of posterolateral lumbar spine fusion using low-dose rhBMP-2 and cultured marrow stromal cells.* Journal of orthopaedic research : official publication of the Orthopaedic Research Society, 2009. **27**(3): p. 380-4.

406. Gressot, L.V., et al., *Rh-BMP-2 for L5-S1 arthrodesis in long fusions to the pelvis for neuromuscular spinal deformity in the pediatric age group: analysis of 11 patients.* Child's nervous system : ChNS : official journal of the International Society for Pediatric Neurosurgery, 2014. **30**(2): p. 249-55.
407. Park, S.B., et al., *BMP-2 induced early bone formation in spine fusion using rat ovariectomy osteoporosis model.* The spine journal : official journal of the North American Spine Society, 2013. **13**(10): p. 1273-80.
408. Namikawa, T., et al., *Enhancing effects of a prostaglandin EP4 receptor agonist on recombinant human bone morphogenetic protein-2 mediated spine fusion in a rabbit model.* Spine, 2007. **32**(21): p. 2294-9.
409. Minamide, A., et al., *The effects of bone morphogenetic protein and basic fibroblast growth factor on cultured mesenchymal stem cells for spine fusion.* Spine, 2007. **32**(10): p. 1067-71.
410. Alden, T.D., et al., *Bone morphogenetic protein gene therapy for the induction of spinal arthrodesis.* Neurosurgical focus, 1998. **4**(2): p. e12.
411. Alden, T.D., et al., *Percutaneous spinal fusion using bone morphogenetic protein-2 gene therapy.* Journal of neurosurgery, 1999. **90**(1 Suppl): p. 109-14.
412. Miyazaki, M., et al., *Comparison of human mesenchymal stem cells derived from adipose tissue and bone marrow for ex vivo gene therapy in rat spinal fusion model.* Spine, 2008. **33**(8): p. 863-9.
413. Miyazaki, M., et al., *The effects of lentiviral gene therapy with bone morphogenetic protein-2-producing bone marrow cells on spinal fusion in rats.* Journal of spinal disorders & techniques, 2008. **21**(5): p. 372-9.
414. Miyazaki, M., et al., *Comparison of lentiviral and adenoviral gene therapy for spinal fusion in rats.* Spine, 2008. **33**(13): p. 1410-7.
415. Hasharoni, A., et al., *Murine spinal fusion induced by engineered mesenchymal stem cells that conditionally express bone morphogenetic protein-2.* Journal of neurosurgery. Spine, 2005. **3**(1): p. 47-52.
416. Wang, J.C., et al., *Effect of regional gene therapy with bone morphogenetic protein-2-producing bone marrow cells on spinal fusion in rats.* The Journal of bone and joint surgery. American volume, 2003. **85-A**(5): p. 905-11.
417. Riew, K.D., et al., *Thoracoscopic intradiscal spine fusion using a minimally invasive gene-therapy technique.* The Journal of bone and joint surgery. American volume, 2003. **85-A**(5): p. 866-71.
418. Riew, K.D., et al., *Induction of bone formation using a recombinant adenoviral vector carrying the human BMP-2 gene in a rabbit spinal fusion model.* Calcified tissue international, 1998. **63**(4): p. 357-60.
419. Lee, T.C., et al., *Bone morphogenetic protein gene therapy using a fibrin scaffold for a rabbit spinal-fusion experiment.* Neurosurgery, 2006. **58**(2): p. 373-80; discussion 373-80.
420. Smucker, J.D., et al., *B2A peptide on ceramic granules enhance posterolateral spinal fusion in rabbits compared with autograft.* Spine (Phila Pa 1976), 2008. **33**(12): p. 1324-9.
421. Cunningham, B.W., et al., *Ceramic granules enhanced with B2A peptide for lumbar interbody spine fusion: an experimental study using an instrumented model in sheep.* J Neurosurg Spine, 2009. **10**(4): p. 300-7.
422. Sardar, Z., et al., *Twelve-month results of a multicenter, blinded, pilot study of a novel peptide (B2A) in promoting lumbar spine fusion.* J Neurosurg Spine, 2015. **22**(4): p. 358-66.
423. Cheng, J.C., et al., *How does recombinant human bone morphogenetic protein-4 enhance posterior spinal fusion?* Spine, 2002. **27**(5): p. 467-74.
424. Guo, X., et al., *Recombinant human bone morphogenetic protein-4 (rhBMP-4) enhanced posterior spinal fusion without decortication.* Journal of orthopaedic research : official publication of the Orthopaedic Research Society, 2002. **20**(4): p. 740-6.
425. Zhao, J., et al., *Promoting lumbar spinal fusion by adenovirus-mediated bone morphogenetic protein-4 gene therapy.* Chin J Traumatol, 2007. **10**(2): p. 72-6.
426. Valdes, M., et al., *rhBMP-6 stimulated osteoprogenitor cells enhance posterolateral spinal fusion in the New Zealand white rabbit.* The spine journal : official journal of the North American Spine Society, 2007. **7**(3): p. 318-25.
427. Laurent, J.J., et al., *The use of bone morphogenetic protein-6 gene therapy for percutaneous spinal fusion in rabbits.* J Neurosurg Spine, 2004. **1**(1): p. 90-4.
428. Lu, J., et al., *Posterolateral intertransverse spinal fusion possible in osteoporotic rats with BMP-7 in a higher dose delivered on a composite carrier.* Spine, 2008. **33**(3): p. 242-9.

429. Vaccaro, A.R., D.G. Anderson, and C.A. Toth, *Recombinant human osteogenic protein-1 (bone morphogenetic protein-7) as an osteoinductive agent in spinal fusion.* Spine, 2002. **27**(16 Suppl 1): p. S59-65.
430. Salamon, M.L., et al., *The effects of BMP-7 in a rat posterolateral intertransverse process fusion model.* Journal of spinal disorders & techniques, 2003. **16**(1): p. 90-5.
431. Jenis, L.G., et al., *The effect of osteogenic protein-1 in instrumented and noninstrumented posterolateral fusion in rabbits.* The spine journal : official journal of the North American Spine Society, 2002. **2**(3): p. 173-8.
432. Mermer, M.J., et al., *Efficacy of osteogenic protein-1 in a challenging multilevel fusion model.* Spine, 2004. **29**(3): p. 249-56.
433. Delawi, D., et al., *A prospective, randomized, controlled, multicenter study of osteogenic protein-1 in instrumented posterolateral fusions: report on safety and feasibility.* Spine (Phila Pa 1976), 2010. **35**(12): p. 1185-91.
434. Furlan, J.C., et al., *Use of osteogenic protein-1 in patients at high risk for spinal pseudarthrosis: a prospective cohort study assessing safety, health-related quality of life, and radiographic fusion. Invited submission from the Joint Section on Disorders of the Spine and Peripheral Nerves, March 2007.* J Neurosurg Spine, 2007. **7**(5): p. 486-95.
435. Vaccaro, A.R., et al., *The safety and efficacy of OP-1 (rhBMP-7) as a replacement for iliac crest autograft for posterolateral lumbar arthrodesis: minimum 4-year follow-up of a pilot study.* Spine J, 2008. **8**(3): p. 457-65.
436. Kanayama, M., et al., *A prospective randomized study of posterolateral lumbar fusion using osteogenic protein-1 (OP-1) versus local autograft with ceramic bone substitute: emphasis of surgical exploration and histologic assessment.* Spine (Phila Pa 1976), 2006. **31**(10): p. 1067-74.
437. Vaccaro, A.R., et al., *Comparison of OP-1 Putty (rhBMP-7) to iliac crest autograft for posterolateral lumbar arthrodesis: a minimum 2-year follow-up pilot study.* Spine (Phila Pa 1976), 2005. **30**(24): p. 2709-16.
438. Vaccaro, A.R., et al., *A 2-year follow-up pilot study evaluating the safety and efficacy of op-1 putty (rhbmp-7) as an adjunct to iliac crest autograft in posterolateral lumbar fusions.* Eur Spine J, 2005. **14**(7): p. 623-9.
439. Vaccaro, A.R., et al., *A pilot study evaluating the safety and efficacy of OP-1 Putty (rhBMP-7) as a replacement for iliac crest autograft in posterolateral lumbar arthrodesis for degenerative spondylolisthesis.* Spine (Phila Pa 1976), 2004. **29**(17): p. 1885-92.
440. Vaccaro, A.R., et al., *A pilot safety and efficacy study of OP-1 putty (rhBMP-7) as an adjunct to iliac crest autograft in posterolateral lumbar fusions.* Eur Spine J, 2003. **12**(5): p. 495-500.
441. Taghavi, C.E., et al., *Bone morphogenetic protein binding peptide mechanism and enhancement of osteogenic protein-1 induced bone healing.* Spine, 2010. **35**(23): p. 2049-56.
442. Hidaka, C., et al., *Enhancement of spine fusion using combined gene therapy and tissue engineering BMP-7-expressing bone marrow cells and allograft bone.* Spine (Phila Pa 1976), 2003. **28**(18): p. 2049-57.
443. Helm, G.A., et al., *Use of bone morphogenetic protein-9 gene therapy to induce spinal arthrodesis in the rodent.* J Neurosurg, 2000. **92**(2 Suppl): p. 191-6.
444. Dumont, R.J., et al., *Ex vivo bone morphogenetic protein-9 gene therapy using human mesenchymal stem cells induces spinal fusion in rodents.* Neurosurgery, 2002. **51**(5): p. 1239-44; discussion 1244-5.
445. Baud'huin, M., et al., *A soluble bone morphogenetic protein type IA receptor increases bone mass and bone strength.* Proc Natl Acad Sci U S A, 2012. **109**(30): p. 12207-12.
446. Turgeman, G., et al., *Systemically administered rhBMP-2 promotes MSC activity and reverses bone and cartilage loss in osteopenic mice.* Journal of cellular biochemistry, 2002. **86**(3): p. 461-74.
447. Zerath, E., et al., *Effects of BMP-2 on osteoblastic cells and on skeletal growth and bone formation in unloaded rats.* Growth hormone & IGF research : official journal of the Growth Hormone Research Society and the International IGF Research Society, 1998. **8**(2): p. 141-9.
448. Dumic-Cule, I., et al., *Systemically available bone morphogenetic protein two and seven affect bone metabolism.* Int Orthop, 2014. **38**(9): p. 1979-85.
449. Kumar, S., T.R. Nagy, and S. Ponnazhagan, *Therapeutic potential of genetically modified adult stem cells for osteopenia.* Gene therapy, 2010. **17**(1): p. 105-16.

450. Kumar, S., et al., *Osteogenic differentiation of recombinant adeno-associated virus 2-transduced murine mesenchymal stem cells and development of an immunocompetent mouse model for ex vivo osteoporosis gene therapy.* Human gene therapy, 2004. **15**(12): p. 1197-206.
451. Hsiao, W.K., et al., *Intramarrow bone morphogenetic protein 4 gene delivery improves local bone quality in femurs of ovariectomized rabbits.* J Periodontol, 2011. **82**(6): p. 854-62.
452. Zhang, X.S., et al., *Local ex vivo gene therapy with bone marrow stromal cells expressing human BMP4 promotes endosteal bone formation in mice.* J Gene Med, 2004. **6**(1): p. 4-15.
453. Simic, P., et al., *Systemically administered bone morphogenetic protein-6 restores bone in aged ovariectomized rats by increasing bone formation and suppressing bone resorption.* J Biol Chem, 2006. **281**(35): p. 25509-21.
454. Gonzalez, E.A., et al., *Treatment of a murine model of high-turnover renal osteodystrophy by exogenous BMP-7.* Kidney Int, 2002. **61**(4): p. 1322-31.
455. Shim, J.H., et al., *Administration of BMP2/7 in utero partially reverses Rubinstein-Taybi syndrome-like skeletal defects induced by Pdk1 or Cbp mutations in mice.* The Journal of clinical investigation, 2012. **122**(1): p. 91-106.
456. Kim, J.G., et al., *Enhancement of tendon-bone healing with the use of bone morphogenetic protein-2 inserted into the suture anchor hole in a rabbit patellar tendon model.* Cytotherapy, 2014. **16**(6): p. 857-67.
457. Ma, C.B., et al., *Bone morphogenetic proteins-signaling plays a role in tendon-to-bone healing: a study of rhBMP-2 and noggin.* Am J Sports Med, 2007. **35**(4): p. 597-604.
458. Rodeo, S.A., et al., *Use of recombinant human bone morphogenetic protein-2 to enhance tendon healing in a bone tunnel.* Am J Sports Med, 1999. **27**(4): p. 476-88.
459. Chen, C.H., et al., *Enhancement of rotator cuff tendon-bone healing with injectable periosteum progenitor cells-BMP-2 hydrogel in vivo.* Knee Surg Sports Traumatol Arthrosc, 2011. **19**(9): p. 1597-607.
460. Chen, C.H., et al., *Photoencapsulation of bone morphogenetic protein-2 and periosteal progenitor cells improve tendon graft healing in a bone tunnel.* Am J Sports Med, 2008. **36**(3): p. 461-73.
461. Martinek, V., et al., *Enhancement of tendon-bone integration of anterior cruciate ligament grafts with bone morphogenetic protein-2 gene transfer: a histological and biomechanical study.* J Bone Joint Surg Am, 2002. **84-A**(7): p. 1123-31.
462. Hoffmann, A., et al., *Neotendon formation induced by manipulation of the Smad8 signalling pathway in mesenchymal stem cells.* J Clin Invest, 2006. **116**(4): p. 940-52.
463. Wang, C.J., et al., *pCMV-BMP-2-transfected cell-mediated gene therapy in anterior cruciate ligament reconstruction in rabbits.* Arthroscopy, 2010. **26**(7): p. 968-76.
464. Coen, M.J., et al., *Lentiviral-based BMP4 in vivo gene transfer strategy increases pull-out tensile strength without an improvement in the osteointegration of the tendon graft in a rat model of biceps tenodesis.* J Gene Med, 2011. **13**(10): p. 511-21.
465. Aspenberg, P. and C. Forslund, *Bone morphogenetic proteins and tendon repair.* Scand J Med Sci Sports, 2000. **10**(6): p. 372-5.
466. Dines, J.S., et al., *The effect of growth differentiation factor-5-coated sutures on tendon repair in a rat model.* J Shoulder Elbow Surg, 2007. **16**(5 Suppl): p. S215-21.
467. Bolt, P., et al., *BMP-14 gene therapy increases tendon tensile strength in a rat model of Achilles tendon injury.* J Bone Joint Surg Am, 2007. **89**(6): p. 1315-20.
468. Lamplot, J.D., et al., *Distinct effects of platelet-rich plasma and BMP13 on rotator cuff tendon injury healing in a rat model.* Am J Sports Med, 2014. **42**(12): p. 2877-87.
469. Greiner, S., et al., *Local rhBMP-12 on an Absorbable Collagen Sponge as an Adjuvant Therapy for Rotator Cuff Repair - A Phase 1, Randomized, Standard of Care Control, Multicenter Study: Safety and Feasibility.* Am J Sports Med, 2015. **43**(8): p. 1994-2004.
470. Chamberlain, C.S., et al., *Effects of BMP-12-releasing sutures on Achilles tendon healing.* Tissue Eng Part A, 2015. **21**(5-6): p. 916-27.
471. Majewski, M., et al., *Ex vivo adenoviral transfer of bone morphogenetic protein 12 (BMP-12) cDNA improves Achilles tendon healing in a rat model.* Gene Ther, 2008. **15**(16): p. 1139-46.
472. Lories, R.J., I. Derese, and F.P. Luyten, *Modulation of bone morphogenetic protein signaling inhibits the onset and progression of ankylosing enthesitis.* J Clin Invest, 2005. **115**(6): p. 1571-9.
473. Tandon, A., et al., *BMP7 gene transfer via gold nanoparticles into stroma inhibits corneal fibrosis in vivo.* PLoS One, 2013. **8**(6): p. e66434.

474. Saika, S., et al., *Therapeutic effects of adenoviral gene transfer of bone morphogenic protein-7 on a corneal alkali injury model in mice.* Lab Invest, 2005. **85**(4): p. 474-86.
475. Zhong, L., et al., *The anti-fibrotic effect of bone morphogenic protein-7(BMP-7) on liver fibrosis.* Int J Med Sci, 2013. **10**(4): p. 441-50.
476. Wang, L.P., et al., *BMP-7 attenuates liver fibrosis via regulation of epidermal growth factor receptor.* Int J Clin Exp Pathol, 2014. **7**(7): p. 3537-47.
477. Kinoshita, K., et al., *Adenovirus-mediated expression of BMP-7 suppresses the development of liver fibrosis in rats.* Gut, 2007. **56**(5): p. 706-14.
478. Hao, Z.M., et al., *Oral administration of recombinant adeno-associated virus-mediated bone morphogenetic protein-7 suppresses CCl(4)-induced hepatic fibrosis in mice.* Mol Ther, 2012. **20**(11): p. 2043-51.
479. Saika, S., et al., *Adenoviral gene transfer of BMP-7, Id2, or Id3 suppresses injury-induced epithelial-to-mesenchymal transition of lens epithelium in mice.* Am J Physiol Cell Physiol, 2006. **290**(1): p. C282-9.
480. Myllarniemi, M., et al., *Gremlin-mediated decrease in bone morphogenetic protein signaling promotes pulmonary fibrosis.* Am J Respir Crit Care Med, 2008. **177**(3): p. 321-9.
481. Yang, G., et al., *Bone morphogenetic protein 7 attenuates epithelial-mesenchymal transition induced by silica.* Hum Exp Toxicol, 2016. **35**(1): p. 69-77.
482. Yang, Y.L., et al., *Bone morphogenetic protein-2 antagonizes renal interstitial fibrosis by promoting catabolism of type I transforming growth factor-beta receptors.* Endocrinology, 2009. **150**(2): p. 727-40.
483. Klahr, S. and J. Morrissey, *Obstructive nephropathy and renal fibrosis: The role of bone morphogenic protein-7 and hepatocyte growth factor.* Kidney Int Suppl, 2003(87): p. S105-12.
484. Manson, S.R., et al., *The BMP-7-Smad1/5/8 pathway promotes kidney repair after obstruction induced renal injury.* J Urol, 2011. **185**(6 Suppl): p. 2523-30.
485. Manson, S.R., et al., *Endogenous BMP-7 is a critical molecular determinant of the reversibility of obstruction-induced renal injuries.* Am J Physiol Renal Physiol, 2011. **301**(6): p. F1293-302.
486. Vukicevic, S., et al., *Osteogenic protein-1 (bone morphogenetic protein-7) reduces severity of injury after ischemic acute renal failure in rat.* J Clin Invest, 1998. **102**(1): p. 202-14.
487. Zeisberg, M., et al., *Bone morphogenic protein-7 inhibits progression of chronic renal fibrosis associated with two genetic mouse models.* Am J Physiol Renal Physiol, 2003. **285**(6): p. F1060-7.
488. Sugimoto, H., et al., *Renal fibrosis and glomerulosclerosis in a new mouse model of diabetic nephropathy and its regression by bone morphogenic protein-7 and advanced glycation end product inhibitors.* Diabetes, 2007. **56**(7): p. 1825-33.
489. Li, R.X., et al., *BMP7 reduces inflammation and oxidative stress in diabetic tubulopathy.* Clin Sci (Lond), 2015. **128**(4): p. 269-80.
490. Zhen-Qiang, F., et al., *Localized expression of human BMP-7 by BM-MSCs enhances renal repair in an in vivo model of ischemia-reperfusion injury.* Genes Cells, 2012. **17**(1): p. 53-64.
491. Qi, R., W. Li, and S. Yu, *FK506 inhibits the mice glomerular mesangial cells proliferation by affecting the transforming growth factor-beta and Smads signal pathways.* Ren Fail, 2014. **36**(4): p. 589-92.
492. Zhang, Q., et al., *In vivo delivery of Gremlin siRNA plasmid reveals therapeutic potential against diabetic nephropathy by recovering bone morphogenetic protein-7.* PLoS One, 2010. **5**(7): p. e11709.
493. Liu, G.X., et al., *Smad7 inhibits AngII-mediated hypertensive nephropathy in a mouse model of hypertension.* Clin Sci (Lond), 2014. **127**(3): p. 195-208.
494. Sugimoto, H., et al., *Activin-like kinase 3 is important for kidney regeneration and reversal of fibrosis.* Nat Med, 2012. **18**(3): p. 396-404.
495. Manson, S.R., et al., *HDAC dependent transcriptional repression of Bmp-7 potentiates TGF-beta mediated renal fibrosis in obstructive uropathy.* J Urol, 2014. **191**(1): p. 242-52.
496. Cunha, S.I., et al., *Genetic and pharmacological targeting of activin receptor-like kinase 1 impairs tumor growth and angiogenesis.* J Exp Med, 2010. **207**(1): p. 85-100.
497. Bendell, J.C., et al., *Safety, pharmacokinetics, pharmacodynamics, and antitumor activity of dalantercept, an activin receptor-like kinase-1 ligand trap, in patients with advanced cancer.* Clin Cancer Res, 2014. **20**(2): p. 480-9.
498. Mitchell, D., et al., *ALK1-Fc inhibits multiple mediators of angiogenesis and suppresses tumor growth.* Mol Cancer Ther, 2010. **9**(2): p. 379-88.
499. Larrivee, B., et al., *ALK1 signaling inhibits angiogenesis by cooperating with the Notch pathway.* Dev Cell, 2012. **22**(3): p. 489-500.

500. Ricard, N., et al., *BMP9 and BMP10 are critical for postnatal retinal vascular remodeling.* Blood, 2012. **119**(25): p. 6162-71.
501. Wang, S., et al., *Up-regulation of BMP-2 antagonizes TGF-beta1/ROCK-enhanced cardiac fibrotic signalling through activation of Smurf1/Smad6 complex.* J Cell Mol Med, 2012. **16**(10): p. 2301-10.
502. Zeisberg, E.M., et al., *Endothelial-to-mesenchymal transition contributes to cardiac fibrosis.* Nat Med, 2007. **13**(8): p. 952-61.
503. Urbina, P. and D.K. Singla, *BMP-7 attenuates adverse cardiac remodeling mediated through M2 macrophages in prediabetic cardiomyopathy.* Am J Physiol Heart Circ Physiol, 2014. **307**(5): p. H762-72.
504. Wei, L.H., et al., *Smad7 inhibits angiotensin II-induced hypertensive cardiac remodelling.* Cardiovasc Res, 2013. **99**(4): p. 665-73.
505. Ebelt, H., et al., *Treatment with bone morphogenetic protein 2 limits infarct size after myocardial infarction in mice.* Shock, 2013. **39**(4): p. 353-60.
506. Sun, L., et al., *Bone morphogenetic protein-10 induces cardiomyocyte proliferation and improves cardiac function after myocardial infarction.* J Cell Biochem, 2014. **115**(11): p. 1868-76.
507. Pachori, A.S., et al., *Bone morphogenetic protein 4 mediates myocardial ischemic injury through JNK-dependent signaling pathway.* J Mol Cell Cardiol, 2010. **48**(6): p. 1255-65.
508. Ogura, Y., et al., *Therapeutic impact of follistatin-like 1 on myocardial ischemic injury in preclinical models.* Circulation, 2012. **126**(14): p. 1728-38.
509. Alfaro, M.P., et al., *sFRP2 suppression of bone morphogenic protein (BMP) and Wnt signaling mediates mesenchymal stem cell (MSC) self-renewal promoting engraftment and myocardial repair.* J Biol Chem, 2010. **285**(46): p. 35645-53.
510. Reynolds, A.M., et al., *Targeted gene delivery of BMPR2 attenuates pulmonary hypertension.* Eur Respir J, 2012. **39**(2): p. 329-43.
511. Reynolds, A.M., et al., *Bone morphogenetic protein type 2 receptor gene therapy attenuates hypoxic pulmonary hypertension.* Am J Physiol Lung Cell Mol Physiol, 2007. **292**(5): p. L1182-92.
512. McMurtry, M.S., et al., *Overexpression of human bone morphogenetic protein receptor 2 does not ameliorate monocrotaline pulmonary arterial hypertension.* Am J Physiol Lung Cell Mol Physiol, 2007. **292**(4): p. L872-8.
513. Feng, F., R.L. Harper, and P.N. Reynolds, *BMPR2 gene delivery reduces mutation-related PAH and counteracts TGF-beta-mediated pulmonary cell signalling.* Respirology, 2015.
514. Long, L., et al., *Selective enhancement of endothelial BMPR-II with BMP9 reverses pulmonary arterial hypertension.* Nat Med, 2015. **21**(7): p. 777-85.
515. Long, L., et al., *Chloroquine prevents progression of experimental pulmonary hypertension via inhibition of autophagy and lysosomal bone morphogenetic protein type II receptor degradation.* Circ Res, 2013. **112**(8): p. 1159-70.
516. Spiekerkoetter, E., et al., *FK506 activates BMPR2, rescues endothelial dysfunction, and reverses pulmonary hypertension.* J Clin Invest, 2013. **123**(8): p. 3600-13.
517. Ciuclan, L., et al., *Treatment with anti-gremlin 1 antibody ameliorates chronic hypoxia/SU5416-induced pulmonary arterial hypertension in mice.* Am J Pathol, 2013. **183**(5): p. 1461-73.
518. Brock, M., et al., *AntagomiR directed against miR-20a restores functional BMPR2 signalling and prevents vascular remodelling in hypoxia-induced pulmonary hypertension.* Eur Heart J, 2012.
519. Liu, Y., et al., *Serotonin induces Rho/ROCK-dependent activation of Smads 1/5/8 in pulmonary artery smooth muscle cells.* FASEB J, 2009. **23**(7): p. 2299-306.
520. Yang, J., et al., *Sildenafil potentiates bone morphogenetic protein signaling in pulmonary arterial smooth muscle cells and in experimental pulmonary hypertension.* Arterioscler Thromb Vasc Biol, 2013. **33**(1): p. 34-42.
521. Zhang, Y., et al., *Inhibition of bone morphogenic protein 4 restores endothelial function in db/db diabetic mice.* Arterioscler Thromb Vasc Biol, 2014. **34**(1): p. 152-9.
522. Koga, M., et al., *The bone morphogenic protein inhibitor, noggin, reduces glycemia and vascular inflammation in db/db mice.* Am J Physiol Heart Circ Physiol, 2013. **305**(5): p. H747-55.
523. Derwall, M., et al., *Inhibition of bone morphogenetic protein signaling reduces vascular calcification and atherosclerosis.* Arterioscler Thromb Vasc Biol, 2012. **32**(3): p. 613-22.
524. Malhotra, R., et al., *Inhibition of bone morphogenetic protein signal transduction prevents the medial vascular calcification associated with matrix Gla protein deficiency.* PLoS One, 2015. **10**(1): p. e0117098.

525. Kang, Y.H., et al., *Bone morphogenetic protein-7 inhibits vascular calcification induced by high vitamin D in mice.* Tohoku J Exp Med, 2010. **221**(4): p. 299-307.
526. Saeed, O., et al., *Pharmacological suppression of hepcidin increases macrophage cholesterol efflux and reduces foam cell formation and atherosclerosis.* Arterioscler Thromb Vasc Biol, 2012. **32**(2): p. 299-307.
527. Kajimoto, H., et al., *BMP type I receptor inhibition attenuates endothelial dysfunction in mice with chronic kidney disease.* Kidney Int, 2015. **87**(1): p. 128-36.
528. Park, S.S., et al., *Improvement of ovarian response and oocyte quality of aged female by administration of bone morphogenetic protein-6 in a mouse model.* Reprod Biol Endocrinol, 2012. **10**: p. 117.
529. Lee, W.S., et al., *Effect of bone morphogenetic protein-7 on folliculogenesis and ovulation in the rat.* Biol Reprod, 2001. **65**(4): p. 994-9.
530. McIntosh, C.J., et al., *Active immunization against the proregions of GDF9 or BMP15 alters ovulation rate and litter size in mice.* Reproduction, 2012. **143**(2): p. 195-201.
531. McNatty, K.P., et al., *The effects of immunizing sheep with different BMP15 or GDF9 peptide sequences on ovarian follicular activity and ovulation rate.* Biol Reprod, 2007. **76**(4): p. 552-60.
532. Juengel, J.L., et al., *Effects of immunization against bone morphogenetic protein 15 and growth differentiation factor 9 on ovulation rate, fertilization, and pregnancy in ewes.* Biol Reprod, 2004. **70**(3): p. 557-61.
533. Myllymaa, S., et al., *Inhibition of oocyte growth factors in vivo modulates ovarian folliculogenesis in neonatal and immature mice.* Reproduction, 2010. **139**(3): p. 587-98.
534. Jung, J.W., et al., *An Activin A/BMP2 chimera, AB215, blocks estrogen signaling via induction of ID proteins in breast cancer cells.* BMC Cancer, 2014. **14**: p. 549.
535. Ye, S., et al., *In vivo inhibition of bone morphogenetic protein-2 on breast cancer cell growth.* Spine (Phila Pa 1976), 2013. **38**(3): p. E143-50.
536. Molina, C.A., et al., *Delayed onset of paralysis and slowed tumor growth following in situ placement of recombinant human bone morphogenetic protein 2 within spine tumors in a rat model of metastatic breast cancer.* J Neurosurg Spine, 2012. **16**(4): p. 365-72.
537. Buijs, J.T., et al., *The BMP2/7 heterodimer inhibits the human breast cancer stem cell subpopulation and bone metastases formation.* Oncogene, 2012. **31**(17): p. 2164-74.
538. Owens, P., et al., *Inhibition of BMP signaling suppresses metastasis in mammary cancer.* Oncogene, 2015. **34**(19): p. 2437-49.
539. Li, Q., et al., *Mesenchymal stem cells from human fat engineered to secrete BMP4 are nononcogenic, suppress brain cancer, and prolong survival.* Clin Cancer Res, 2014. **20**(9): p. 2375-87.
540. Ye, X.Y., et al., *Adenovirus mediated knockdown of bone morphogenetic protein 2 inhibits human lung cancer growth and invasion in vitro and in vivo.* Int J Immunopathol Pharmacol, 2012. **25**(4): p. 967-76.
541. Dai, J., et al., *Bone morphogenetic protein-6 promotes osteoblastic prostate cancer bone metastases through a dual mechanism.* Cancer Res, 2005. **65**(18): p. 8274-85.
542. Kobayashi, A., et al., *Bone morphogenetic protein 7 in dormancy and metastasis of prostate cancer stem-like cells in bone.* J Exp Med, 2011. **208**(13): p. 2641-55.
543. Lee, Y.C., et al., *BMP4 promotes prostate tumor growth in bone through osteogenesis.* Cancer Res, 2011. **71**(15): p. 5194-203.
544. Virk, M.S., et al., *Combined inhibition of the BMP pathway and the RANK-RANKL axis in a mixed lytic/blastic prostate cancer lesion.* Bone, 2011. **48**(3): p. 578-87.
545. Virk, M.S., et al., *Influence of simultaneous targeting of the bone morphogenetic protein pathway and RANK/RANKL axis in osteolytic prostate cancer lesion in bone.* Bone, 2009. **44**(1): p. 160-7.
546. Feeley, B.T., et al., *Overexpression of noggin inhibits BMP-mediated growth of osteolytic prostate cancer lesions.* Bone, 2006. **38**(2): p. 154-66.
547. Secondini, C., et al., *The role of the BMP signaling antagonist noggin in the development of prostate cancer osteolytic bone metastasis.* PLoS One, 2011. **6**(1): p. e16078.
548. Hawinkels, L.J., et al., *Activin Receptor-like Kinase 1 Ligand Trap Reduces Microvascular Density and Improves Chemotherapy Efficiency to Various Solid Tumors.* Clin Cancer Res, 2016. **22**(1): p. 96-106.
549. Dummula, K., et al., *Bone morphogenetic protein inhibition promotes neurological recovery after intraventricular hemorrhage.* J Neurosci, 2011. **31**(34): p. 12068-82.
550. Pei, H., et al., *Bone morphogenetic protein-7 ameliorates cerebral ischemia and reperfusion injury via inhibiting oxidative stress and neuronal apoptosis.* Int J Mol Sci, 2013. **14**(12): p. 23441-53.

551. Xu, J.H., et al., *Protective effects of recombinant human bone morphogenetic protein-7 on focal cerebral ischemia-reperfusion injury.* Int J Neurosci, 2013. **123**(6): p. 375-84.
552. Liu, Y., et al., *The effect of bone morphogenetic protein-7 (BMP-7) on functional recovery, local cerebral glucose utilization and blood flow after transient focal cerebral ischemia in rats.* Brain Res, 2001. **905**(1-2): p. 81-90.
553. Guan, J., et al., *Bone morphogenetic protein-7 (BMP-7) mediates ischemic preconditioning-induced ischemic tolerance via attenuating apoptosis in rat brain.* Biochem Biophys Res Commun, 2013. **441**(3): p. 560-6.
554. Heinonen, A.M., et al., *Neuroprotection by rAAV-mediated gene transfer of bone morphogenic protein 7.* BMC Neurosci, 2014. **15**: p. 38.
555. Shin, J.A., et al., *Noggin improves ischemic brain tissue repair and promotes alternative activation of microglia in mice.* Brain Behav Immun, 2014.
556. Ding, J., et al., *Effects of nerve growth factor and Noggin-modified bone marrow stromal cells on stroke in rats.* J Neurosci Res, 2011. **89**(2): p. 222-30.
557. Chen, C., Y. Cheng, and J. Chen, *Transfection of Noggin in bone marrow stromal cells (BMSCs) enhances BMSC-induced functional outcome after stroke in rats.* J Neurosci Res, 2011. **89**(8): p. 1194-202.
558. Sabo, J.K., et al., *Remyelination is altered by bone morphogenic protein signaling in demyelinated lesions.* J Neurosci, 2011. **31**(12): p. 4504-10.
559. Sabo, J.K., et al., *Investigation of sequential growth factor delivery during cuprizone challenge in mice aimed to enhance oligodendrogliogenesis and myelin repair.* PLoS One, 2013. **8**(5): p. e63415.
560. Hampton, D.W., et al., *A potential role for bone morphogenetic protein signalling in glial cell fate determination following adult central nervous system injury in vivo.* Eur J Neurosci, 2007. **26**(11): p. 3024-35.
561. Hampton, D.W., et al., *Spinally upregulated noggin suppresses axonal and dendritic plasticity following dorsal rhizotomy.* Exp Neurol, 2007. **204**(1): p. 366-79.
562. Matsuura, I., et al., *BMP inhibition enhances axonal growth and functional recovery after spinal cord injury.* J Neurochem, 2008. **105**(4): p. 1471-9.
563. Xiao, Q., et al., *Bone morphogenetic proteins mediate cellular response and, together with Noggin, regulate astrocyte differentiation after spinal cord injury.* Exp Neurol, 2010. **221**(2): p. 353-66.
564. Setoguchi, T., et al., *Treatment of spinal cord injury by transplantation of fetal neural precursor cells engineered to express BMP inhibitor.* Exp Neurol, 2004. **189**(1): p. 33-44.
565. Li, Z., et al., *Bone morphogenetic protein 4 inhibits liposaccharide-induced inflammation in the airway.* Eur J Immunol, 2014. **44**(11): p. 3283-94.
566. Wang, L., et al., *The bone morphogenetic protein-hepcidin axis as a therapeutic target in inflammatory bowel disease.* Inflamm Bowel Dis, 2012. **18**(1): p. 112-9.
567. Maric, I., et al., *Bone morphogenetic protein-7 reduces the severity of colon tissue damage and accelerates the healing of inflammatory bowel disease in rats.* J Cell Physiol, 2003. **196**(2): p. 258-64.
568. Maric, I., et al., *BMP signaling in rats with TNBS-induced colitis following BMP7 therapy.* Am J Physiol Gastrointest Liver Physiol, 2012. **302**(10): p. G1151-62.
569. Steinbicker, A.U., et al., *Inhibition of bone morphogenetic protein signaling attenuates anemia associated with inflammation.* Blood, 2011. **117**(18): p. 4915-23.
570. Andriopoulos, B., Jr., et al., *BMP6 is a key endogenous regulator of hepcidin expression and iron metabolism.* Nat Genet, 2009. **41**(4): p. 482-7.
571. Meynard, D., et al., *Regulation of TMPRSS6 by BMP6 and iron in human cells and mice.* Blood, 2011. **118**(3): p. 747-56.
572. Yu, P.B., et al., *Dorsomorphin inhibits BMP signals required for embryogenesis and iron metabolism.* Nat Chem Biol, 2008. **4**(1): p. 33-41.
573. Poli, M., et al., *Heparin: a potent inhibitor of hepcidin expression in vitro and in vivo.* Blood, 2011. **117**(3): p. 997-1004.
574. Theurl, I., et al., *Pharmacologic inhibition of hepcidin expression reverses anemia of chronic inflammation in rats.* Blood, 2011. **118**(18): p. 4977-84.
575. Babitt, J.L., et al., *Modulation of bone morphogenetic protein signaling in vivo regulates systemic iron balance.* J Clin Invest, 2007. **117**(7): p. 1933-9.
576. Tsugawa, D., et al., *Specific activin receptor-like kinase 3 inhibitors enhance liver regeneration.* J Pharmacol Exp Ther, 2014. **351**(3): p. 549-58.

577. Helbing, T., et al., *Inhibition of BMP activity protects epithelial barrier function in lung injury.* J Pathol, 2013. **231**(1): p. 105-16.
578. Shi, S., et al., *BMP antagonists enhance myogenic differentiation and ameliorate the dystrophic phenotype in a DMD mouse model.* Neurobiol Dis, 2011. **41**(2): p. 353-60.
579. Boon, M.R., et al., *BMP7 activates brown adipose tissue and reduces diet-induced obesity only at subthermoneutrality.* PLoS One, 2013. **8**(9): p. e74083.
580. Cao, Y., et al., *Noggin attenuates cerulein-induced acute pancreatitis and impaired autophagy.* Pancreas, 2013. **42**(2): p. 301-7.
581. Ueki, Y. and T.A. Reh, *Activation of BMP-Smad1/5/8 signaling promotes survival of retinal ganglion cells after damage in vivo.* PLoS One, 2012. **7**(6): p. e38690.
582. Yoon, B.H., et al., *Anti-wrinkle effect of bone morphogenetic protein receptor 1a-extracellular domain (BMPR1a-ECD).* BMB Rep, 2013. **46**(9): p. 465-70.
583. Anderson, B.C., et al., *Sustained release of bone morphogenetic protein-4 in adult rabbit extraocular muscle results in decreased force and muscle size: potential for strabismus treatment.* Invest Ophthalmol Vis Sci, 2011. **52**(7): p. 4021-9.
584. Kihara, T., et al., *Acerogenin A, a natural compound isolated from Acer nikoense Maxim, stimulates osteoblast differentiation through bone morphogenetic protein action.* Biochem Biophys Res Commun, 2011. **406**(2): p. 211-7.
585. Li, X., et al., *Identification of upregulators of BMP2 expression via high-throughput screening of a synthetic and natural compound library.* J Biomol Screen, 2009. **14**(10): p. 1251-6.
586. Kong, X.H., et al., *Astragaloside II induces osteogenic activities of osteoblasts through the bone morphogenetic protein-2/MAPK and Smad1/5/8 pathways.* Int J Mol Med, 2012. **29**(6): p. 1090-8.
587. Lee, K.B., et al., *Bone morphogenetic protein-binding peptide reduces the inflammatory response to recombinant human bone morphogenetic protein-2 and recombinant human bone morphogenetic protein-7 in a rodent model of soft-tissue inflammation.* Spine J, 2011. **11**(6): p. 568-76.
588. Taghavi, C.E., et al., *Bone morphogenetic protein binding peptide mechanism and enhancement of osteogenic protein-1 induced bone healing.* Spine (Phila Pa 1976), 2010. **35**(23): p. 2049-56.
589. Dunmore, B.J., et al., *The lysosomal inhibitor, chloroquine, increases cell surface BMPR-II levels and restores BMP9 signalling in endothelial cells harbouring BMPR-II mutations.* Hum Mol Genet, 2013. **22**(18): p. 3667-79.
590. Munisso, M.C., et al., *Cilomilast enhances osteoblast differentiation of mesenchymal stem cells and bone formation induced by bone morphogenetic protein 2.* Biochimie, 2012. **94**(11): p. 2360-5.
591. Guo, H.F., et al., *Substituted benzothiophene or benzofuran derivatives as a novel class of bone morphogenetic protein-2 up-regulators: synthesis, structure-activity relationships, and preventive bone loss efficacies in senescence accelerated mice (SAMP6) and ovariectomized rats.* J Med Chem, 2010. **53**(4): p. 1819-29.
592. Lee, H.R., et al., *Discovery of a Small Molecule that Enhances Astrocytogenesis by Activation of STAT3, SMAD1/5/8, and ERK1/2 via Induction of Cytokines in Neural Stem Cells.* ACS Chem Neurosci, 2015.
593. Balaramnavar, V.M., et al., *Identification of novel 2-((1-(benzyl(2-hydroxy-2-phenylethyl)amino)-1-oxo-3-phenylpropan-2-yl)carbamoyl) benzoic acid analogues as BMP-2 stimulators.* J Med Chem, 2012. **55**(19): p. 8248-59.
594. Tang, C.H., et al., *Enhancement of bone morphogenetic protein-2 expression and bone formation by coumarin derivatives via p38 and ERK-dependent pathway in osteoblasts.* Eur J Pharmacol, 2008. **579**(1-3): p. 40-9.
595. Lee, S.Y., et al., *Combined effects of dentin sialoprotein and bone morphogenetic protein-2 on differentiation in human cementoblasts.* Cell Tissue Res, 2014. **357**(1): p. 119-32.
596. Uemura, T., et al., *Epinephrine accelerates osteoblastic differentiation by enhancing bone morphogenetic protein signaling through a cAMP/protein kinase A signaling pathway.* Bone, 2010. **47**(4): p. 756-65.
597. Matsumoto, Y., et al., *Estrogen facilitates osteoblast differentiation by upregulating bone morphogenetic protein-4 signaling.* Steroids, 2013. **78**(5): p. 513-20.
598. Lee, T.C., et al., *Bone morphogenetic protein-2 expression in spinal fusion masses enhanced by extracorporeal shock wave treatment: a rabbit experiment.* Acta Neurochir (Wien), 2010. **152**(10): p. 1779-84.

599. Kanazawa, I., et al., *Fasudil hydrochloride induces osteoblastic differentiation of stromal cell lines, C3H10T1/2 and ST2, via bone morphogenetic protein-2 expression.* Endocr J, 2010. **57**(5): p. 415-21.
600. Kanazawa, I., et al., *Activation of AMP kinase and inhibition of Rho kinase induce the mineralization of osteoblastic MC3T3-E1 cells through endothelial NOS and BMP-2 expression.* Am J Physiol Endocrinol Metab, 2009. **296**(1): p. E139-46.
601. Roedel, E.K., E. Schwarz, and S.M. Kanse, *The factor VII-activating protease (FSAP) enhances the activity of bone morphogenetic protein-2 (BMP-2).* J Biol Chem, 2013. **288**(10): p. 7193-203.
602. Dai, J., et al., *Genistein promotion of osteogenic differentiation through BMP2/SMAD5/RUNX2 signaling.* Int J Biol Sci, 2013. **9**(10): p. 1089-98.
603. Yonezawa, T., et al., *Harmine promotes osteoblast differentiation through bone morphogenetic protein signaling.* Biochem Biophys Res Commun, 2011. **409**(2): p. 260-5.
604. Hu, N., et al., *Inhibition of histone deacetylases potentiates BMP9-induced osteogenic signaling in mouse mesenchymal stem cells.* Cell Physiol Biochem, 2013. **32**(2): p. 486-98.
605. Kuo, W.J., M.A. Digman, and A.D. Lander, *Heparan sulfate acts as a bone morphogenetic protein coreceptor by facilitating ligand-induced receptor hetero-oligomerization.* Mol Biol Cell, 2010. **21**(22): p. 4028-41.
606. Imai, Y., et al., *Hepatocyte growth factor contributes to fracture repair by upregulating the expression of BMP receptors.* J Bone Miner Res, 2005. **20**(10): p. 1723-30.
607. Joung, Y.H., et al., *Hwanggeumchal sorghum extract enhances BMP7 and GH signaling through the activation of Jak2/STAT5B in MC3T3E1 osteoblastic cells.* Mol Med Rep, 2013. **8**(3): p. 891-6.
608. Yao, Y., et al., *Heat shock protein 70 enhances vascular bone morphogenetic protein-4 signaling by binding matrix Gla protein.* Circ Res, 2009. **105**(6): p. 575-84.
609. Liang, W., et al., *Icariin promotes bone formation via the BMP-2/Smad4 signal transduction pathway in the hFOB 1.19 human osteoblastic cell line.* Int J Mol Med, 2012. **30**(4): p. 889-95.
610. Shi, S., et al., *Regulation of articular chondrocyte aggrecan and collagen gene expression by multiple growth factor gene transfer.* J Orthop Res, 2012. **30**(7): p. 1026-31.
611. Chen, L., et al., *Insulin-like growth factor 2 (IGF-2) potentiates BMP-9-induced osteogenic differentiation and bone formation.* J Bone Miner Res, 2010. **25**(11): p. 2447-59.
612. Kautz, L., et al., *Iron regulates phosphorylation of Smad1/5/8 and gene expression of Bmp6, Smad7, Id1, and Atoh8 in the mouse liver.* Blood, 2008. **112**(4): p. 1503-9.
613. Ellman, M.B., et al., *Lactoferricin enhances BMP7-stimulated anabolic pathways in intervertebral disc cells.* Gene, 2013. **524**(2): p. 282-91.
614. Zhang, C., et al., *Lactoferrin activates BMP7 gene expression through the mitogen-activated protein kinase ERK pathway in articular cartilage.* Biochem Biophys Res Commun, 2013. **431**(1): p. 31-5.
615. Kim, S.N., et al., *In vitro and in vivo osteogenic activity of licochalcone A.* Amino Acids, 2012. **42**(4): p. 1455-65.
616. Yang, Z., et al., *Low-intensity pulsed ultrasound induces osteogenic differentiation of human periodontal ligament cells through activation of bone morphogenetic protein-smad signaling.* J Ultrasound Med, 2014. **33**(5): p. 865-73.
617. Xue, H., et al., *Low-intensity pulsed ultrasound accelerates tooth movement via activation of the BMP-2 signaling pathway.* PLoS One, 2013. **8**(7): p. e68926.
618. Hou, C.H., S.M. Hou, and C.H. Tang, *Ultrasound increased BMP-2 expression via PI3K, Akt, c-Fos/c-Jun, and AP-1 pathways in cultured osteoblasts.* J Cell Biochem, 2009. **106**(1): p. 7-15.
619. Huang, W., et al., *Low-intensity pulsed ultrasound enhances bone morphogenetic protein expression of human mandibular fracture haematoma-derived cells.* Int J Oral Maxillofac Surg, 2015. **44**(7): p. 929-35.
620. Hirata, S., et al., *Low-level laser irradiation enhances BMP-induced osteoblast differentiation by stimulating the BMP/Smad signaling pathway.* J Cell Biochem, 2010. **111**(6): p. 1445-52.
621. Kodach, L.L., et al., *The effect of statins in colorectal cancer is mediated through the bone morphogenetic protein pathway.* Gastroenterology, 2007. **133**(4): p. 1272-81.
622. Nam, J., et al., *Dynamic regulation of bone morphogenetic proteins in engineered osteochondral constructs by biomechanical stimulation.* Tissue Eng Part A, 2013. **19**(5-6): p. 783-92.
623. Rath, B., et al., *Biomechanical forces exert anabolic effects on osteoblasts by activation of SMAD 1/5/8 through type 1 BMP receptor.* Biorheology, 2011. **48**(1): p. 37-48.
624. Balachandran, K., et al., *Elevated cyclic stretch induces aortic valve calcification in a bone morphogenic protein-dependent manner.* Am J Pathol, 2010. **177**(1): p. 49-57.

625. Park, K.H., et al., *Melatonin promotes osteoblastic differentiation through the BMP/ERK/Wnt signaling pathways.* J Pineal Res, 2011. **51**(2): p. 187-94.
626. Hsu, Y.L., et al., *Myricetin induces human osteoblast differentiation through bone morphogenetic protein-2/p38 mitogen-activated protein kinase pathway.* Biochem Pharmacol, 2007. **73**(4): p. 504-14.
627. Miguel, B.S., et al., *N-methyl pyrrolidone as a potent bone morphogenetic protein enhancer for bone tissue regeneration.* Tissue Eng Part A, 2009. **15**(10): p. 2955-63.
628. Tan, T.W., et al., *CCN3 increases BMP-4 expression and bone mineralization in osteoblasts.* J Cell Physiol, 2012. **227**(6): p. 2531-41.
629. Nakagawa, K., et al., *Prostaglandin E2 EP4 agonist (ONO-4819) accelerates BMP-induced osteoblastic differentiation.* Bone, 2007. **41**(4): p. 543-8.
630. Tanaka, M., et al., *Prostaglandin E2 receptor (EP4) selective agonist (ONO-4819.CD) accelerates bone repair of femoral cortex after drill-hole injury associated with local upregulation of bone turnover in mature rats.* Bone, 2004. **34**(6): p. 940-8.
631. Yu, B., et al., *Parathyroid hormone induces differentiation of mesenchymal stromal/stem cells by enhancing bone morphogenetic protein signaling.* J Bone Miner Res, 2012. **27**(9): p. 2001-14.
632. Zhang, R., et al., *Transcriptional Regulation of BMP2 Expression by the PTH-CREB Signaling Pathway in Osteoblasts.* PloS one, 2011. **6**(6): p. e20780.
633. Nakao, Y., et al., *Parathyroid hormone enhances bone morphogenetic protein activity by increasing intracellular 3', 5'-cyclic adenosine monophosphate accumulation in osteoblastic MC3T3-E1 cells.* Bone, 2009. **44**(5): p. 872-7.
634. Park, K.W., et al., *The small molecule phenamil induces osteoblast differentiation and mineralization.* Mol Cell Biol, 2009. **29**(14): p. 3905-14.
635. Ying, X., et al., *Phosphoserine promotes osteogenic differentiation of human adipose stromal cells through bone morphogenetic protein signalling.* Cell Biol Int, 2014. **38**(3): p. 309-17.
636. Okada, M., et al., *Upregulation of intervertebral disc-cell matrix synthesis by pulsed electromagnetic field is mediated by bone morphogenetic proteins.* J Spinal Disord Tech, 2013. **26**(3): p. 167-73.
637. Jansen, J.H., et al., *Stimulation of osteogenic differentiation in human osteoprogenitor cells by pulsed electromagnetic fields: an in vitro study.* BMC Musculoskelet Disord, 2010. **11**: p. 188.
638. Schwartz, Z., et al., *Pulsed electromagnetic fields enhance BMP-2 dependent osteoblastic differentiation of human mesenchymal stem cells.* J Orthop Res, 2008. **26**(9): p. 1250-5.
639. Casarin, R.C., et al., *Resveratrol improves bone repair by modulation of bone morphogenetic proteins and osteopontin gene expression in rats.* Int J Oral Maxillofac Surg, 2014. **43**(7): p. 900-6.
640. Kuroyanagi, G., et al., *Resveratrol amplifies BMP-4-stimulated osteoprotegerin synthesis via p38 MAP kinase in osteoblasts.* Mol Med Rep, 2015. **12**(3): p. 3849-54.
641. Reiner, D.J., et al., *9-Cis retinoic acid protects against methamphetamine-induced neurotoxicity in nigrostriatal dopamine neurons.* Neurotox Res, 2014. **25**(3): p. 248-61.
642. Shen, H., et al., *9-Cis-retinoic acid reduces ischemic brain injury in rodents via bone morphogenetic protein.* J Neurosci Res, 2009. **87**(2): p. 545-55.
643. Karakida, T., et al., *Retinoic acid receptor gamma-dependent signaling cooperates with BMP2 to induce osteoblastic differentiation of C2C12 cells.* Connect Tissue Res, 2011. **52**(5): p. 365-72.
644. Moore, N.M., et al., *Synergistic enhancement of human bone marrow stromal cell proliferation and osteogenic differentiation on BMP-2-derived and RGD peptide concentration gradients.* Acta Biomater, 2011. **7**(5): p. 2091-100.
645. Chen, J.J., et al., *Salidroside stimulates osteoblast differentiation through BMP signaling pathway.* Food Chem Toxicol, 2013. **62**: p. 499-505.
646. Rondelet, B., et al., *Sildenafil added to sitaxsentan in overcirculation-induced pulmonary arterial hypertension.* Am J Physiol Heart Circ Physiol, 2010. **299**(4): p. H1118-23.
647. Yen, C.H., et al., *Sildenafil limits monocrotaline-induced pulmonary hypertension in rats through suppression of pulmonary vascular remodeling.* J Cardiovasc Pharmacol, 2010. **55**(6): p. 574-84.
648. Yang, L., et al., *Sildenefil increases connexin 40 in smooth muscle cells through activation of BMP pathways in pulmonary arterial hypertension.* Int J Clin Exp Pathol, 2014. **7**(8): p. 4674-84.
649. Ying, X., et al., *Silibinin promotes osteoblast differentiation of human bone marrow stromal cells via bone morphogenetic protein signaling.* Eur J Pharmacol, 2013. **721**(1-3): p. 225-30.
650. Zhang, H. and C.Y. Lin, *Simvastatin stimulates chondrogenic phenotype of intervertebral disc cells partially through BMP-2 pathway.* Spine (Phila Pa 1976), 2008. **33**(16): p. E525-31.

651. Bradley, J.D., et al., *Cyclooxygenase-2 inhibitor reduces simvastatin-induced bone morphogenetic protein-2 and bone formation in vivo.* J Periodontal Res, 2007. **42**(3): p. 267-73.
652. Song, C., et al., *Simvastatin induces osteoblastic differentiation and inhibits adipocytic differentiation in mouse bone marrow stromal cells.* Biochem Biophys Res Commun, 2003. **308**(3): p. 458-62.
653. Maeda, T., et al., *Simvastatin promotes osteoblast differentiation and mineralization in MC3T3-E1 cells.* Biochem Biophys Res Commun, 2001. **280**(3): p. 874-7.
654. Sugiyama, M., et al., *Compactin and simvastatin, but not pravastatin, induce bone morphogenetic protein-2 in human osteosarcoma cells.* Biochem Biophys Res Commun, 2000. **271**(3): p. 688-92.
655. Cao, Y., et al., *Selective small molecule compounds increase BMP-2 responsiveness by inhibiting Smurf1-mediated Smad1/5 degradation.* Sci Rep, 2014. **4**: p. 4965.
656. Kato, S., et al., *A synthetic compound that potentiates bone morphogenetic protein-2-induced transdifferentiation of myoblasts into the osteoblastic phenotype.* Mol Cell Biochem, 2011. **349**(1-2): p. 97-106.
657. Okada, M., et al., *Development and optimization of a cell-based assay for the selection of synthetic compounds that potentiate bone morphogenetic protein-2 activity.* Cell Biochem Funct, 2009. **27**(8): p. 526-34.
658. Hsu, Y.L., et al., *Syringetin, a flavonoid derivative in grape and wine, induces human osteoblast differentiation through bone morphogenetic protein-2/extracellular signal-regulated kinase 1/2 pathway.* Mol Nutr Food Res, 2009. **53**(11): p. 1452-61.
659. de Gorter, D.J., et al., *Biphasic effects of transforming growth factor beta on bone morphogenetic protein-induced osteoblast differentiation.* J Bone Miner Res, 2011. **26**(6): p. 1178-87.
660. Simmons, C.A., et al., *Dual growth factor delivery and controlled scaffold degradation enhance in vivo bone formation by transplanted bone marrow stromal cells.* Bone, 2004. **35**(2): p. 562-9.
661. Sun, S.X., et al., *BMP-2 and titanium particles synergistically activate osteoclast formation.* Braz J Med Biol Res, 2014. **47**(6): p. 461-9.
662. Xu, L., et al., *U0126 promotes osteogenesis of rat bone-marrow-derived mesenchymal stem cells by activating BMP/Smad signaling pathway.* Cell Tissue Res, 2015. **359**(2): p. 537-45.
663. Damrongsri, D., et al., *Cyclooxygenase-2 inhibition selectively attenuates bone morphogenetic protein-6 synthesis and bone formation during guided tissue regeneration in a rat model.* Clin Oral Implants Res, 2006. **17**(1): p. 38-47.
664. Song, T., et al., *Fibroblast growth factor 2 inhibits bone morphogenetic protein 9-induced osteogenic differentiation of mesenchymal stem cells by repressing Smads signaling and subsequently reducing Smads dependent up-regulation of ALK1 and ALK2.* Int J Biochem Cell Biol, 2013. **45**(8): p. 1639-46.
665. Wahdan-Alaswad, R.S., et al., *Insulin-like growth factor I suppresses bone morphogenetic protein signaling in prostate cancer cells by activating mTOR signaling.* Cancer Res, 2010. **70**(22): p. 9106-17.
666. Brock, M., et al., *Interleukin-6 modulates the expression of the bone morphogenic protein receptor type II through a novel STAT3-microRNA cluster 17/92 pathway.* Circ Res, 2009. **104**(10): p. 1184-91.
667. Huang, R.L., et al., *LPS-stimulated inflammatory environment inhibits BMP-2-induced osteoblastic differentiation through crosstalk between TLR4/MyD88/NF-kappaB and BMP/Smad signaling.* Stem Cells Dev, 2014. **23**(3): p. 277-89.
668. Kodama, A., et al., *Progesterone decreases bone morphogenetic protein (BMP) 7 expression and BMP7 inhibits decidualization and proliferation in endometrial stromal cells.* Hum Reprod, 2010. **25**(3): p. 751-6.
669. Mi, D., et al., *PTHrP inhibits BMP-6 expression through the PKA signaling pathway in breast cancer cells.* J Cancer Res Clin Oncol, 2011. **137**(2): p. 295-303.
670. Yeh, L.C., et al., *Rapamycin inhibits BMP-7-induced osteogenic and lipogenic marker expressions in fetal rat calvarial cells.* J Cell Biochem, 2013. **114**(8): p. 1760-71.
671. Sheng, N., et al., *Retinoic acid regulates bone morphogenic protein signal duration by promoting the degradation of phosphorylated Smad1.* Proc Natl Acad Sci U S A, 2010. **107**(44): p. 18886-91.
